# Disentangling influence over group speed and direction reveals multiple patterns of influence in moving meerkat groups

**DOI:** 10.1038/s41598-022-17259-z

**Published:** 2022-08-16

**Authors:** Baptiste Averly, Vivek H. Sridhar, Vlad Demartsev, Gabriella Gall, Marta Manser, Ariana Strandburg-Peshkin

**Affiliations:** 1 Department for the Ecology of Animal Societies, Max Planck Institute of Animal Behavior, Constance, Germany; 2 Department of Biology, University of Konstanz, Constance, Germany; 3 Centre for the Advanced Study of Collective Behaviour, University of Konstanz, Constance, Germany; 4 Kalahari Meerkat Project, Kuruman River Reserve, Northern Cape, South Africa; 5 Department of Evolutionary Biology and Environmental Studies, University of Zurich, Zurich, Switzerland; 6 Zukunftskolleg, University of Konstanz, Constance, Germany; 7 Mammal Research Institute, University of Pretoria, Pretoria, South Africa

**Keywords:** Animal behaviour, Behavioural ecology

## Abstract

Animals that travel together in groups must constantly come to consensus about both the direction and speed of movement, often simultaneously. Contributions to collective decisions may vary among group members, yet inferring who has influence over group decisions is challenging, largely due to the multifaceted nature of influence. Here we collected high-resolution GPS data from five habituated meerkat groups in their natural habitat during foraging and developed a method to quantify individual influence over both group direction and speed. We find that individual influence over direction and speed are correlated, but also exhibit substantial variation. Comparing patterns across social statuses reveals that dominant females have higher influence than other individuals over both group direction and speed. Individuals with high influence also tend to spend more time in the front of the group. We discuss our results in light of meerkat life-history and current literature on influence during group movement. Our method provides a general approach which can be applied to disentangle individual influence over group direction and speed in a wide range of species with cohesive movement, emphasizing the importance of integrating multiple lines of inquiry when inferring influence in moving animal groups.

## Introduction

In social animals, individuals often make differential contributions to group decisions. Group members whose actions cause others to change their behavior can be said to exert *influence*, and the distribution and consistency of influence among group-mates can vary across decision types, contexts, and species^1^. Group decision mechanisms can range from completely shared, where most individuals contribute to most decisions, to completely unshared, where one or a few individuals consistently impose their preferences on other group members^2^. Studies of influence on collective movement have generally focused on single dimensions of influence, such as who leads from the front of the group^3–6^, or who determines the group direction^7–11^ and timing of travel^12,13^. A positive link between influence and social rank has often been established (reviewed in Smith et al), though systems with influential subordinates are also found^14–16^. Thus, there is a diversity of movement decision-making mechanisms in nature depending on group characteristics such as size and composition, social structure, and type of movement^17–19^.

In part because of this diversity, assessing patterns of influence and comparing them between social systems remains challenging. In order to correctly define and quantify influence in a social system, one should ideally first identify the decision-making mechanisms at play and the individual actions that impact behavioral decisions of others^1^. In the context of movement, these actions can include an individual’s position in space, its movement in a given direction, or the production of signals such as vocalizations. To assess whether individuals differ in their influence, one then needs to quantify whether groups respond differently to the behaviors of different individuals. In most cases, it is difficult or impossible to firmly establish causal relationships underlying how the behavior of individuals translates into group-level outcomes, especially in natural (unmanipulated) settings. However, by analyzing recurring patterns in the temporal sequence of events (for example observing the probability that a group leaves a resting spot after an individual initiates movement), it is possible to establish reasonable proxies of individual influence over group decision-making (for a review, see^1^).

How much influence an individual exerts may also vary depending on the type of decision being made. In particular, theoretical work has emphasized a fundamental distinction between decisions about movement *direction* and decisions about movement *timing*. These two types of decision are expected to have different distributions of consensus costs, leading to contrasting predictions about whether they are likely to be shared or unshared^20,21^. It can be particularly challenging to disentangle these two fields of influence, as both may occur at the same time when groups travel collectively, continuously needing to come to consensus on both the direction and speed of travel. Very few field studies have simultaneously looked at multiple measures of influence in the same system, to evaluate if influence in one domain necessarily translates to influence in other domains (but see^22–24^ for lab experiments). The versatility of the notion of influence makes it crucial to define the context in which it is assessed, while also accounting for the biology and social structure of the study system.

We investigated patterns of influence over group movement in meerkats (*Suricata suricatta)*, a social mongoose species that lives in highly cohesive groups of up to 50 individuals, in the arid parts of southern Africa^25,26^. Meerkats are opportunistic generalists that forage on small invertebrate and some vertebrate prey distributed across their desert habitat by digging in the ground^27^. The dispersed nature of prey is reflected in the groups’ movement dynamics: throughout the day meerkat groups typically move in a relatively slow, continuous fashion while simultaneously foraging. Though individuals forage independently, typically 1–10 m from their nearest neighbors^28^, groups remain highly cohesive while navigating 2–5 km^2^ territories^29^. Meerkats have a highly developed vocal repertoire^30^ and calls have been shown to play an important role in maintaining cohesion^31,32^ and in initiating rapid travel when relocating^15^, returning to a sleeping burrow^33^, or as a predator avoidance response^34^. Yet, the extent to which different group members influence collective decisions about movement speed and direction remains unclear. Meerkat groups are socially structured with a dominant pair monopolizing most of the breeding opportunities^35,36^, and much weaker social hierarchies among subordinate group members^37^. Once established, dominant females (normally the eldest natal members of their groups) typically retain their status for life using behavioral assertions of dominance including reproductive suppression and aggression toward subordinate members, sometimes resulting in eviction^38–40^. Dominant males are typically non-natal and have a shorter tenure, though the majority still retain their position for more than a year^26^. Thus far, however, there is little evidence that dominance translates into stronger influence over group movement decisions^33,41–43^.

Here, we assess the distribution of influence over collective movement decisions in foraging meerkats in their natural habitat using high-resolution GPS data from five social groups of varying sizes. We develop a method for disentangling individual influence over the speed and direction of moving groups, which is widely applicable across animal study systems. We use this method to assess if patterns of influence are associated with social status within groups, as well as whether the two different domains of influence correlate with one another. Since frontmost position is often used as a proxy for influence^5,6,44^, we also test whether individuals that spend more time in the front of the group have higher levels of influence as assessed by our method.

## Methods

### Study site and data collection

#### Study system

The study was conducted at the Kalahari Meerkat Project (KMP) within the Kuruman River Reserve in South Africa (26° 58′ S, 21° 49′ E)^45^, where 7–15 habituated meerkat groups are continuously monitored for group composition, dominance status, and life history events. We collected simultaneous, high-resolution movement data on the majority of individuals within five meerkat groups (Table S1): HM17 (7 individuals) in August and September 2017, HM19 (18 individuals) in June and July 2019, L19 (19 individuals) in August 2019, ZU21 (13 individuals) in May 2021 and NQ21 (11 individuals) in August 2021. We chose the groups with the highest levels of habituation among the monitored population to enable collars to be deployed without the need for capture. HM17 and HM19 were the same group two years apart, with similar home range, but only three individuals in common, two of which had different statuses in these 2 years (see Table S2).

Individuals were attributed one of six different social statuses based on established protocols at the KMP: dominant females (one per group), dominant males (one per group), other adults (2+ years), yearlings (< 2 years), sub-adults (< 1 year) and juveniles (< 3 months). We based our assessment of individual dominance on long-term data continuously collected at the KMP. Dominance in meerkats is typically unambiguous, and can be determined in the field based on both behavioral traits (dominance assertions, see^37,46^) and physiological traits (enlarged anal glands in males^47^, increased body size in females^48^).

#### Collar design, deployment and duty cycle

**Figure 1:**
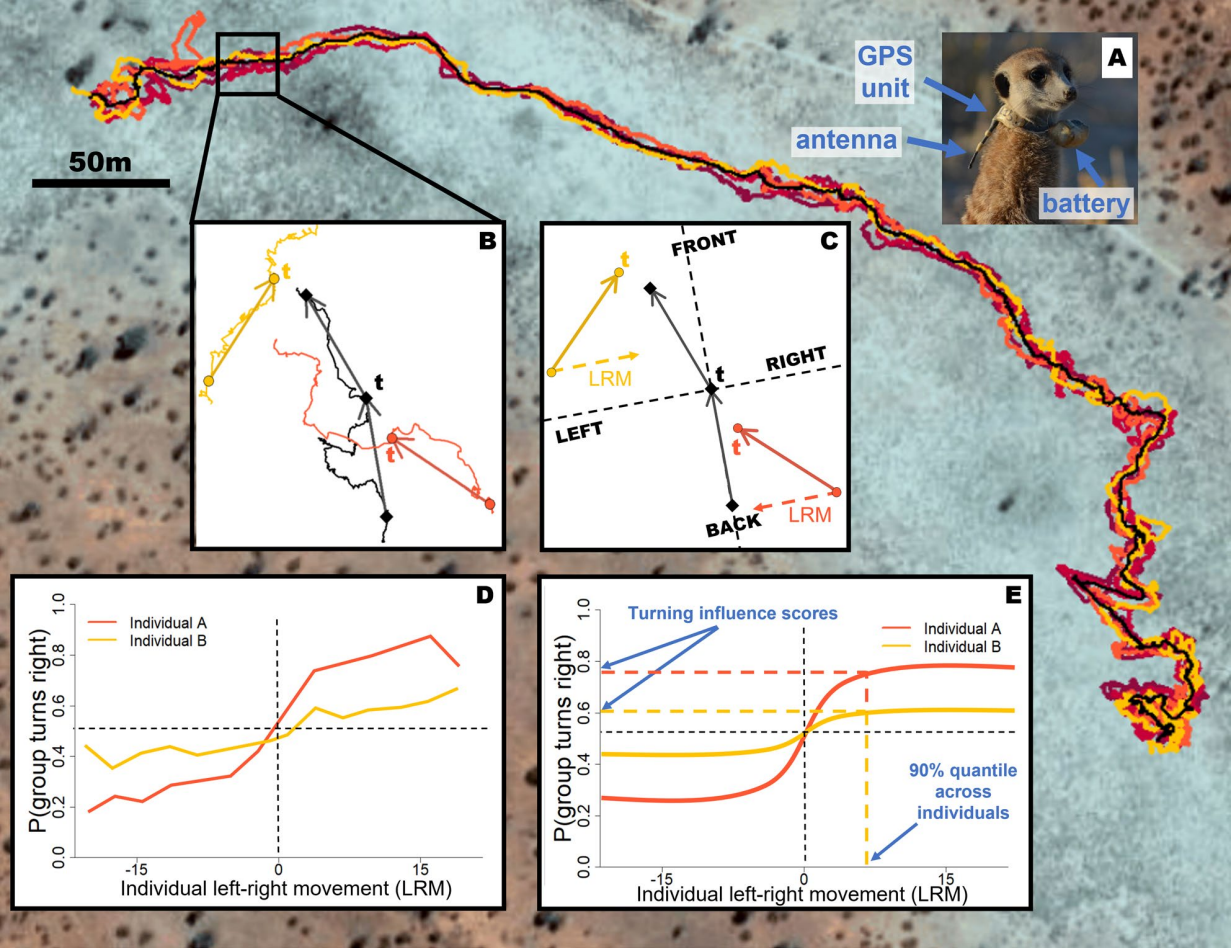
Summary of the data processing pipeline to calculate the turn influence scores of each individual. Background image shows the trajectories of six meerkats from group HM17 recorded over a 3-h time period at 1 Hz using GPS collars. The black line represents the trajectory of the group centroid over the same time-interval, obtained by averaging the coordinates of every individual in the group at each time step. (**A**) Photo of a meerkat wearing a GPS collar. (**B**) Close up of a portion of the trajectory, with only the group centroid and two individuals shown, in yellow and orange, for clarity. At a given time *t*, velocity vectors (solid arrows) are calculated for the past and future movement of the group (black arrows) and individual (colored arrows) using spatial discretization. (**C**) The centroid’s *past* velocity vector is used to define a group reference frame (dashed lines) relative to which the position and movement of individuals are calculated. Based on the centroid’s *future* velocity vector, the group is defined as either turning left or turning right at time t (turning left in the example). An individual’s left–right movement (LRM, dashed arrows) is calculated as the component of its past velocity vector along the left–right axis of the group reference frame. In this example, the orange individual has a positive turn influence at time *t* because it was moving toward the left side of the group before the group turned left. Conversely, the yellow individual has a negative turn influence at time *t* because it was moving toward the right side of the group before the group turned left. (**D**) After aggregating data across all time steps, the probability of the group to turn right as a function of an individual’s left–right speed can be estimated. Exploratory analyses showed that this curve typically shows a sigmoid-like shape: as an individual moves faster toward the right, the probability of the group to turn right in the future increases (and correspondingly for the probability of turning left). However, the extent and steepness of this increase varies for different individuals, which we interpret as differences in *influence*. Here, the orange individual has a higher influence on the rest of the group than the yellow individual (steeper curve). (**E**) We model these influence curves using a modified logistic function, and the 90% quantile of the left–right movement across all individuals of a given group is used to compute a single turn influence score for each individual. The speed influence score is calculated analogously, using instead the probability of the group to speed up as the response variable and the difference between group and individual front-back speed as the predictor variable (not shown, see main text). Note that in the real analyses, the data for a given individual whose influence is being measured is excluded from the computation of the centroid location and movement, to avoid circularity. See Supplemental Materials Sect. 1c for a more detailed description of the method.

We simultaneously recorded the trajectories of all or most individuals in meerkat groups at 1 Hz resolution using custom-built GPS collars (Fig. 1A; Gipsy 5 in 2017 and 2019, Axy-Trek Mini in 2021; Technosmart, Colleverde, Italy). Collars weighed 22–25 g (< 5% of body weight). Juveniles were not recorded due to their small size. GPS loggers recorded for 3 h each day during times when meerkats typically forage within their territory while moving as a group (either in the morning after the group had left the sleeping burrow, or in the afternoon before returning to it), over the course of 5–13 days per group. During recording sessions, an observer noted the times of any group-level disturbances (predator alarms, inter-group encounters, and resting periods) on an all-occurrence basis, and these events were excluded from subsequent analyses. See Supplemental Materials Sect. 1 for details on collaring, group composition, and data pre-processing. All methods were performed in accordance with relevant guidelines and regulations and are reported following the recommendations of the ARRIVE guidelines where relevant to observational field studies.

### Analysis

All analyses were performed in R version 4.0.3^49^, using packages *nlme*^50^, *multcomp*^51^ and *correlation*^52^.

#### Quantifying turn and speed influence

To quantify individual influence from movement data, we define two complementary metrics, designed to serve as proxies for influence over group direction (*turn influence*) and group speed (*speed influence*) separately. For each metric, we evaluate a given individual’s *influence* on the group by measuring the probability that the group’s movement temporally follows that individual’s movement. Each metric also describes how these probabilities change as a function of how extreme an individual’s movement is relative to the group. Briefly, *turn influence* is defined as the probability that the group turns in a given direction (left or right) as a function of the focal individual’s movement along the group’s left–right axis. *Speed influence* is defined as the probability that the group speeds up as a function of the difference between individual and group movement along the front-back axis of movement. For each metric, we fit curves to describe the relationship between each individual’s movement and the group’s subsequent movement, using a modified version of a logistic function. We then used these models to attribute a “turn influence score” and a “speed influence score” to each individual for both metrics. Finally, we fit Linear Mixed Effect Models for each influence score to compare their values among social statuses, using R package *nlme*. For a detailed description of the method, see Supplemental Materials Sect. 1c, and for a visual description see Fig. 1.

An important methodological detail is that our approach uses spatial rather than temporal thresholds for computing individual and group velocity vectors. For example, we define an individual’s future velocity vector as the vector pointing from its current position to its position after it has moved a distance of at least *R* meters, divided by the amount of time taken to get there. This is important because the stop-and-go nature of foraging movement in individual meerkats (and individuals of many other species) makes the temporal scale at which movements occur highly variable. Such a spatial approach also avoids introducing noise in the headings due to small fluctuations in measured GPS positions when groups are relatively stationary^53^. Both of these features make the method broadly applicable to tracking data from many systems, and especially appropriate for terrestrial species which do not show continuous, highly aligned movement. Here we chose *R* = 10 m as the spatial threshold, as this reflects a biologically meaningful spatial scale for the system. To check for robustness, we repeated the analyses with thresholds of 5, 15 and 20 m, and obtained broadly similar results (see Supplemental Materials Sects. 4, 5).

We also defined alternative versions of both metrics based on the spatial location of individuals within the group rather than their movement and compared the outcomes of the two versions (see Supplemental Materials Sects. 2, 3).

#### Quantifying proportion of time spent in the front

To assess whether individuals differ in their propensity to be at the front of the group, we quantified for each individual the distribution of its front-back position relative to the direction of group travel. We also calculated the proportion of time each individual spent in the front half of the group, as a simple metric of ‘frontness’, to allow comparison with our influence scores and to include as an explanatory variable in the models of influence (see below). At time *t*, a given individual was considered in the front half of the group if its front-back position in the group reference frame was positive. To quantify the variation in the propensity to be in the front between individuals and across groups, we computed the proportion of time points an individual was in the front half of the group in time segments of one hour.

#### Assessing the relationships between influence, social status, and time spent at the front of the group

To test if there are consistent differences in influence based on individual social status, we fitted linear mixed effects models (LMMs) predicting influence score as a function of status (dominant female, dominant male, adult, sub-adult, juvenile), for both types of influence. Each individual’s influence score was considered as one data point in the models, and we included group as a random effect to account for non-independence of data within each group. To assess whether social status is linked with propensity to be found at the front of groups, we fitted LMMs predicting frontness as a function of social status. Finally, to test whether frequently being at the front of the group is in itself associated with influence and may mediate the effects of social status on influence, we fitted LMMs incorporating both frontness and social status as predictors. For all LMMs we conducted post-hoc Tukey tests to compare each pair of social statuses (Supplemental Materials Sect. 6).

#### Assessing associations among different types of influence and spatial position

To test for associations among different types of influence, we compared the two forms of influence to one another, as well as each form of influence to the overall proportion of time spent at the front of the group. For each pair of metrics, we computed Spearman multilevel correlations, with group as a random factor.

### Ethics

Field research was conducted under the permission of the ethical committee of Pretoria University, South Africa (permit number: EC031-17). We would like to thank the Northern Cape Department of Environment and Nature Conservation, South Africa for permission to conduct the research (FAUNA 1020/2016).

## Results

### Turn influence and speed influence vary as a function of social status

**Figure 2:**
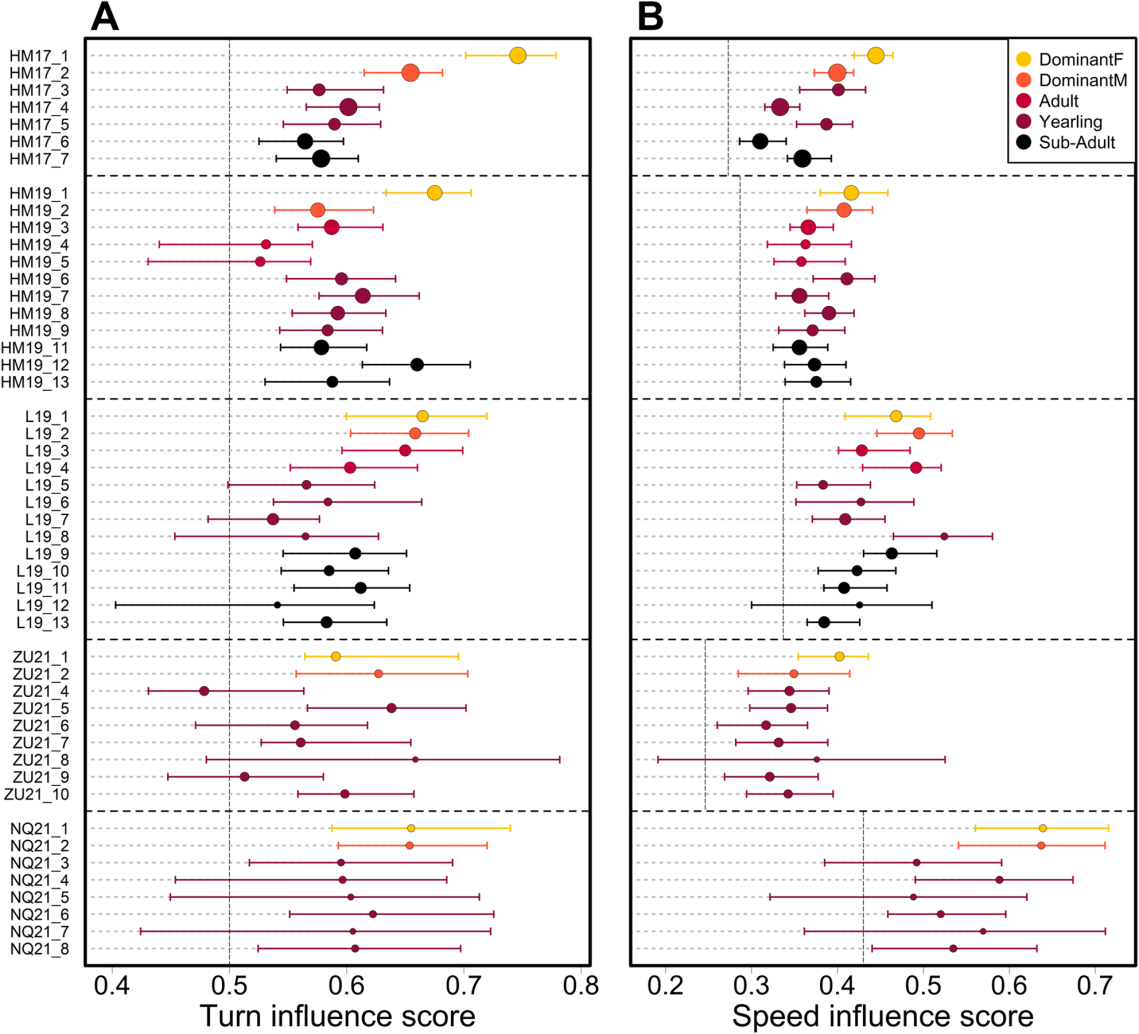
(**A**) Turn influence and (**B**) speed influence scores for each recorded individual (colored dots) in the 5 study groups (vertical axis). Dot color indicates individual status, with dot size proportional to the quantity of data available. Error bars show bootstrapped 90% quantiles (see Supplemental Materials Sect. 1c for details). Dashed vertical lines represent baseline probabilities for the outcome of group decision. This baseline value is 50% for turn influence (equal probability of turning left or right), and is set to the overall probability to speed up for each group for speed influence (because groups tend to accelerate in rapid bursts and then slow down more gradually, the baseline probability of a group speeding up is not 50%—see Supplemental Materials Sect. 1c).

We found that individuals varied substantially in their influence on group direction and speed (Fig. 2, Supplemental Materials Sect. 7), and that social status had a significant effect on both turn (Fig. 2A, F = 5.63; DF = 40; p = 0.001) and speed influence score (Fig. 2B, F = 6.74; DF = 40; p < 0.0001). In particular, dominant females had outsized turn influence (including having the highest score in four out of five groups), and to a lesser extent speed influence. Post-hoc pair-wise Tukey tests (see Tables S4–S7) showed that the turn influence and speed influence scores of dominant females were overall significantly higher than the scores of all subordinate statuses (i.e. non-dominant adults, yearlings and sub-adults). Dominant males’ turn influence scores were not significantly different from scores of the subordinate classes, but their speed influence scores were significantly different from yearlings and sub-adults. Fitted LMM coefficients reveal an overall pattern of dominant females having highest influence, followed by dominant males, then adults/yearlings, and sub-adults—for both turn and speed influence.

### Influence scores are associated with proportion of time spent in the front

**Figure 3:**
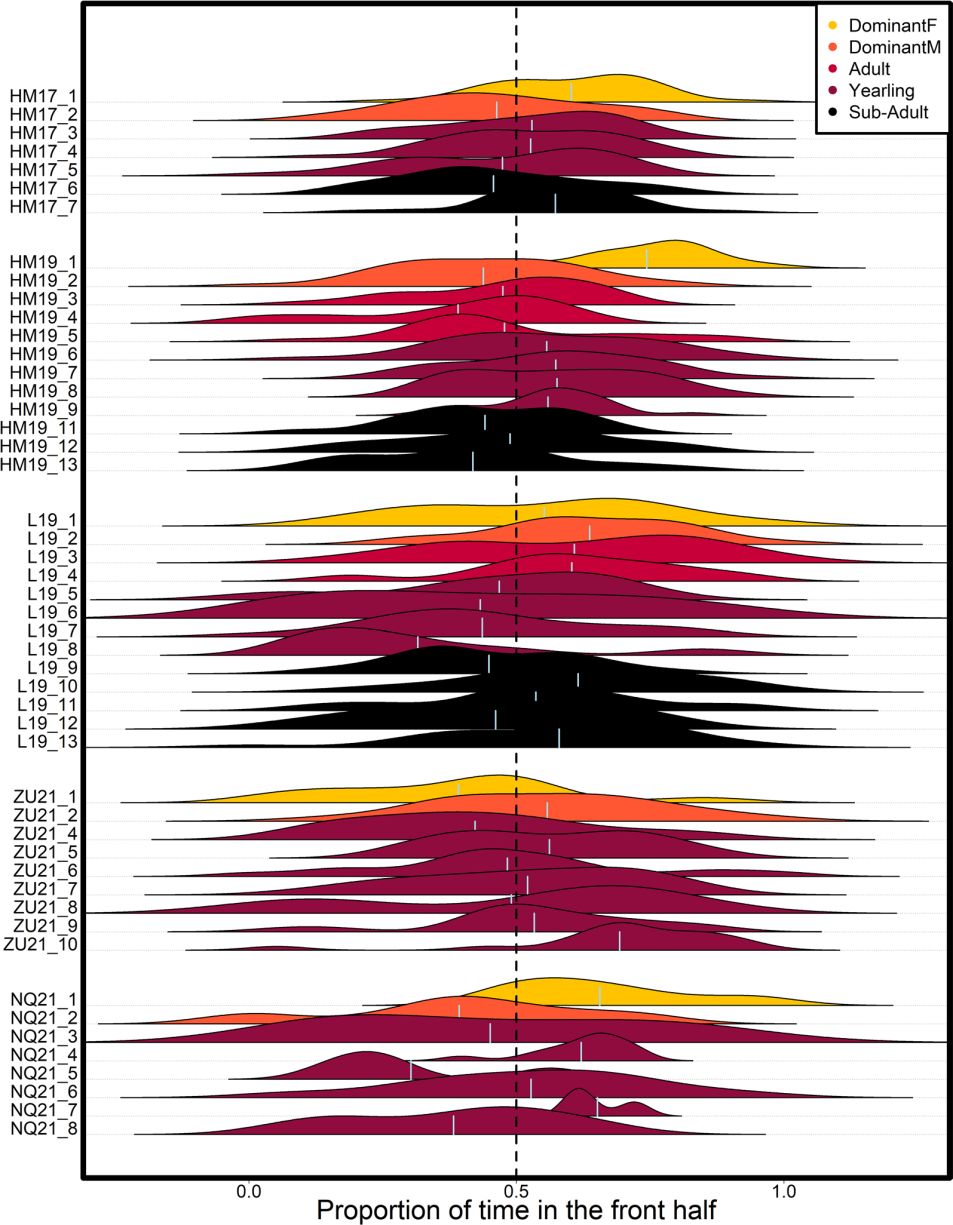
Distribution of the proportion of time spent in the front half of the group over 1-h time periods, for each individual in the 5 study groups (vertical axis). Color indicates individual status, light vertical lines within each distribution indicate the overall mean proportion of time spent in the front half of the group for that individual. Vertical dotted line indicates equal amount of time spent in the front and in the back half of the group.

There was a significant effect of individual social status on the propensity to spend more time in the front of the group, i.e. ‘frontness’ (Fig. 3, F = 6.71; DF = 1036; p-value = 0.0001). The most consistent pattern was for dominant females to spend more time in the front (see Table S8), with the notable exception of group ZU21 (which is also the one group where the dominant female did not have the highest turn influence). In models incorporating both status and frontness, frontness had a significant positive effect on individual turn influence score, with those more often at the front having higher turn influence (Table S9, F = 5.46; DF = 39; p-value = 0.025). However, there was no significant effect of frontness on individual speed influence score (Table S11, F = 0.97; DF = 39; p-value = 0.332). Fitted model coefficients (Table S10) and post hoc pair-wise Tukey tests (Table S12) showed similar results for the effect of status on influence as seen in models not incorporating frontness, suggesting a direct effect of social status on influence that is not entirely mediated by frontness.

**Figure 4:**
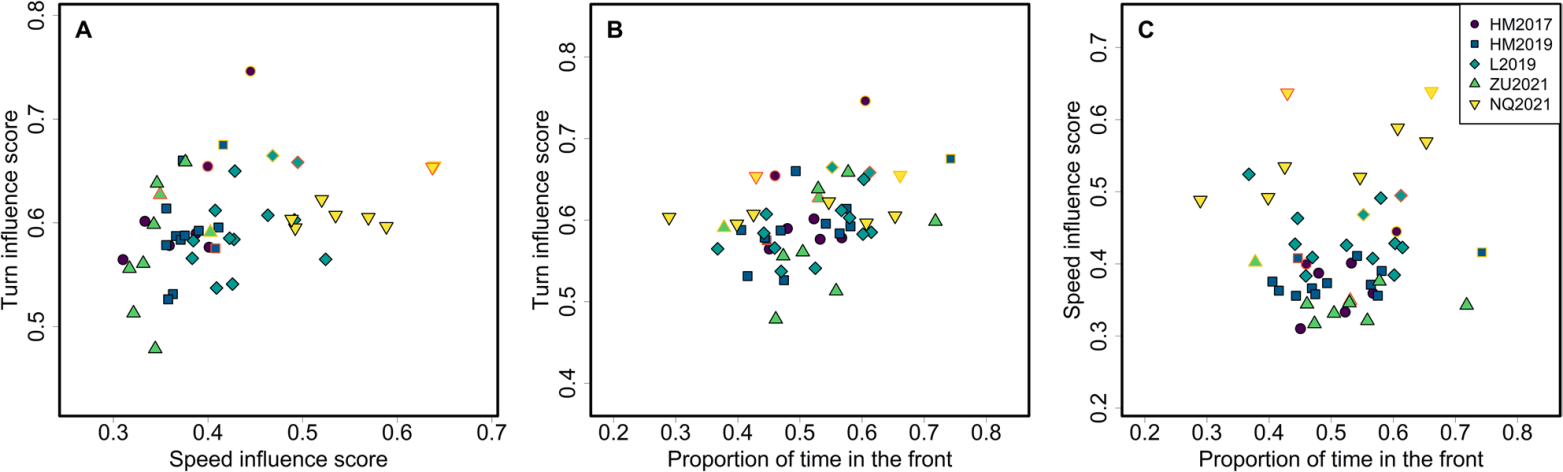
Pairwise associations between turn influence scores, speed influence scores, and proportion of time spent in the front half of the group. Each point represents one individual, with color and shape indicating group membership. Dominant females and dominant males are indicated by yellow and orange borders, respectively. (**A**) Turn influence vs. speed influence. (**B**) Turn influence vs. proportion of time spent in the front. (**C**) Speed influence vs. proportion of time spent in the front.

We found weak positive correlations between individuals’ turn influence scores and their speed influence scores (r = 0.45; p = 0.001; Fig. 4A) and between individuals’ turn influence scores and the overall proportion of time spent in the front half of the group (r = 0.37; p = 0.009; Fig. 4B). There was no significant correlation between individuals’ speed influence scores and the overall proportion of time they spent in the front half of the group, though the estimated direction of this relationship was also positive (r = 0.22; p = 0.120; Fig. 4C).

## Discussion

The nature of group decision-making can be multi-faceted, with individuals exerting influence in one context not necessarily wielding it in others. The method we developed here provides a simple way to separately quantify individual influence over group direction and speed from movement data. Using this method, we reveal that meerkats differ substantially in the amount of influence that their movement exerts on their group, and these differences are linked to social status. Moreover, while the different forms of influence show a weak positive correlation with one another, they also exhibit substantial variation, highlighting the importance of capturing multiple dimensions of influence.

In social mammals, previous work has often found that socially dominant individuals, and particularly dominant females, are the most influential individuals^5,12,44,54^, though exceptions are also found^11,14^. Here, we found dominant females to have significantly higher turn and speed influence than other social statuses, whereas the pattern for dominant males was less consistent. In mammals, the finding that dominant females appear to wield more influence than dominant males is often interpreted in light of the higher energetic requirements of reproduction in females, with leadership potentially providing females with priority of access to higher-quality resources, therefore compensating the costs of pregnancy and/or lactation^55,56^. In meerkats, dominant females have indeed been found more likely to initiate group movement via calling when they are breeding than when they are not^43^. However, in the aforementioned study as well as in ours, dominant females still had in general higher influence than other social statuses, regardless of their reproductive state (see Table S2), so this is unlikely the only explanation. Additionally, meerkats exhibit several cooperative breeding behaviors, including allolactation^57,58^, so even though dominant females are usually the only ones in their group to bear pups, after birth the cost of reproduction is distributed amongst group members. It is noteworthy that in our data the one dominant female that did not have the highest turn influence in her group and also spent more time in the back of the group (group ZU21) had by far the longest tenure at the time of data collection amongst dominant females in our study (104 weeks versus 38 weeks maximum, see Table S2). This suggests that dominant females might exert more influence in the beginning of their tenure, perhaps due to a need to more strongly exert their preferences after attaining dominance, or due to losing influence over time. While the current study cannot yet address these possibilities, the methods developed here provide a foundation for future studies investigating more detailed questions about how group composition and social structure shape the distribution of decision-making across decision types.

Prior studies of meerkat decision-making in other contexts, using different methodologies, have given differing perspectives on the level of decision-sharing. For example, a study of burrow usage in meerkats^42^ showed that groups were more likely to switch burrows when their dominant females had low foraging success, but not necessarily when subordinates did, suggesting dominant females wield outsized influence on the (directional) decision of which burrow to use. In contrast, another study^33^ investigating the timing of return to the communal burrow found that these decisions were more strongly influenced by the foraging success of subordinate group members than by that of dominants. Though both studies also highlighted other factors that played a role in decisions regarding when to return and which burrow to use, taken together they highlight a potential outsized influence of dominant females over directional but not timing decisions. Furthermore, a study of vocalizations associated with changes in group speed found no evidence of outsized influence by dominant individuals^15,41^, again suggesting a lesser role of dominant individuals in influencing timing decisions. Our results are broadly consistent with these findings, as the pattern of dominant female influence was stronger and more consistent in the realm of turn influence than for speed influence. Thus, while our results suggest that both types of decisions are more influenced by dominant individuals, decisions regarding the timing or speed of movement may fall further toward the shared end of the spectrum. This difference could be explained by the fact that it is easier to “compromise” on the (speed) decision of how quickly to move, but not on the (directional) decision of which discrete location to choose, leading to different distributions of consensus costs to group members^21^. Moreover, in meerkats, sub-optimal decisions regarding the direction of movement may prove very costly for group members, as groups could end up in a location with little food or no sleeping burrows, or even in rival territories. Dominant females are typically the oldest natal individuals in the group with the greatest amount of experience navigating the group territory, making their decisions regarding direction likely to be reliable. In contrast, decisions regarding speed may be less dependent on such experience and more dependent on local conditions and the current motivational states (e.g. hunger) of individuals.

The tendency to be in the front of the group is sometimes taken as a proxy for leadership in studies of group movement, but the validity of this assumption is debated^20,59,60^. Here, we found that individuals’ influence over the group direction was linked with their position along the front-back axis of movement. This pattern was particularly striking for the dominant females: the one dominant female that did not have the highest turn influence of her group was also the only one that did not typically occupy a frontmost position. In contrast, we found no evidence that frontmost individuals had higher influence over group speed.

A possible explanation for these divergent results is that it may be easier to influence group direction from the front of the group, whereas speed may be possible to control from other locations. The ability to exert influence from different positions in a group should depend strongly on the range and modality of information transfer. If individuals broadcast vocal signals to the majority of the group, their relative spatial position may be less important in determining their level of influence. Meerkats are known to use a range of vocal signals to maintain cohesion and coordinate movement^15,30,31,61^. Thus individuals should have the potential to convey information, and therefore influence others, from anywhere in the group. For example, meerkats use specific ‘move calls’ to increase group speed, but these calls were not found to be associated with changes in direction^15^. Thus, influencing speed via move calls may be possible to accomplish from anywhere in the group, whereas influencing direction may require an additional (likely visual) cue to indicate which direction is proposed. Future work could expand on the influence framework we have developed here to investigate the role of vocalizations in mediating influence relationships within meerkats and other species.

The nature of influence relationships in groups is also likely to be shaped by environmental factors which were not accounted for in the present work. For instance, individuals’ motivation regarding when and where to move are influenced by the distribution of prey across the territory, and by their past and current foraging success. Similarly, differences in predator encounter probability might change individual trade-offs, hence influencing the movement dynamics of groups. Furthermore, environmental factors such as vegetation and wind can constrain or facilitate the flow of information over different modalities, leading to potential changes in the dynamics of group coordination^62^. Future work incorporating data about environmental parameters, predator and prey distribution, as well as foraging success could give insight into how these factors shape decision-making dynamics in social groups.

Group size and composition are also likely to affect the way influence is distributed among group members. Though our current sample size at the level of groups is too small to address this question, the variability in patterns of influence across groups suggests additional possible drivers of the distribution of influence. For example, the smallest group in our sample (HM19, 7 individuals) was also the one where the dominant female had the strongest turn influence relative to other members, suggesting that dominant individuals may be more able to exert influence in groups with fewer individuals. Age proximity between the dominant female and the eldest subordinate individuals as well as dominance tenure (as discussed above) are also likely to impact the way dominant females can exert influence. Expanding the methods developed here to a larger number of groups of different compositions would enable us to test how the specific configuration of social groups may shift the balance of power over collective decisions.

The method developed here is designed to be broadly applicable to any cohesively moving social group, and could prove useful in comparing how the distribution of influence over group speed and direction varies across species. We expect the sigmoid-like shape of influence curves to be a general feature across systems, as long as the basic assumption holds that more extreme movements or spatial displacements of an individual along a given dimension result in a greater propensity of its group to turn or speed up along that dimension. However, we note that this assumption still requires empirical validation within a given species and dataset. If this assumption does not hold, a possible simplification would be to compute the probability of the group turning right or speeding up when an individual moves right or moves faster than the group respectively, and use this value as the influence score rather than fitting influence curves. Furthermore, though the method as currently developed is only applicable to cohesively moving groups, it may be possible to adapt it to less cohesive or even fission–fusion species with some modifications. In particular, the use of the mean position (group centroid) to represent the trajectory of the group may be sensitive to individuals that move far away from the group and would also become unrepresentative in cases of group splits (which are rare in meerkats). A possible adaptation for fission–fusion species could involve first identifying subgroups (e.g. using spatial clustering) and then using the method to analyze influence relationships within these subgroups, or even among different subgroups.

Our approach, by design, captures influence aggregated over time. It should therefore also be compared with complementary approaches—for instance those analyzing specific events such as sharp changes in direction, increases in speed during movement, or emission of specific calls—to gain a more complete picture of the distribution and variability of influence in social groups.

## Conclusion

The work presented here aims at disentangling influence over group speed and direction when assessing patterns of influence in moving animal groups. In meerkats, we find that these two components of influence are linked and associated with dominance, but also show substantial variation. Assessing whether other species show consistent or divergent patterns of influence over direction and speed offers the potential to test theoretical predictions regarding the level of decision-sharing in these different domains^21^. The results presented here highlight the complexity of the concept of influence, and demonstrate the need to study it from different perspectives across multiple groups to gain a more complete understanding of collective decision-making in animal societies.

## Supplementary Information

Supplementary Information.

## Data Availability

Raw data and scripts are available on GitHub (https://github.com/BaptisteAverly/Meerkat_TurnSpeed_Influence.git) and Zenodo 10.5281/zenodo.6913199.
