# Supplementary material for: Disentangling influence over group speed and direction reveals multiple patterns of influence in moving meerkat groups
Source: Sci Rep. 2022 Aug 16;12:13844. doi: 10.1038/s41598-022-17259-z (PMC9381760; doi:10.1038/s41598-022-17259-z)
Supplement: Supplementary file 1 — Supplementary Information. [file 41598_2022_17259_MOESM1_ESM.docx]

Supplemental Materials

**1 - Methodological details**

***a - Collaring process***

In addition to GPS units, collars also included a small audio recorder (Edic-mini Tiny+ A77; TS-Market, Russia). Axy-Trek units (used in 2021) also collected accelerometry data. However, only GPS data was used in the present study. The collar closing mechanism consisted of 2 magnets (1 x 5 x 5 mm) glued to 3-D printed plastic clasps at each end of the leather strap, designed to be able to close easily but to require human intervention to open.

We deployed most collars in the morning hours, when meerkats were at their communal sleeping burrow to warm up in the sun or groom each other prior to foraging. Some collars were also deployed in the afternoon hours when meerkats stopped foraging and rested in the heat, or while meerkats were grooming one another at the sleeping burrow before descending into the burrow for the night. A few collars were also opportunistically deployed in conjunction with scheduled captures of individuals for other purposes at the long-term study.

A collar of the appropriate size was selected based on prior neck measurements of the target individual. We put it on after carefully approaching and grooming the target meerkat and only if the animal remained stationary and showed no sign of discomfort or attempted escape. Some individuals were collared while drinking water which was presented for distraction (Figure S1). No more than two attempts of collaring were done per day per individual to prevent dishabituation of the meerkats to human observers. After successful collaring, individuals were observed for at least ten minutes and the collar was removed if they exhibited any kind of unusual behavior for more than a few minutes (e.g. scratching at the collar, trying to remove it). This happened in very few cases, always on the smallest individuals. In these few cases we removed the collars and, in some instances, re-deployed different collars on a subsequent day (if collar fit was determined to be the issue). At the end of data collection, collars were taken off much in the same way as they were put on or by cutting the leather straps using mini diagonal cutters, though sometimes as well during foraging since removal could be done much more quickly and easily than deployment. See Tables S1, S2 and S3 below for information on group composition, deployment timing and individual characteristics.

Across all groups, a total of 14 non-juvenile individuals that could not be collared (this included one dominant per group already carrying a VHF collar for tracking) or for which collar GPS had failed mid-session were instead continuously recorded by a human observer. A GPS tag equivalent to those deployed in collars was strapped to a directional microphone on the end of a telescopic pole and kept within 1 meter of the foraging meerkat for the 3-hour duration of each session. At the same time, the observer vocally described the focal meerkat’s behavior using a handheld microphone, including noting occasional moments when the meerkat went out of range of the pole (these portions were then removed from the recorded trajectories). The numbers of meerkats which could be focal-followed in this way depended on the number of observers available (never more than 3), therefore not every non-juvenile could be recorded on every day, especially as GPS tags started failing due to low battery towards the end of each deployment round. We processed the data of focal followed individuals in the same way as data from meerkats wearing collars.


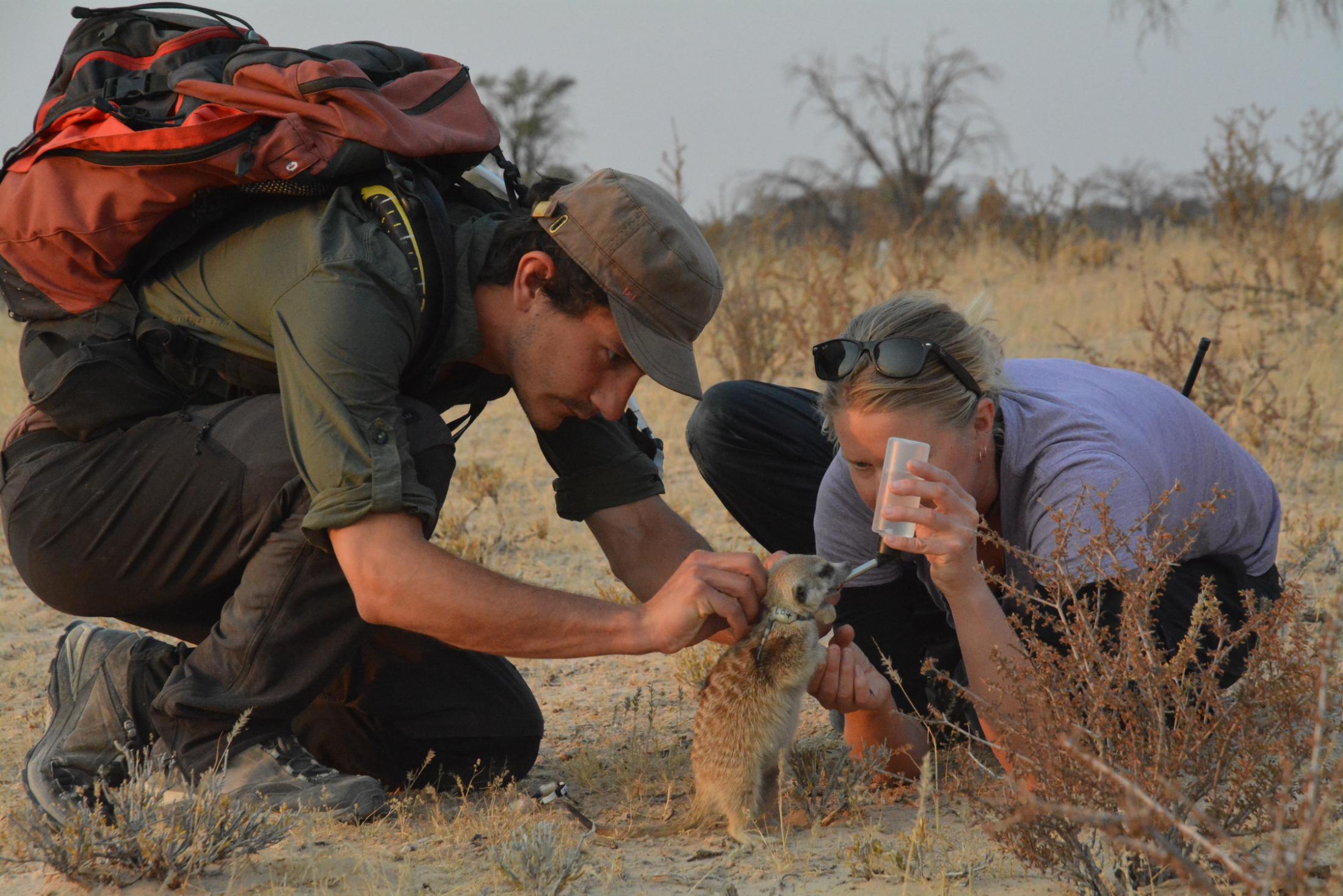
Figure S1. Image showing the collar deployment process.

***b - GPS data pre-processing***

GPS coordinates were first converted from WGS84 to UTM S34 to allow for easier spatial analyses. To increase GPS reliability and reduce sampling biases, we performed minimal pre-processing of GPS data before subsequent analysis. Specifically, when GPS signals were not recorded continuously (for instance if signal was lost after a meerkat went below ground, e.g. entering a bolt-hole) we discarded all GPS fixes taken 30s before signal loss and 30s after signal return, as these positions tended to be unreliable. We also removed fixes with fewer than five satellites detected.  Finally, in six instances we removed data suggesting biologically unrealistic speeds (>10 m displacement between two fixes one second apart) as these likely represented GPS errors.

In some instances, single individuals were away from the rest of the group during recording times, either at the communal burrow babysitting pups, out travelling on their own (“roving” behavior exhibited by adult males before dispersing from their natal territory), and in one case a female being evicted from the group for a few days by the pregnant dominant female. In such instances the GPS trajectory of that given individual was discarded but the analyses were performed normally on the rest of the group.

Due to GPS tag battery failure and unsuccessful collaring attempts, we could not record every adult group member throughout the whole deployment (Table S2). We excluded time points when fewer than two-thirds of the non-juveniles present that day were recorded, to reduce the impacts of “invisible” (untracked) individuals. We also removed periods where the group interrupted their normal movement behavior such as predator alarm responses, rare instances of resting periods due to the heat, one instance of an encounter with another meerkat group, and one day when three adult males (including the dominant) were not present with the group. After the removal of these data, we were left with a minimum of 9.5 hours (NQ21) and a maximum of 37.5 hours (HM17) of usable data per group (Table S1).

***c – Detailed method for computing influence scores***

To compute the influence metrics for a given focal individual, we first computed the track of the group centroid by averaging the positions of all individuals recorded (other than the focal individual) at each time point (Figure 1B). For every time point *t*, we then calculated the *future* and *past* velocity vectors of the group centroid (Figure 1C). The *future velocity vector* was defined as the vector pointing from the position at time *t* (henceforth ‘current position’) to the next recorded position that was at least 10 meters away (henceforth ‘future position’). The *past velocity vector* was defined as the vector pointing to the current position from the most recent position that was at least 10 meters away (henceforth ‘past position’).

For each time point, we defined a coordinate system with the group’s current position at the origin and its past direction of motion pointing along the y-axis (henceforth *group reference frame*, Figure 1D). We then calculated the past velocity vector of the focal individual at each time point, defined in the same way as for the group past velocity vector, and projected it into the group frame of reference to describe the individual’s movement relative to the group. From this, we computed two variables corresponding to the two metrics of influence: the y component of the individual’s past velocity vector (i.e. left-right movement, corresponding to **turn influence**), and the difference between the x component of the centroid’s past velocity vector and the x component of the individual’s past velocity vector (i.e. front-back movement, corresponding to **speed influence**).

Exploratory analyses showed that the probability of a group turning right as a function of a given individual’s prior movement towards the right increased sharply before plateauing, and conversely for probability to turn left, resulting in a sigmoid-like curve (Figure 1D). A similar shape was observed for speed influence (Section 7 below). For each influence type, we therefore modelled the probability of a binary group response (turn left / right, speed up / slow down) as a function of a continuous individual predictor (movement relative to the group reference frame). Specifically, turn influence is the probability that the group turns right in the future as a function of an individual’s past speed along the group’s left-right axis in the past. Speed influence is the probability that the group speeds up in the future as a function of the difference in speed between an individual and the group along the group’s front-back axis in the past.

To model these probabilities, we fit a modified version of the logistic function to both types of influence, for each individual (equation 1):

$f\left( x \right)= [\alpha\frac{1}{1+e^{-\beta x}}] + [\gamma\left( 1-\alpha\right)]$ (1)

Here, *x* represents the individual behavior (left-right or front-back movement) and *f(x)* represents the probability of the group turning right or speeding up. α and β are variable parameters which were fit for each individual separately, while γ is a fixed parameter which we set as described below. This modified sigmoidal shape was chosen due to its empirical correspondence with the data, as well as the interpretability of its parameters. In particular, α can be interpreted as the probability that the group is influenced by the focal individual at a given time point, and in practice controls the height of the curve. β can be interpreted as the logistic growth rate (steepness) of the curve and hence the strength of influence relative to how much an individual moves. γ is the baseline probability of the group either turning right or speeding up. For turning influence, γ was set to 0.5 (assuming an overall equal probability to turn left or right), whereas for speed influence, γ was fixed to the aggregate probability of a given group to speed up across all the data. Because groups tend to accelerate in rapid bursts but decelerate over longer time periods, the overall probability of a group speeding up is lower than the probability of the group slowing down, hence the value of γ ranged from 0.24 to 0.37.

We fit the values of α and β for each influence metric using maximum likelihood estimation, enabling us to define, for each individual, two curves representing its turn and speed influence (the model fits for each individual are shown in Section 7 below). For ease of interpretation and subsequent modeling, we also defined an aggregate “influence score” as the value of the individual’s fitted curve at the 90% quantile of either continuous predictor variables across all individuals of a given group (Figure 1F). This influence score therefore corresponds to the probability that the group is positively influenced by the focal individual for a fixed amount of movement (either left/right or front/back) relative to the group centroid. We used bootstrapping to calculate confidence intervals on these influence scores, first dividing the data for each individual into N chunks of four minutes (median duration for which the data autocorrelation had dropped close to zero). We then randomly sampled N chunks with replacement, and recomputed the influence score. We re-sampled the data 1000 times for each individual and used the 5% and 95% quantiles of the influence score distributions as the lower and upper bound of the confidence intervals.

***d - Summary tables***

Table S1. Summary of group composition and data collection. df = dominant female ; dm = dominant male ; ye = yearlings ; sub = sub-adult ; juv = juvenile. ‘*Total time used*’ indicates the amount of time steps that were used in the analyses, once irrelevant data were removed (see main text for details).

| Group name | Group size | Group composition  (recorded / present) | Recording  Period | # recording days  used | Recording times | Total time used (hours) |
| --- | --- | --- | --- | --- | --- | --- |
| HM17 | 7 | 1/1 df ; 1/1 dm ;  3/3 ye ; 2/2 sub | 06 Aug –  08 Sep 2017 | 13  (3 deployments) | Days 1-8: 07:00 to 10:00 UTC  Days 9-13: 06:00 to 09:00 UTC | 37.2 |
| HM19 | 18 | 1/1 df ; 1/1 dm ;  3/3 ad ; 4/4 ye ;  3/4 sub ; 0/5 juv | 23 Jun –  19 July 2019 | 12  (2 deployments) | Days 1-5: 08:00 to 11:00 UTC  Days 6-12: 12:00 to 15:00 UTC | 31.4 |
| L19 | 19 | 1/1 df ; 1/1 dm ;  2/2 ad ; 4/4 ye ;  5/5 sub ; 0/6 juv | 05 Aug –  12 Aug 2019 | 8  (1 deployment) | Days 1-8: 08:00 to 11:00 UTC | 18.5 |
| ZU21 | 13 | 1/1 df ; 1/1 dm ;  0/1 ad ; 6/7 ye ;  0/3 juv | 16 May –  24 May 2021 | 8  (1 deployment) | Days 1-8: 08:00 to 11:00 UTC | 15.3 |
| NQ21 | 11 | 1/1 df ; 1/1 dm ;  6/6 ye ; 0/3 juv | 11 Aug –  17 Aug 2021 | 5  (1 deployment) | Days 1-5: 07:00 to 10:00 UTC | 8.8 |

Table S2. Information on all individuals comprising the five recorded groups, with each line representing one individual. Three individuals appear twice as they were present both in HM17 and HM19. (P) next to an individual’s name indicates that this individual was pregnant for at least part of the data collection. DOB = Date Of Birth. ‘*Tenure*’ indicates the dates at which dominance was established (only for dominant individuals). ‘*Natal’* indicates whether or not the individual was born in the group it was recorded in. ‘*Recording type’* indicates how movement data was collected: *collar* = using gps tags mounted on a collar ; *focal* = following the individual with a gps unit strapped to a pole ; *none* = this individual could not be recorded. *‘# record days’* indicates the number of days this individual was recorded out of all the days used in the analysis. *‘# absent days*’ indicates the number of days this individual was absent from the group, out of all the days used in the analysis, with the reason in parentheses: *BS* = baby-sitting at the communal burrow ; *EV* = evicted from the group ; *ROV* = roving. A given individual was thus recorded on every used day if the numbers in the last two columns add up to the number of recording days used for its group as shown in Table S1.

| **Group** | **Name** | **Code** | **DOB** | **Sex** | **Status** | **Tenure** | **Natal** | **Recording type** | **# record days** | **# absent days** |
| --- | --- | --- | --- | --- | --- | --- | --- | --- | --- | --- |
| HM17 | AJB (P) | HM17_1 | 21.08.2013 | F | DominantF | 10.11.2016 | N | Focal | 13 | 0 |
| HM17 | PET | HM17_2 | 21.12.2011 | M | DominantM | 10.11.2016 | N | Collar | 13 | 0 |
| HM17 | FLINT | HM17_3 | 27.10.2016 | F | Yearling | NA | Y | Collar | 7 | 3 (BS) |
| HM17 | RIPTIDE | HM17_4 | 27.10.2016 | M | Yearling | NA | Y | Collar | 13 | 0 |
| HM17 | REEPICHEEP | HM17_5 | 27.10.2016 | M | Yearling | NA | Y | Collar + focal | 1 + 7 | 1 (BS) |
| HM17 | COSMO | HM17_6 | 28.12.2016 | M | Sub-Adult | NA | Y | Collar | 11 | 1 (BS) |
| HM17 | DANTE | HM17_7 | 28.12.2016 | M | Sub-Adult | NA | Y | Collar | 13 | 1 (BS) |
| HM19 | FLINT (P) | HM19_1 | 27.10.2016 | F | DominantF | 10.04.2019 | Y | Collar + focal | 7 + 5 | 0 |
| HM19 | PET | HM19_2 | 21.12.2011 | M | DominantM | 10.11.2016 | N | Focal | 12 | 0 |
| HM19 | DANTE | HM19_3 | 28.12.2016 | M | Adult | NA | Y | Collar | 12 | 0 |
| HM19 | GUACAMOLE | HM19_4 | 01.09.2017 | M | Adult | NA | Y | Collar + focal | 4 + 2 | 0 |
| HM19 | DOUBLE FLUFF | HM19_5 | 01.09.2017 | F | Adult | NA | Y | Focal | 6 | 6 (EV+BS) |
| HM19 | TWIRL | HM19_6 | 15.05.2018 | M | Yearling | NA | Y | Collar | 9 | 3 (BS) |
| HM19 | MOZARELLA | HM19_7 | 15.05.2018 | F | Yearling | NA | Y | Collar | 12 | 0 |
| HM19 | OSCAR JUNIOR | HM19_8 | 15.05.2018 | M | Yearling | NA | Y | Collar | 10 | 2 (BS) |
| HM19 | PINGU | HM19_9 | 15.05.2018 | M | Yearling | NA | Y | Collar | 8 | 0 |
| HM19 | MUNCHKIN | HM19_10 | 26.09.2018 | F | Sub-Adult | NA | Y | None | 0 | 2 (BS) |
| HM19 | SHAMROCK | HM19_11 | 26.09.2018 | M | Sub-Adult | NA | Y | Collar | 12 | 0 |
| HM19 | ACE | HM19_12 | 26.09.2018 | F | Sub-Adult | NA | Y | Collar + focal | 8 + 1 | 0 |
| HM19 | SHANDY | HM19_13 | 26.09.2018 | M | Sub-Adult | NA | Y | Collar | 7 | 0 |
| HM19 | DARJEELING | HM19_14 | 08.02.2019 | F | Juvenile | NA | Y | None | 0 | 0 |
| HM19 | SENCHA | HM19_15 | 08.02.2019 | F | Juvenile | NA | Y | None | 0 | 0 |
| HM19 | KAJESS | HM19_16 | 08.02.2019 | F | Juvenile | NA | Y | None | 0 | 0 |
| HM19 | BELPHEGOR | HM19_17 | 08.02.2019 | M | Juvenile | NA | Y | None | 0 | 0 |
| HM19 | MUGI | HM19_18 | 08.02.2019 | M | Juvenile | NA | Y | None | 0 | 0 |
| L19 | SIGMA (P) | L19_1 | 06.11.2016 | F | DominantF | 26.05.2019 | Y | Collar | 8 | 0 |
| L19 | POLON | L19_2 | 25.04.2016 | M | DominantM | 26.01.2017 | N | Focal | 8 | 0 |
| L19 | FINNICK | L19_3 | 23.04.2017 | M | Adult | NA | Y | Collar | 8 | 0 |
| L19 | TONKS | L19_4 | 23.04.2017 | F | Adult | NA | Y | Collar | 8 | 0 |
| L19 | THURSDAY | L19_5 | 23.02.2018 | M | Yearling | NA | Y | Collar | 4 | 0 |
| L19 | ANNIE | L19_6 | 23.02.2018 | F | Yearling | NA | Y | Focal | 3 | 0 |
| L19 | WEASEL | L19_7 | 23.02.2018 | F | Yearling | NA | Y | Collar | 8 | 0 |
| L19 | FLEDERMAUS | L19_8 | 23.02.2018 | M | Yearling | NA | Y | Focal | 3 | 1 (ROV) |
| L19 | HAOPIA | L19_9 | 18.07.2018 | F | Sub-Adult | NA | Y | Collar | 8 | 0 |
| L19 | LUTHER | L19_10 | 18.07.2018 | M | Sub-Adult | NA | Y | Collar | 6 | 0 |
| L19 | LENNON | L19_11 | 18.07.2018 | F | Sub-Adult | NA | Y | Collar | 8 | 0 |
| L19 | ISRAEL | L19_12 | 18.07.2018 | M | Sub-Adult | NA | Y | Focal | 2 | 0 |
| L19 | NELSON | L19_13 | 18.07.2018 | M | Sub-Adult | NA | Y | Collar | 8 | 0 |
| L19 | SQUELCH | L19_14 | 13.03.2019 | F | Juvenile | NA | Y | None | 0 | 0 |
| L19 | SQUEAL | L19_15 | 13.03.2019 | M | Juvenile | NA | Y | None | 0 | 0 |
| L19 | SIZZLE | L19_16 | 13.03.2019 | M | Juvenile | NA | Y | None | 0 | 0 |
| L19 | SLOP | L19_17 | 13.03.2019 | M | Juvenile | NA | Y | None | 0 | 0 |
| L19 | SPLAT | L19_18 | 13.03.2019 | M | Juvenile | NA | Y | None | 0 | 0 |
| L19 | SAUSAGE | L19_19 | 13.03.2019 | F | Juvenile | NA | Y | None | 0 | 0 |
| ZU21 | SPRUDDEL | ZU21_1 | 10.03.2017 | F | DominantF | 19.05.2019 | Y | Focal | 8 | 0 |
| ZU21 | SCUZI | ZU21_2 | 02.05.2018 | M | DominantM | 01.01.2020 | N | Focal | 6 | 0 |
| ZU21 | JAY-A-ROD | ZU21_3 | 02.05.2018 | M | Adult | NA | N | None | 0 | 0 |
| ZU21 | SALAZAR | ZU21_4 | 09.03.2020 | F | Yearling | NA | Y | Collar | 8 | 0 |
| ZU21 | HELGA | ZU21_5 | 09.03.2020 | F | Yearling | NA | Y | Collar | 8 | 0 |
| ZU21 | GODRIC | ZU21_6 | 09.03.2020 | M | Yearling | NA | Y | Collar | 7 | 0 |
| ZU21 | ROWENA | ZU21_7 | 09.03.2020 | F | Yearling | NA | Y | Collar | 8 | 0 |
| ZU21 | BEAR | ZU21_8 | 27.03.2020 | M | Yearling | NA | Y | Collar | 2 | 0 |
| ZU21 | MION | ZU21_9 | 27.03.2020 | M | Yearling | NA | Y | Collar | 7 | 0 |
| ZU21 | ZUMA | ZU21_10 | 27.03.2020 | M | Yearling | NA | Y | Collar | 6 | 0 |
| ZU21 | SPOEKIES | ZU21_11 | 05.01.2021 | F | Juvenile | NA | Y | None | 0 | 0 |
| ZU21 | WILLOW | ZU21_12 | 05.01.2021 | M | Juvenile | NA | Y | None | 0 | 0 |
| ZU21 | TAZARA | ZU21_13 | 05.01.2021 | M | Juvenile | NA | Y | None | 0 | 0 |
| NQ21 | MANZA | NQ21_1 | 11.11.2019 | F | DominantF | 08.07.2021 | Y | Collar | 5 | 0 |
| NQ21 | UMFANA | NQ21_2 | 07.04.2018 | M | DominantM | 01.07.2021 | N | Focal | 5 | 0 |
| NQ21 | ZUKO | NQ21_3 | 11.11.2019 | M | Yearling | NA | Y | Collar | 4 | 0 |
| NQ21 | MONONOKE | NQ21_4 | 11.11.2019 | F | Yearling | NA | Y | Collar | 5 | 0 |
| NQ21 | HICCUP | NQ21_5 | 01.02.2020 | F | Yearling | NA | Y | Focal | 2 | 0 |
| NQ21 | BARNEY | NQ21_6 | 22.04.2020 | M | Yearling | NA | Y | Collar | 5 | 0 |
| NQ21 | COOPER | NQ21_7 | 22.04.2020 | M | Yearling | NA | Y | Focal | 1 | 0 |
| NQ21 | MEGARA | NQ21_8 | 22.04.2020 | F | Yearling | NA | Y | Collar | 5 | 0 |
| NQ21 | MIA | NQ21_9 | 07.03.2021 | F | Juvenile | NA | Y | None | 0 | 0 |
| NQ21 | FREDI | NQ21_10 | 07.03.2021 | M | Juvenile | NA | Y | None | 0 | 0 |
| NQ21 | HANSPETER | NQ21_11 | 07.03.2021 | M | Juvenile | NA | Y | None | 0 | 0 |


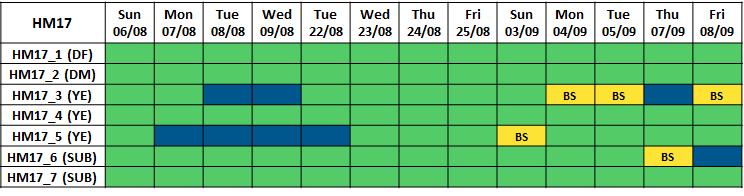
Tables S3. Summary of data collection effort for all individuals (rows) on each recording day (columns). A green cell indicates that the individual was present in the group and recorded on that particular date, a dark blue cell indicates that it was present in the group but not recorded, a yellow cell indicates that it was absent from the group, with reason indicated as in Table S2.

**
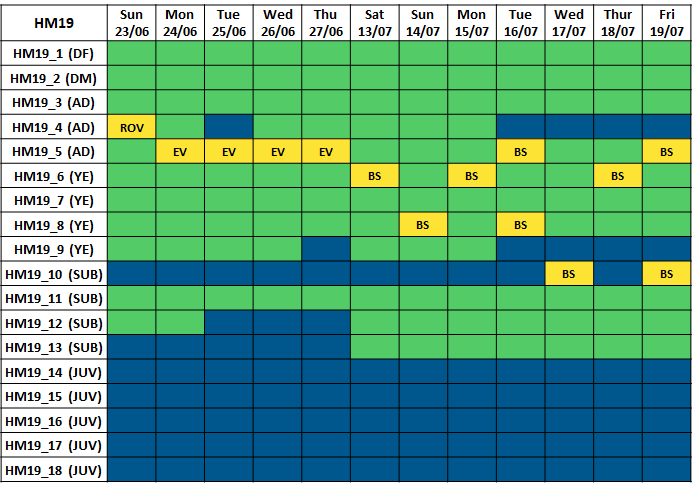
**

**
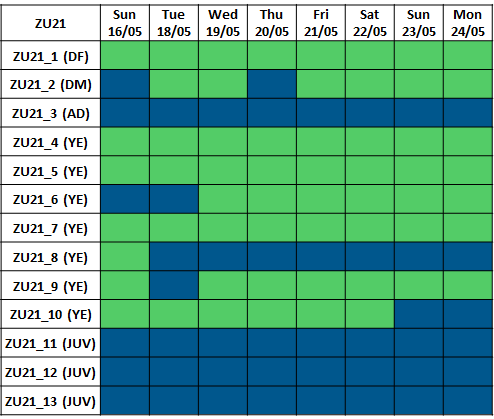

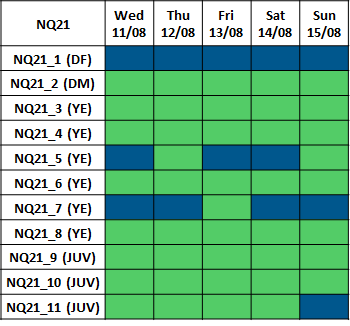

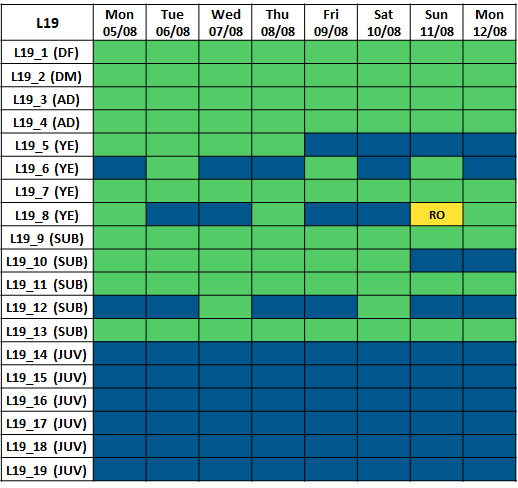
**

**2 - Alternative influence scores based on individual positions**

Depending on the movement characteristics of the social species under study, individual cues that exert influence on the rest of the group’s movement can vary. In addition to the two influence metrics based on individual relative movement (i.e. the group follows their speed and/or direction of motion, see main text), we also considered the possibility that individuals might exert influence via their relative spatial positioning (i.e. the group moves toward their position in space).

**Positional turn influence** is defined as the probability that the group turns in a given direction (right or left) as a function of an individual’s position to the left or right of the group center. Similarly, **positional speed influence** is defined as the probability that the group speeds up as a function of the front-back position of an individual.

Similar to the movement-based turn influence presented in the main text, the binary response variable for positional turn influence is the probability of the group to turn right, but the continuous predictor variable is the left-right *position*, defined as the y-value of the individual’s past position in the group reference frame (see Figure S2 below) rather than the left-right movement speed. Similarly, the binary response variable for positional speed influence is the probability of the group to speed up and the continuous predictor variable is the front-back position, defined as the x-value of the individual’s past position in the group reference frame. See methods in the main text and Supplemental Materials Section 1c for more details about data processing.


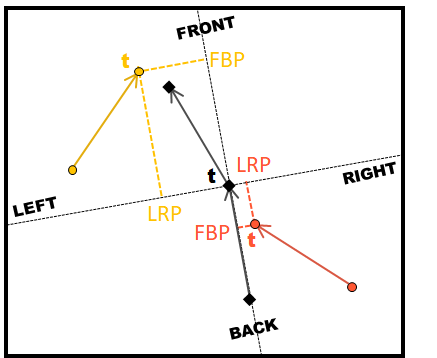
Figure S2. Calculation of individual metrics used to get the positional turn and positional speed influence scores. Left-right position (LRP) is the y-value of the individual’s current position in the group reference frame (doted line) and front-back position (FBP) is the x-value of the individual’s current position in the group reference frame. Colored solid arrows represent the past velocity vectors of the two individuals, black solid arrows represent the past and future velocity vectors of the group centroid. See Figure 2 in the main text for more details on discretization process and calculation of the group reference frame.

The probability of the group to turn right as a function of individual left-right position (positional turn influence) and the probability of the group to speed up as a function of individual front-back position (positional speed influence) were then modelled using a modified logistic function (see equation 1 in the main text), and influence scores were derived from it in the same manner as described for the metrics used in the main text (see figures 2E and 2F).

**B.**

**A.**

Figure S3. Predicted positional influence scores for each recorded individual (colored dots) in the 5 study groups (vertical axis). Dot color indicates individual status as shown in the legend, dot size is proportional to the quantity of data available. Dotted vertical lines represent baseline probabilities for the outcome of group decision (50% percent chance of turning left or right for turn influence and overall probability to speed up for each group for speed influence). (A) Positional turn influence score represents the probability that the group turns toward the side (left or right) where the individual was located. (B) Positional speed influence score represents the probability that the group speeds up after that individual was in the front half of the group.

**3 - Logistic modelling with two predictor variables**

To compare the strength of the two possible effects (position and movement), we conducted an additional analysis in which we modelled the overall probability of groups to turn right as a function of both their members’ past left-right positions and past left-right movements relative to the group’s past heading. Similarly, we modelled the probability of groups to speed up as a function of both their members’ past front-back positions and past front-back movements relative to the group’s heading.

We first computed all four individual variables (left-right movement and position and front-back movement and position) as detailed in the main text and in section 3 above. We then fit two models, one for turn influence and one for speed influence, using data from all individuals across our five groups. The function we used for the models is a modified version of equation 1 presented in the main text, allowing for the use of two predictor variables (equation 2 below):

$f\left( x \right)= [\alpha\frac{1}{1+e^{-\left( \beta_{1}x_{1}+\beta_{2}x_{2} \right)}}] + \left[ \gamma\left( 1-\alpha\right) \right]$ (2)

Here, *f(x)* represents the probability of the group either turning right or speeding up, depending on the type of influence, as in equation 1. *x_1_* represents individual position (left-right or front-back) and *x_2_* represents individual movement (left-right or front-back) as described in the main text. *α*, *β_1_* and *β_2_* were fit for all individuals combined, while *γ* was fixed to either 0.5 for turning influence (assuming an overall equal probability to turn left or right), or to the aggregate probability of all groups to speed up across all the data for speeding influence.

These analyses revealed that in general, the future direction and speed of the group was more strongly associated with individuals’ relative speed than with their relative spatial positions in the past (Figure S4). This result also held at the group and individual level. This result informed our decision to focus our main analyses on movement rather than position as a driver of influence.

Figure S4. Predicted group outcome as a function of individual position and movement, modeled using data across all groups. (A) Predicted probability of a group to turn right as a function of individual left-right position (y-axis) and individual left-right speed (x-axis). (B) Predicted probability of a group to speed up as a function of individual front-back position (y-axis) and front-back movement (x-axis). Axis limits extend to the 0.01-0.99% quantiles of each variable to show them on comparable scales.


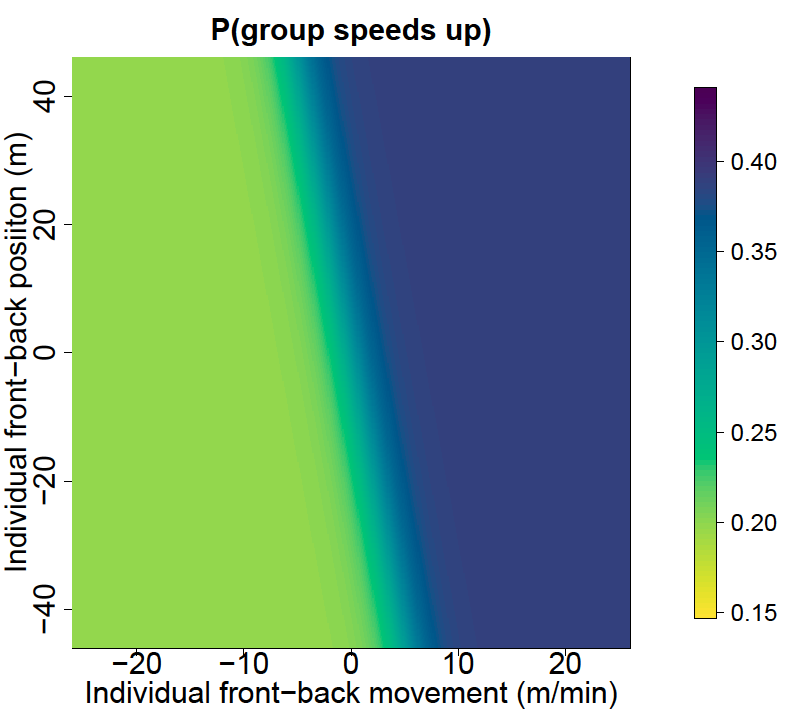

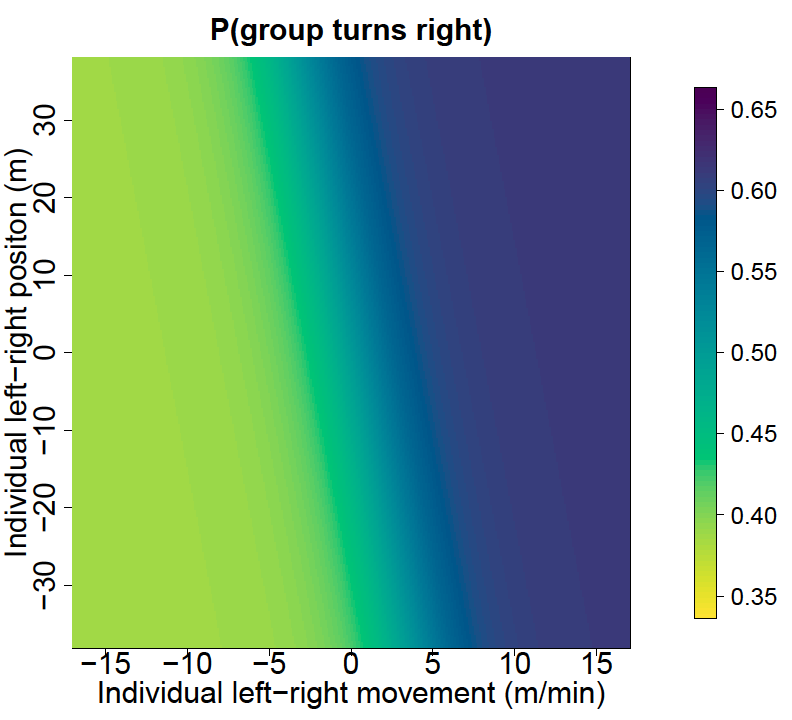


**A.**

**B.**

**4 – Distribution of group metrics**

The figures below show the distribution of three group-level metrics for each five groups: the **group spread**, calculated by averaging all distances between group members at any given moment, the **turning angle**, calculated as the angle between the past and future direction of movement of the group, and the **step duration**, calculated as the time taken by the group centroid to move 10m. For a given metric, x-axis is kept constant for easier comparison across groups.


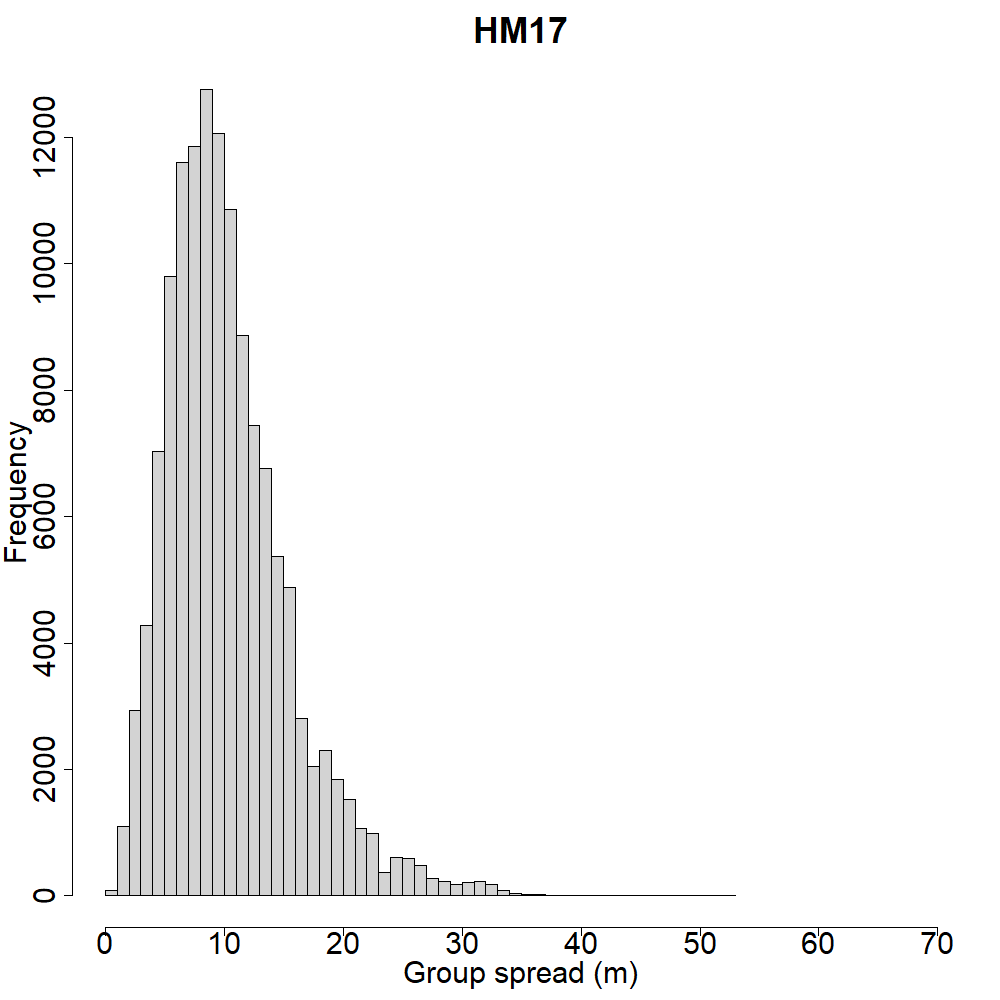

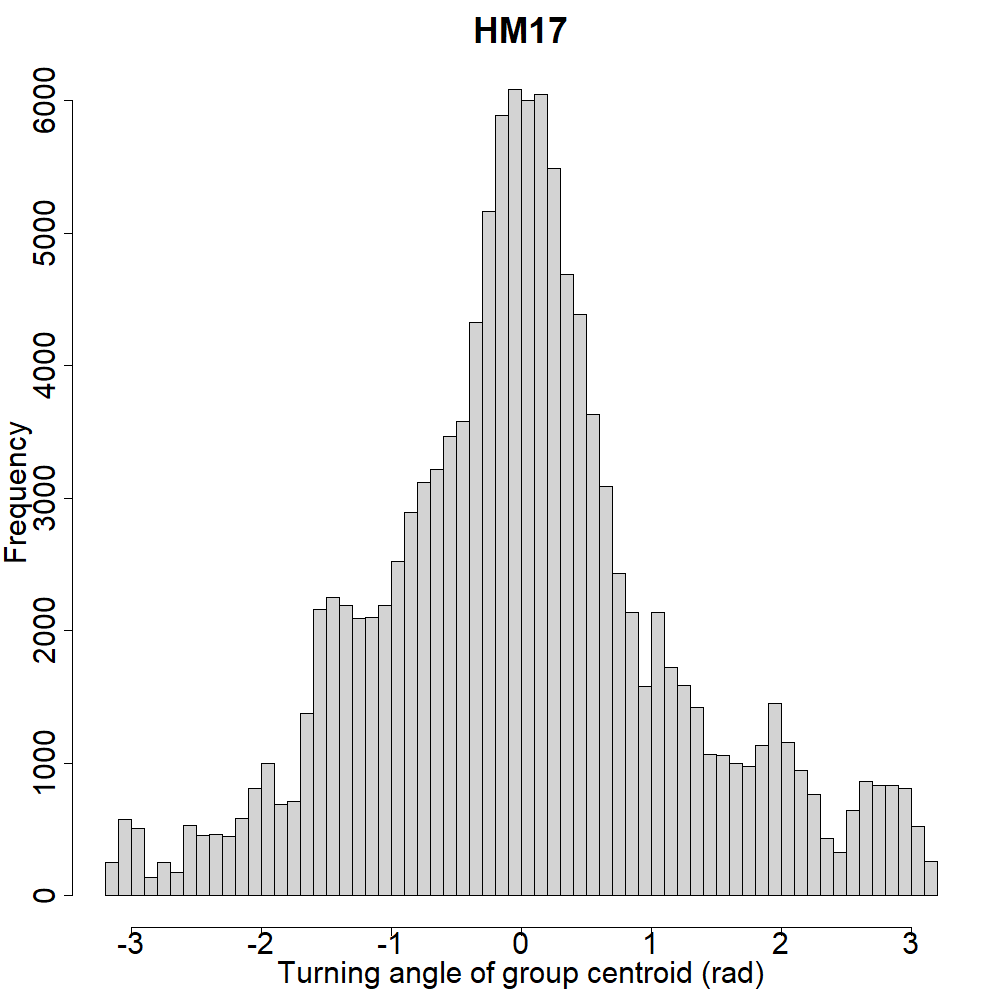

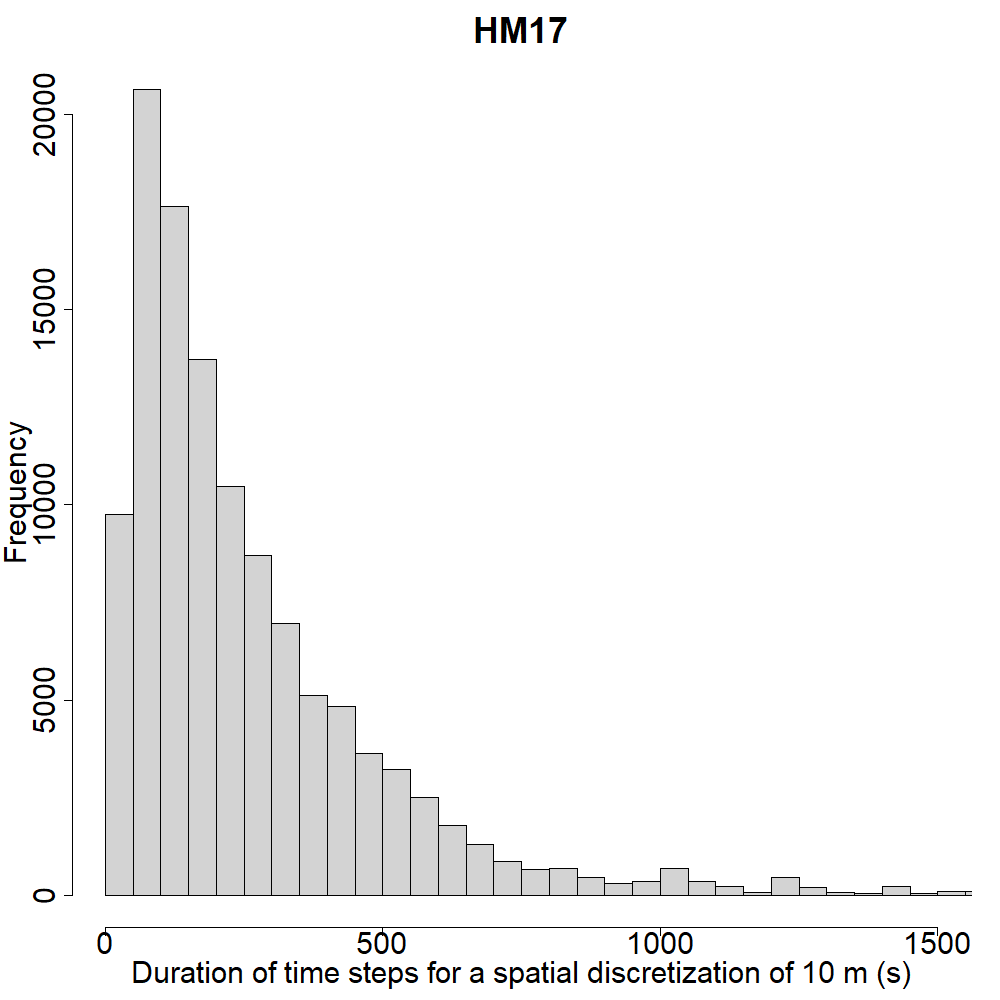

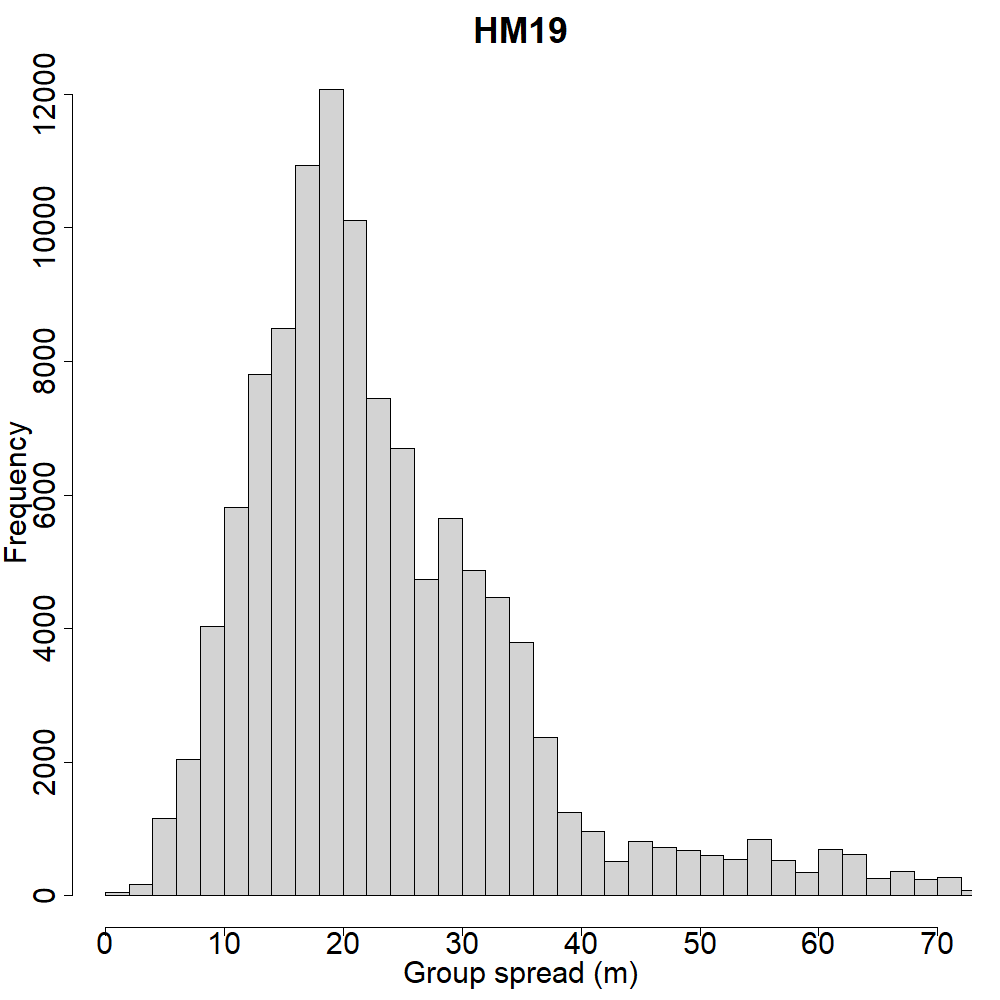

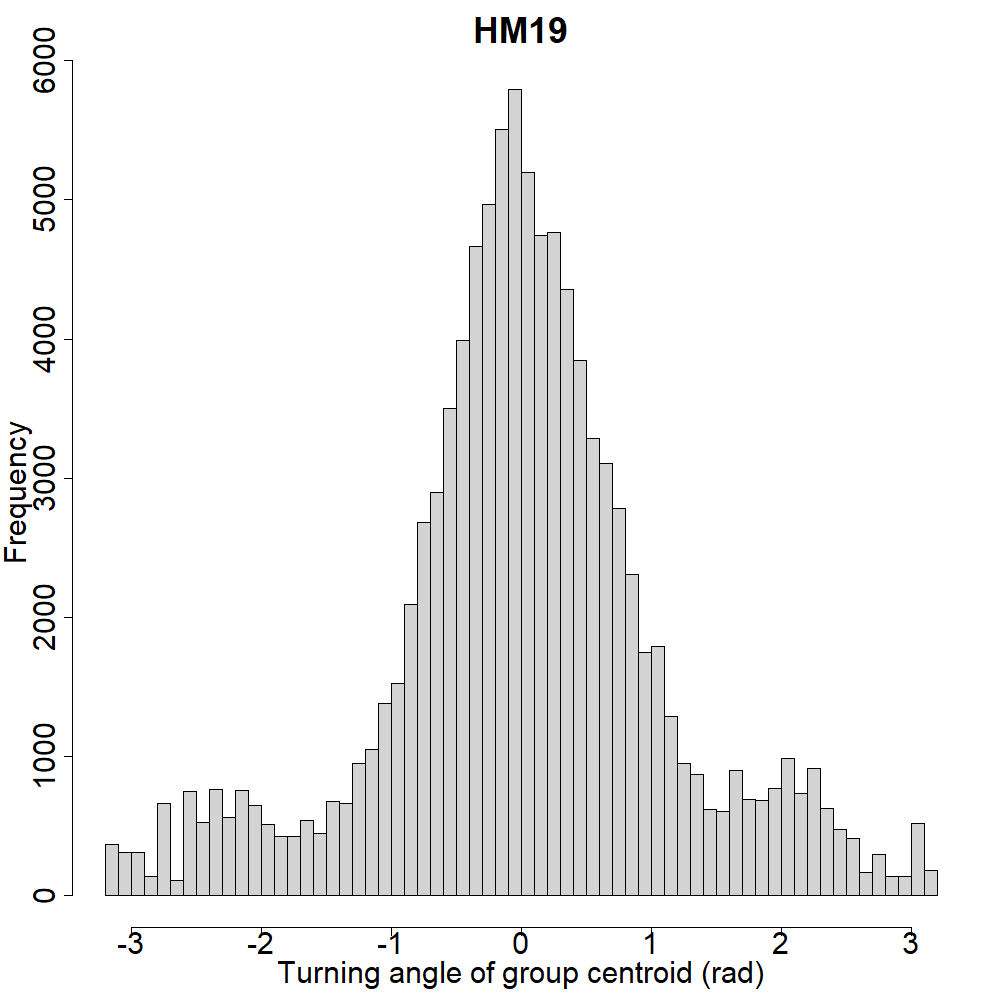

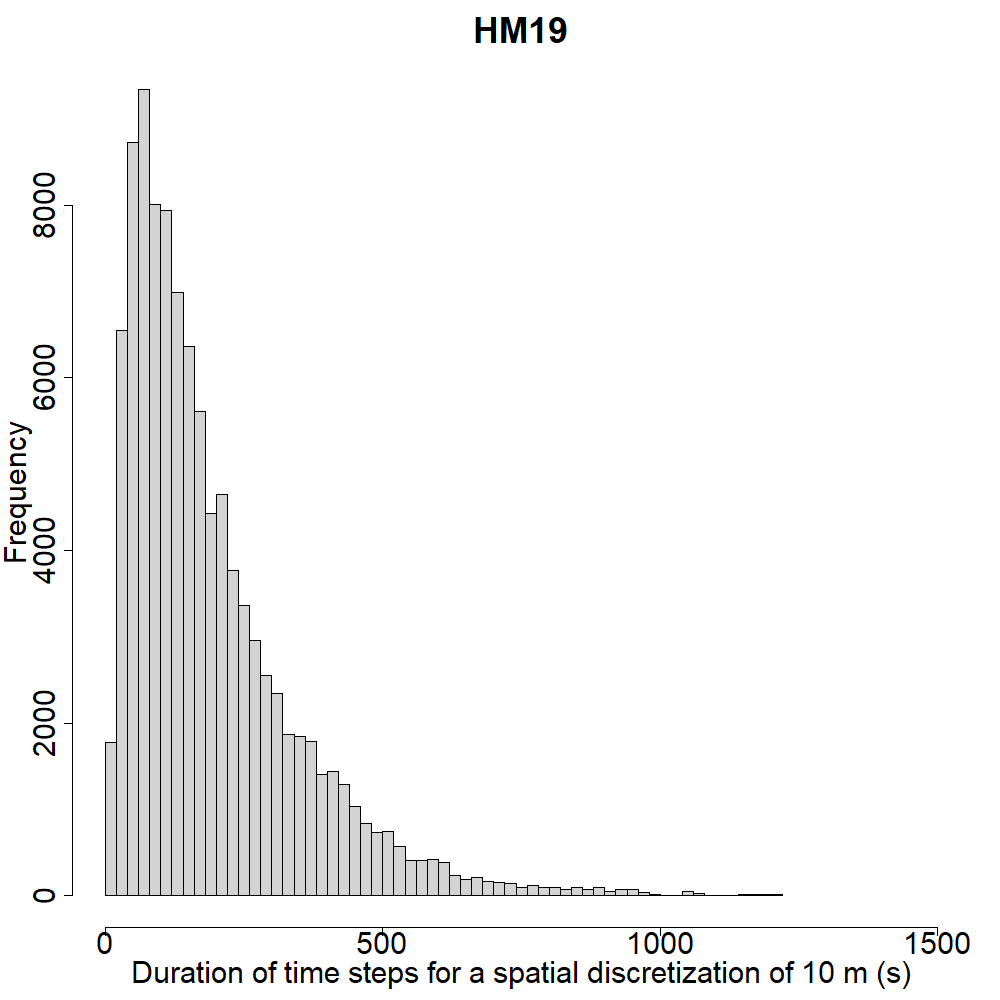

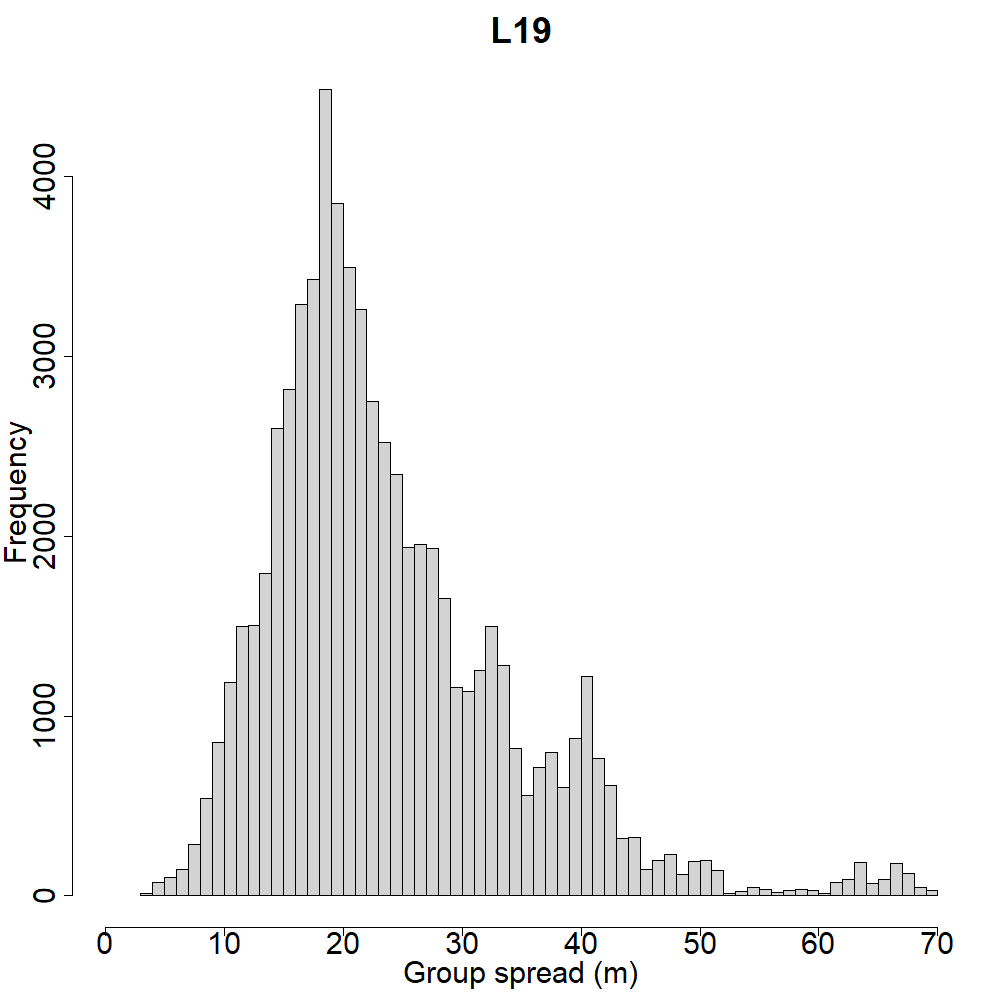

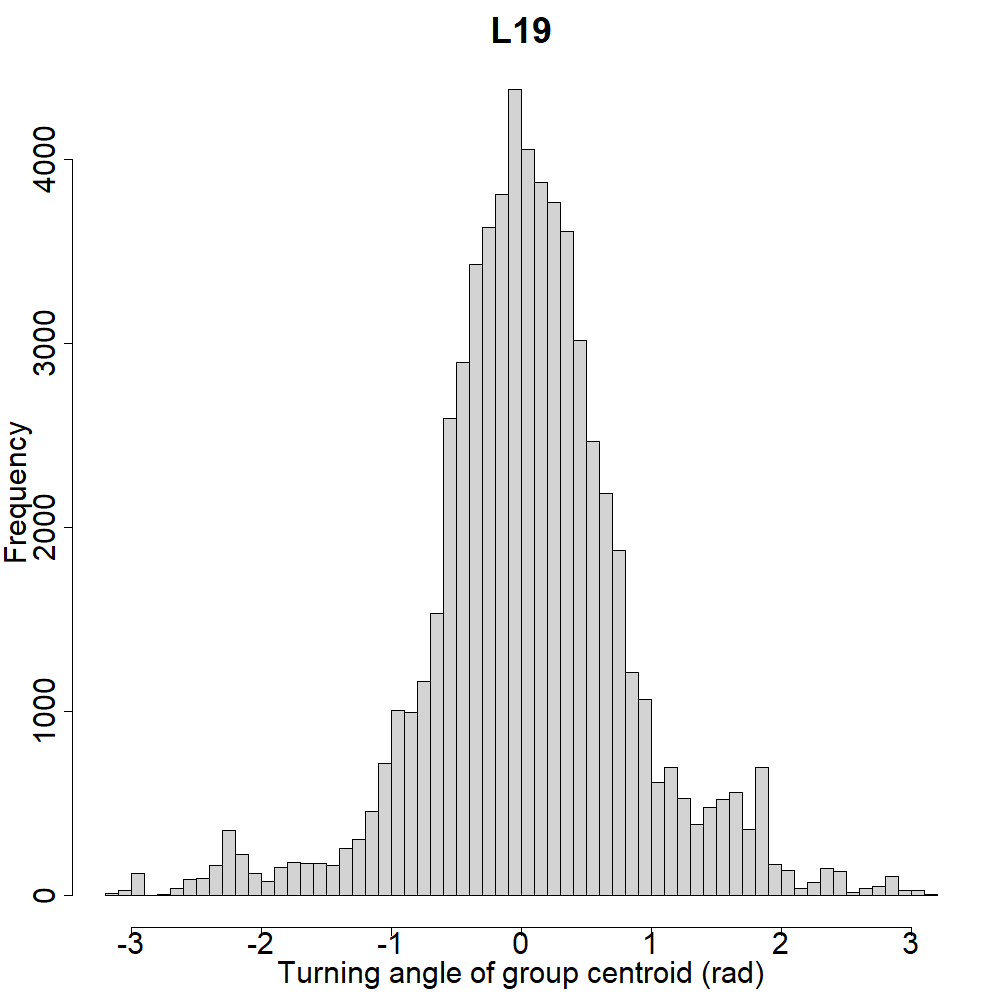

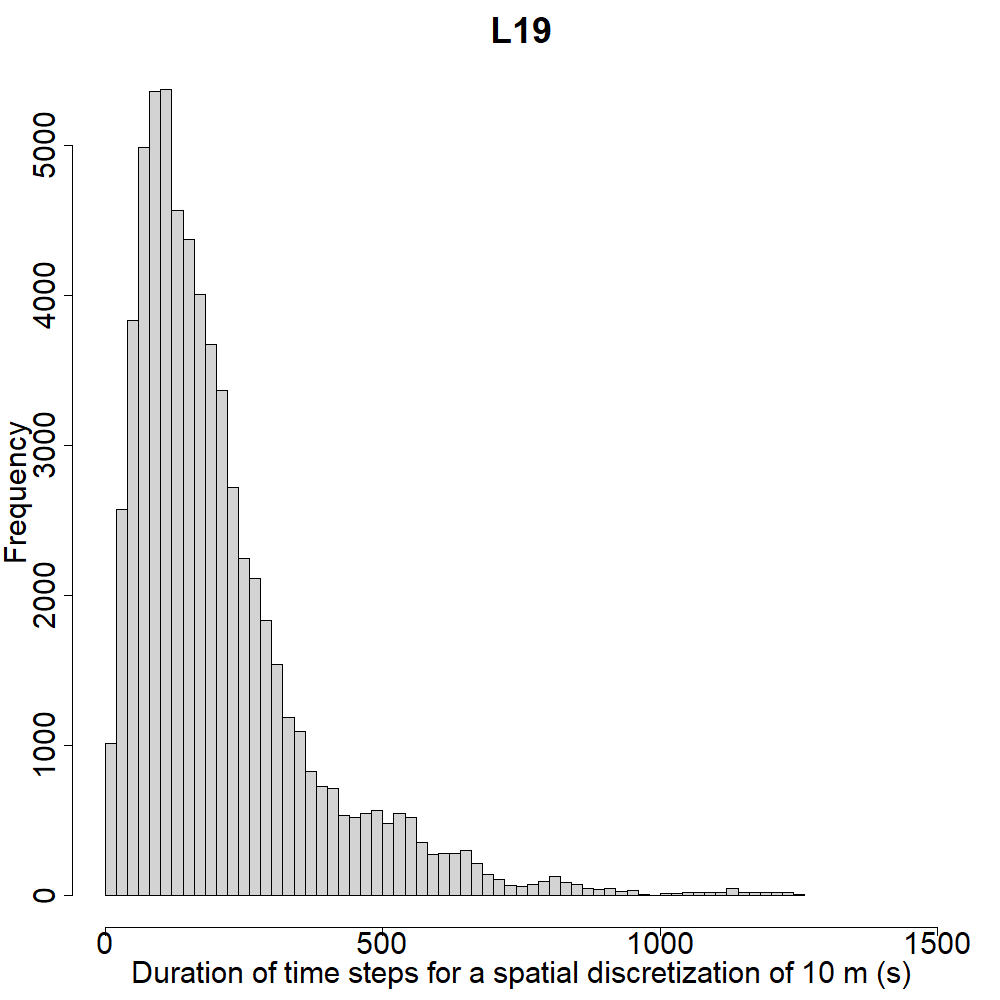

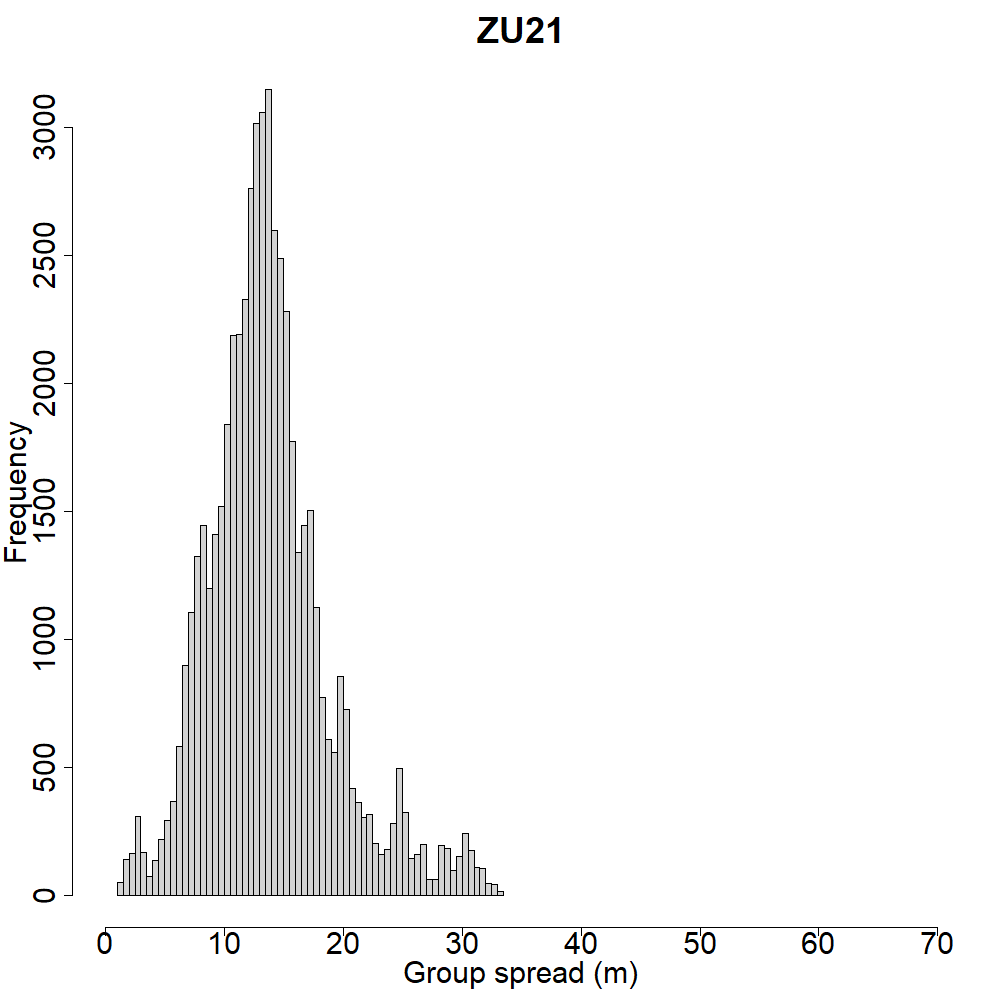

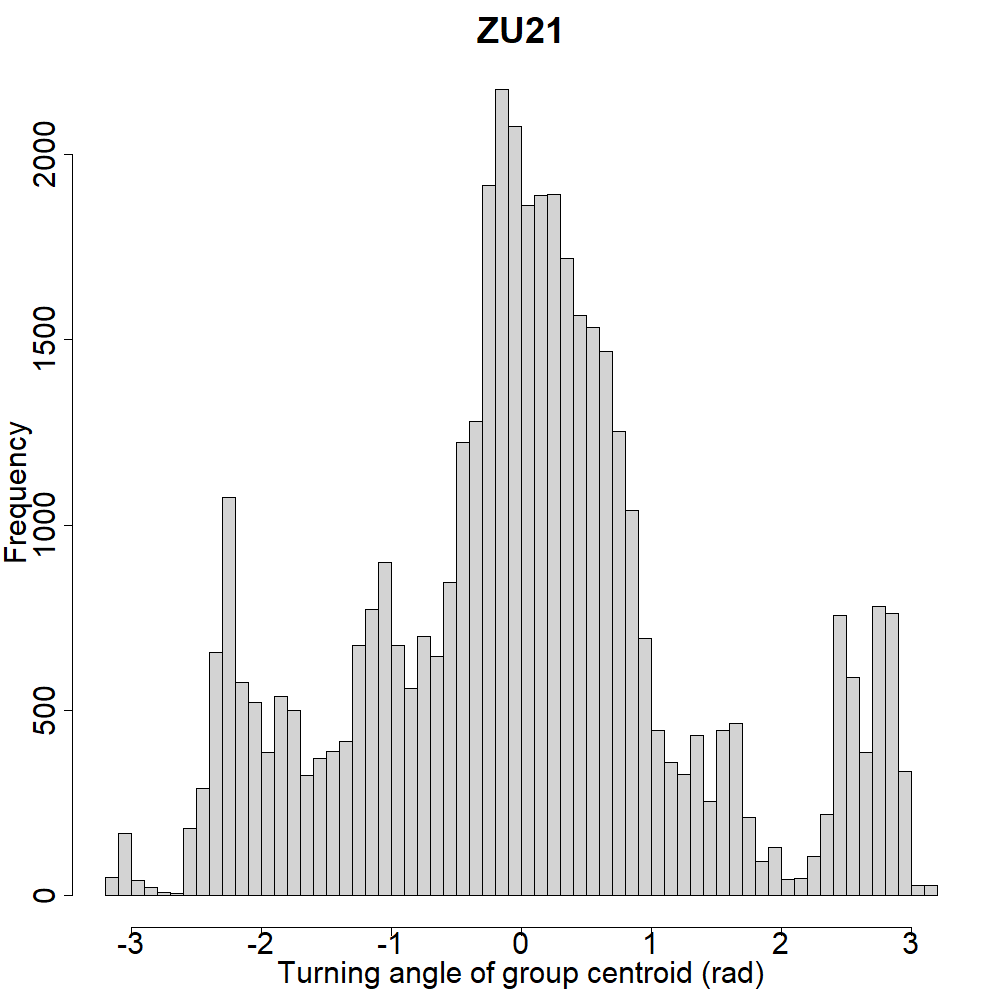

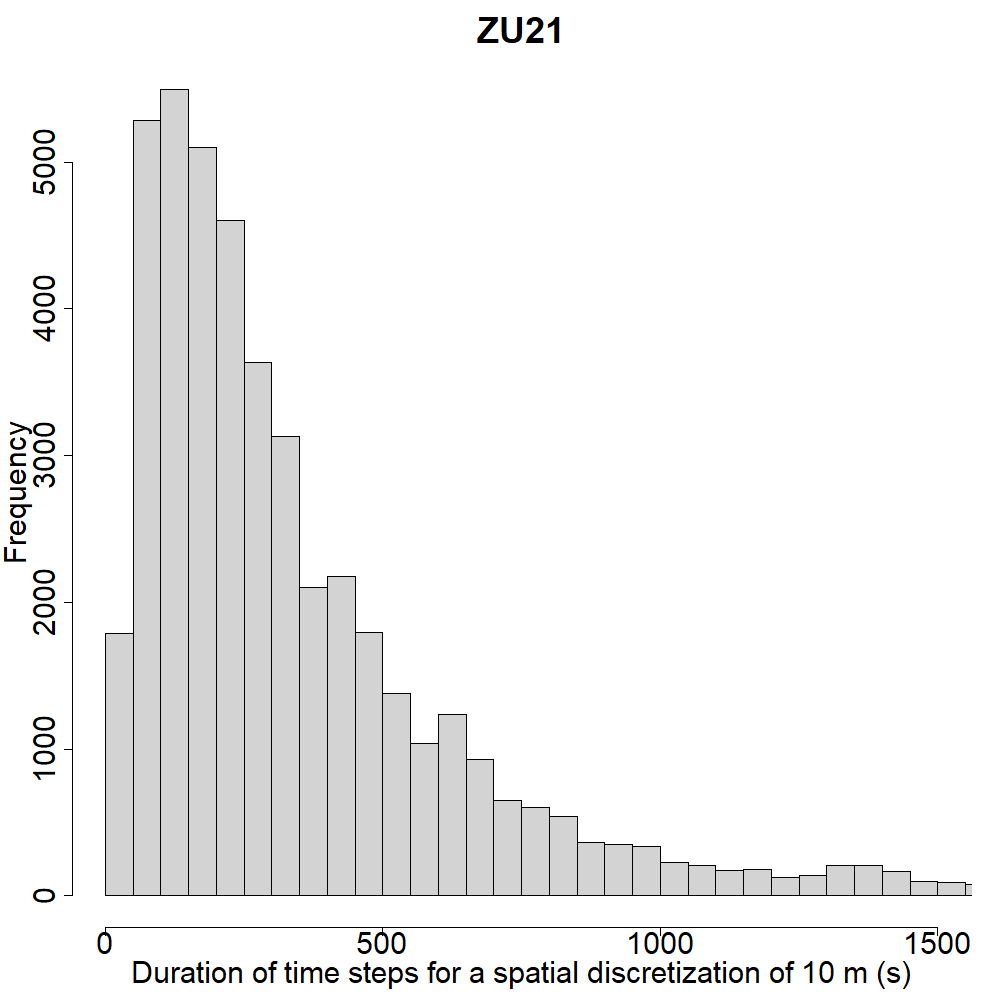

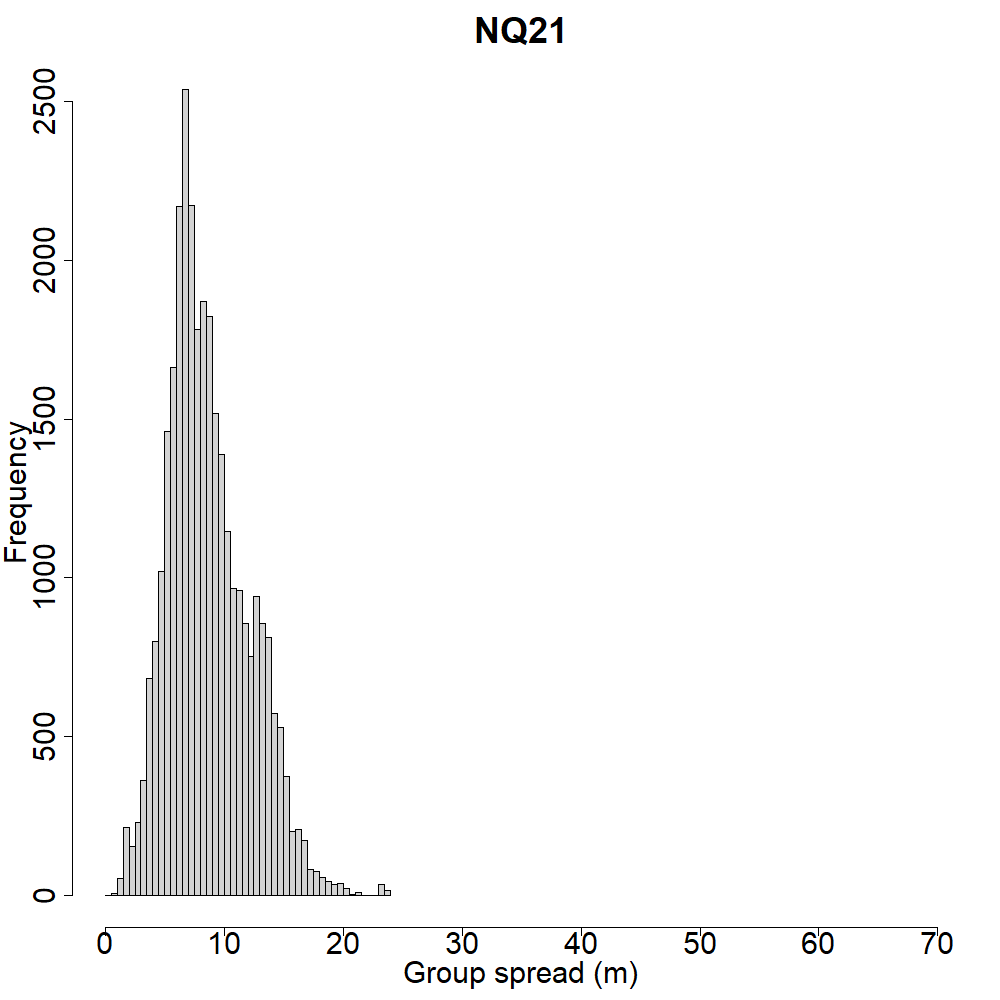

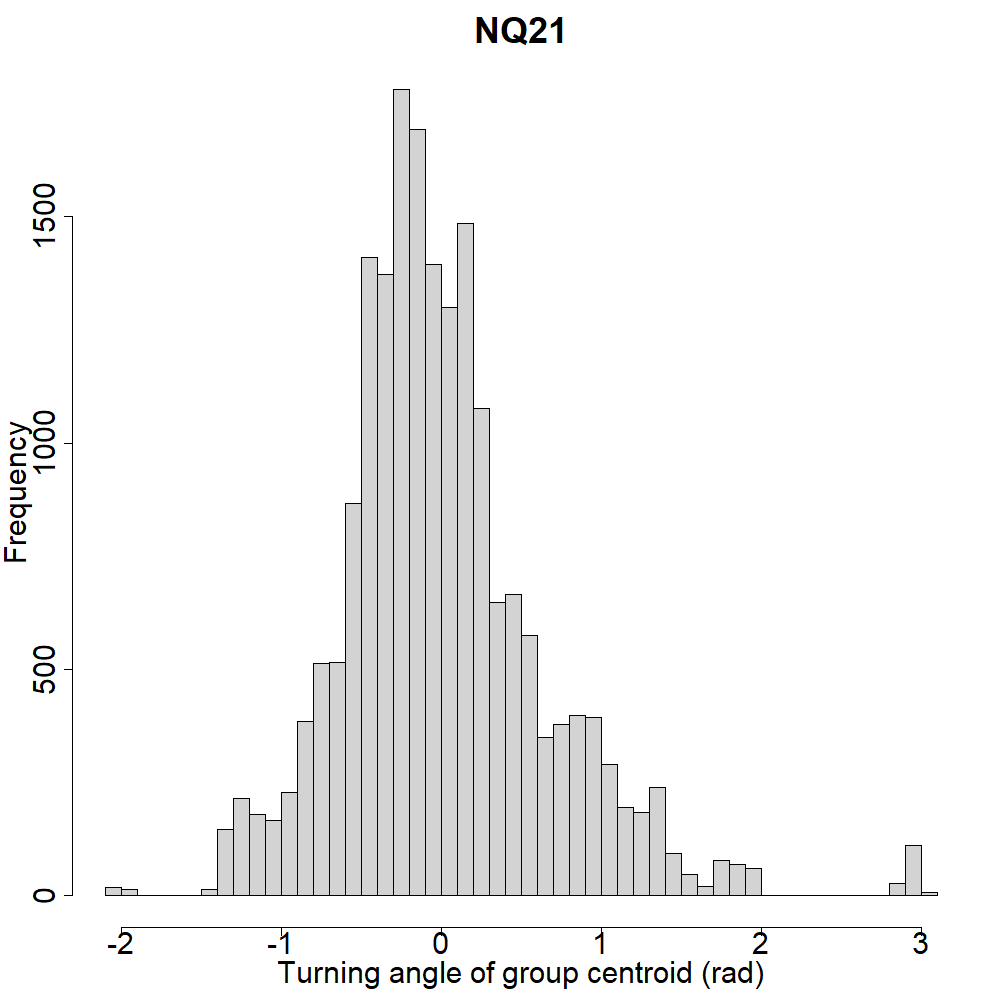

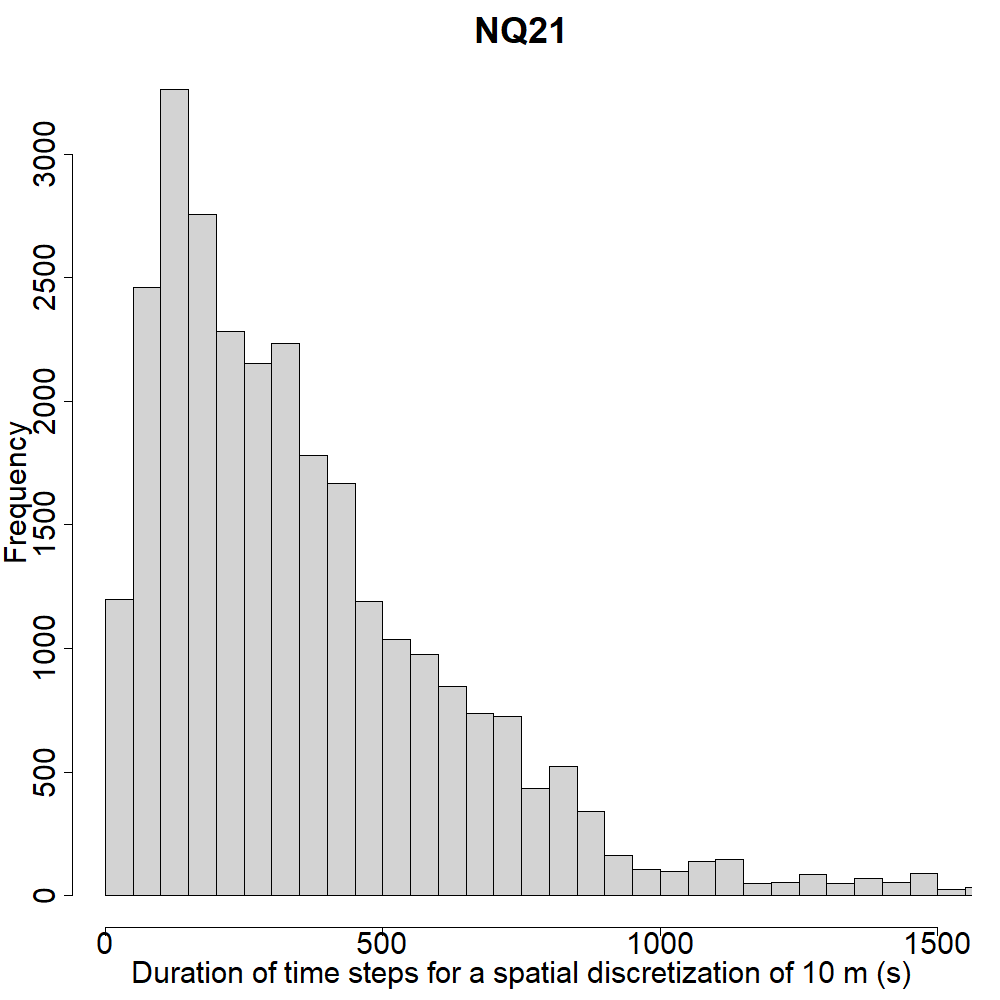


**5 - Robustness check: varying spatial discretization step**

Figures below show the individual turn influence scores, speed influence scores, and proportion of time spent in the front of the group, calculated with spatial discretization steps of 5, 15 and 20 meters, instead of 10 meters as presented in the main text. Plot layout is similar to that of Figures 3 and 4 in the main text.


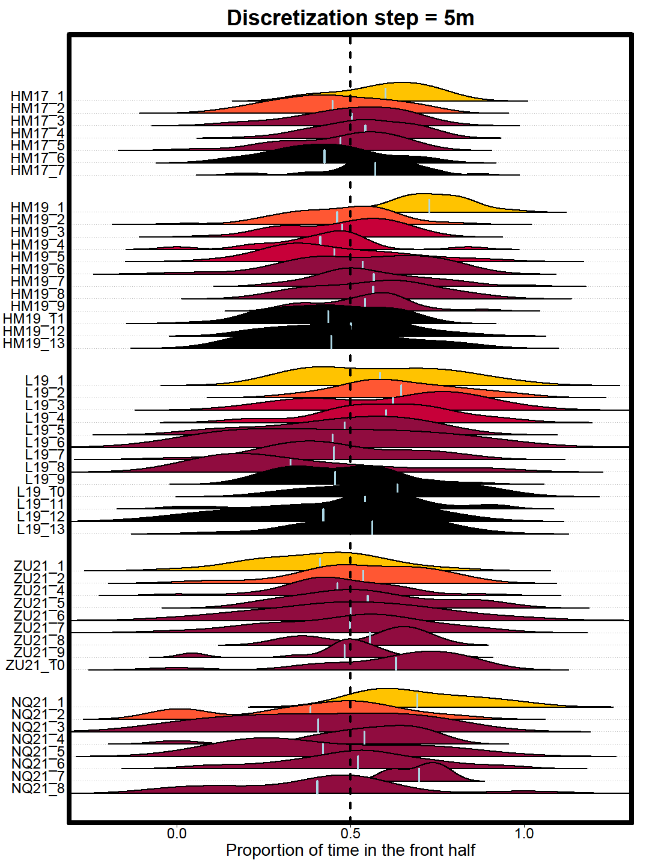

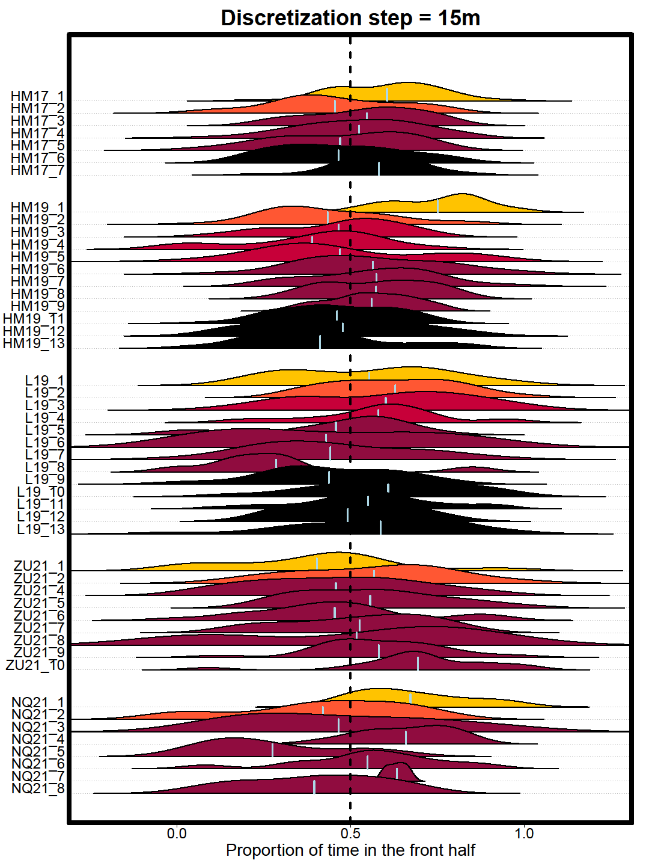

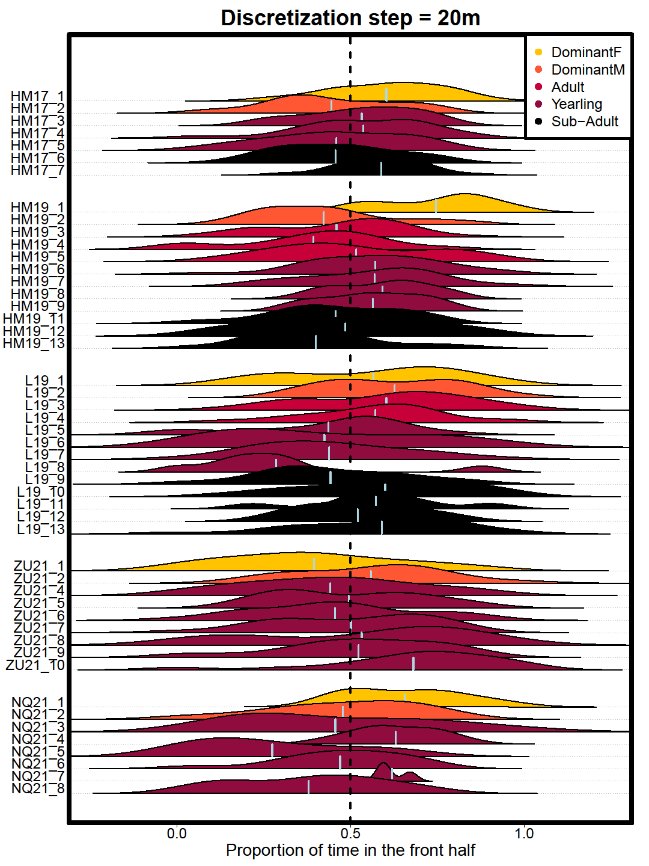


**6 – Statistical model fits**

Turn influence score as a function of status:

Table S4. Linear Mixed Effect models modelling turn influence scores as a function of social status. The dominant female status is the baseline factor.

|  | **Estimate** | **Std. Error** | **p-value** |
| --- | --- | --- | --- |
| Intercept | 0.666 | 0.018 | <0.001 |
| DominantM | -0.032 | 0.025 | 0.206 |
| Adult | -0.087 | 0.025 | 0.001 |
| Yearling | -0.081 | 0.020 | 0.002 |
| Sub-adult | -0.077 | 0.022 | 0.001 |

Table S5. Results of post-hoc Tukey contrasts comparing turn influence scores between the five social statuses. Each line tests the null hypothesis of no difference in the influence scores of the two statuses in the “comparison” column.

| **Comparison** | **Estimate** | **Std. Error** | **p-value** |
| --- | --- | --- | --- |
| DominantM - DominantF | -0.032 | 0.025 | 0.690 |
| Adult - DominantF | -0.087 | 0.025 | 0.005 |
| Yearling - DominantF | -0.081 | 0.020 | <0.001 |
| Sub-Adult - DominantF | -0.077 | 0.022 | 0.004 |
| Adult - DominantM | -0.054 | 0.025 | 0.195 |
| Yearling - DominantM | -0.049 | 0.020 | 0.091 |
| Sub-Adult - DominantM | -0.044 | 0.022 | 0.252 |
| Yearling - Adult | 0.005 | 0.020 | 0.999 |
| Sub-adult-Adult | 0.010 | 0.022 | 0.990 |
| Sub-Adult - Yearling | 0.005 | 0.015 | 0.998 |

Speed influence score as a function of status:

Table S6. Linear Mixed Effects models modelling speed influence scores as a function of social status. The dominant female status is the baseline factor.

|  | **Estimate** | **Std. Error** | **p-value** |
| --- | --- | --- | --- |
| Intercept | 0.474 | 0.040 | <0.001 |
| DominantM | -0.016 | 0.021 | 0.434 |
| Adult | -0.061 | 0.022 | 0.008 |
| Yearling | -0.062 | 0.016 | <0.001 |
| Sub-adult | -0.079 | 0.019 | <0.001 |

Table S7. Results of post-hoc Tukey contrasts comparing speed influence scores between the five social statuses. Each line tests the null hypothesis of no difference in the influence scores of the two statuses in the “comparison” column.

| **Comparison** | **Estimate** | **Std. Error** | **p-value** |
| --- | --- | --- | --- |
| DominantM - DominantF | -0.016 | 0.021 | 0.930 |
| Adult - DominantF | -0.061 | 0.022 | 0.040 |
| Yearling - DominantF | -0.062 | 0.016 | 0.001 |
| Sub-Adult - DominantF | -0.079 | 0.019 | <0.001 |
| Adult - DominantM | -0.044 | 0.022 | 0.238 |
| Yearling - DominantM | -0.046 | 0.016 | 0.035 |
| Sub-Adult - DominantM | -0.063 | 0.019 | 0.006 |
| Yearling - Adult | 0.001 | 0.018 | 0.999 |
| Sub-adult-Adult | -0.019 | 0.018 | 0.844 |
| Sub-Adult - Yearling | -0.017 | 0.014 | 0.726 |

Frontness as a function of status:

Table S8. Linear Mixed Effect models modelling the proportion of times spent in the front of the group (“frontness”) as a function of social status. The dominant female status is the baseline factor.

|  | **Estimate** | **Std. Error** | **p-value** |
| --- | --- | --- | --- |
| Intercept | 0.602 | 0.017 | <0.001 |
| DominantM | -0.110 | 0.025 | <0.001 |
| Adult | -0.089 | 0.025 | <0.001 |
| Yearling | -0.083 | 0.020 | <0.001 |
| Sub-adult | -0.100 | 0.021 | <0.001 |

Turn influence score as a function of status and frontness:

Table S9. Linear Mixed Effect models modelling turn influence scores as a function of social status and total proportion of time spent in the front of the group (”frontness”). The dominant female status is the baseline factor.

|  | **Estimate** | **Std. Error** | **p-value** |
| --- | --- | --- | --- |
| Intercept | 0.581 | 0.040 | <0.001 |
| DominantM | -0.019 | 0.024 | 0.440 |
| Adult | -0.074 | 0.025 | 0.004 |
| Yearling | -0.070 | 0.019 | <0.001 |
| Sub-adult | -0.065 | 0.021 | 0.004 |
| Frontness | 0.145 | 0.062 | 0.024 |

Table S10. Results of post-hoc Tukey contrasts comparing turn influence scores between the five social statuses. Each line tests the null hypothesis of no difference in the influence scores of the two statuses in the “comparison” column.

| **Comparison** | **Estimate** | **Std. Error** | **p-value** |
| --- | --- | --- | --- |
| DominantM – DominantF | -0.019 | 0.024 | 0.6933 |
| Adult – DominantF | -0.074 | 0.025 | 0.020 |
| Yearling – DominantF | -0.070 | 0.019 | 0.002 |
| Sub-Adult – DominantF | -0.065 | 0.021 | 0.018 |
| Adult – DominantM | -0.055 | 0.024 | 0.141 |
| Yearling – DominantM | -0.051 | 0.019 | 0.047 |
| Sub-Adult – DominantM | -0.046 | 0.021 | 0.165 |
| Yearling – Adult | 0.005 | 0.019 | 0.999 |
| Sub-adult-Adult | 0.009 | 0.021 | 0.992 |
| Sub-Adult – Yearling | 0.004 | 0.015 | 0.998 |

Speed influence score as a function of status and frontness:

Table S11. Linear Mixed Effects models modelling speed influence scores as a function of social status and total proportion of time spent in the front of the group.(=”frontness”). The dominant female status is the baseline factor.

|  | **Estimate** | **Std. Error** | **p-value** |
| --- | --- | --- | --- |
| Intercept | 0.443 | 0.051 | <0.001 |
| DominantM | -0.011 | 0.021 | 0.593 |
| Adult | -0.056 | 0.022 | 0.015 |
| Yearling | -0.058 | 0.017 | 0.001 |
| Sub-adult | -0.075 | 0.019 | <0.001 |
| Frontness | 0.053 | 0.054 | 0.331 |

Table S12. Results of post-hoc Tukey contrasts comparing speed influence scores between the five social statuses. Each line tests the null hypothesis of no difference in the influence scores of the two statuses in the “comparison” column.

| **Comparison** | **Estimate** | **Std. Error** | **p-value** |
| --- | --- | --- | --- |
| DominantM - DominantF | -0.011 | 0.021 | 0.982 |
| Adult - DominantF | -0.056 | 0.022 | 0.080 |
| Yearling - DominantF | -0.058 | 0.017 | 0.001 |
| Sub-Adult - DominantF | -0.075 | 0.019 | <0.001 |
| Adult - DominantM | -0.045 | 0.022 | 0.230 |
| Yearling - DominantM | -0.047 | 0.016 | 0.031 |
| Sub-Adult - DominantM | -0.063 | 0.019 | 0.005 |
| Yearling - Adult | -0.002 | 0.018 | 0.999 |
| Sub-adult-Adult | -0.019 | 0.018 | 0.841 |
| Sub-Adult - Yearling | -0.017 | 0.014 | 0.742 |

**7 – Individual fits for the four types of influence**

The figures below show the outcome of the model fit for each individual and each of the four types of influence. Each blue frame represents one individual and within each frame, the top-left plot shows the fit for the positional turn influence, the top-right plot shows the fit for the regular (movement-based) turn influence, the bottom-left plot shows the fit for the positional speed influence, the bottom-right plot shows the fit for the regular (movement-based) speed influence. In each plot, the black curve represents the binned data, with dot size indicating number of time steps used as shown in the bottom-right legend, the red curve shows the fitted line from our model, blue dotted lines show how the influence score is found. Top-left legend indicates the fitted parameter of the model, its log-likelihood, and the individual influence score. X-axis limits extend to the 0.01-0.99% quantiles of each variable to show them on comparable scales.

**
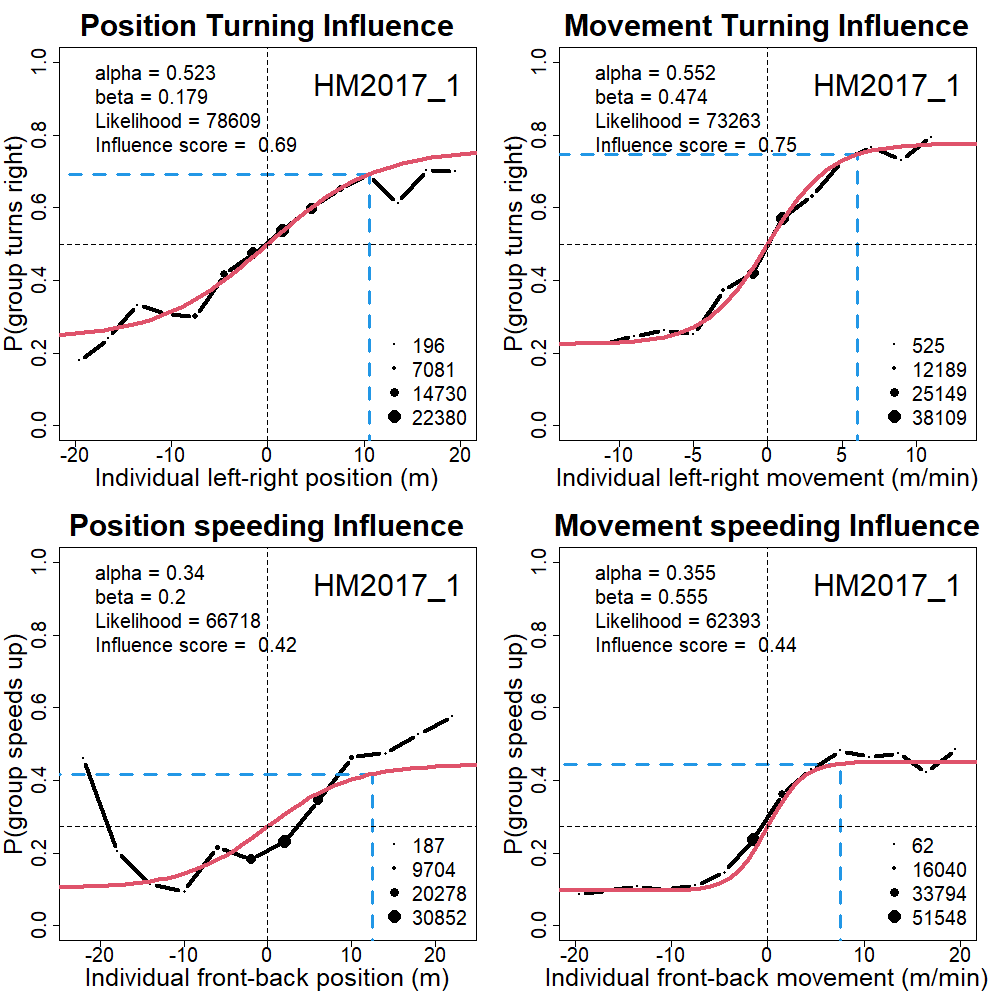

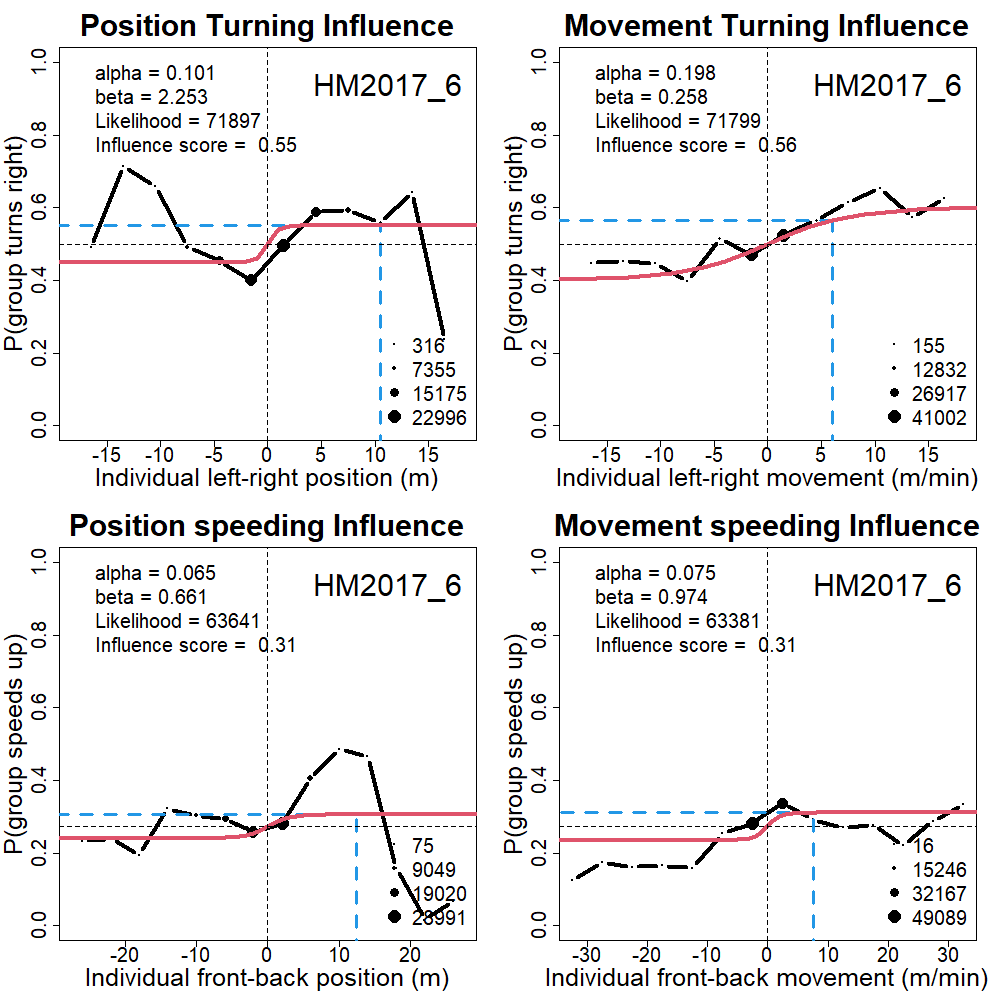

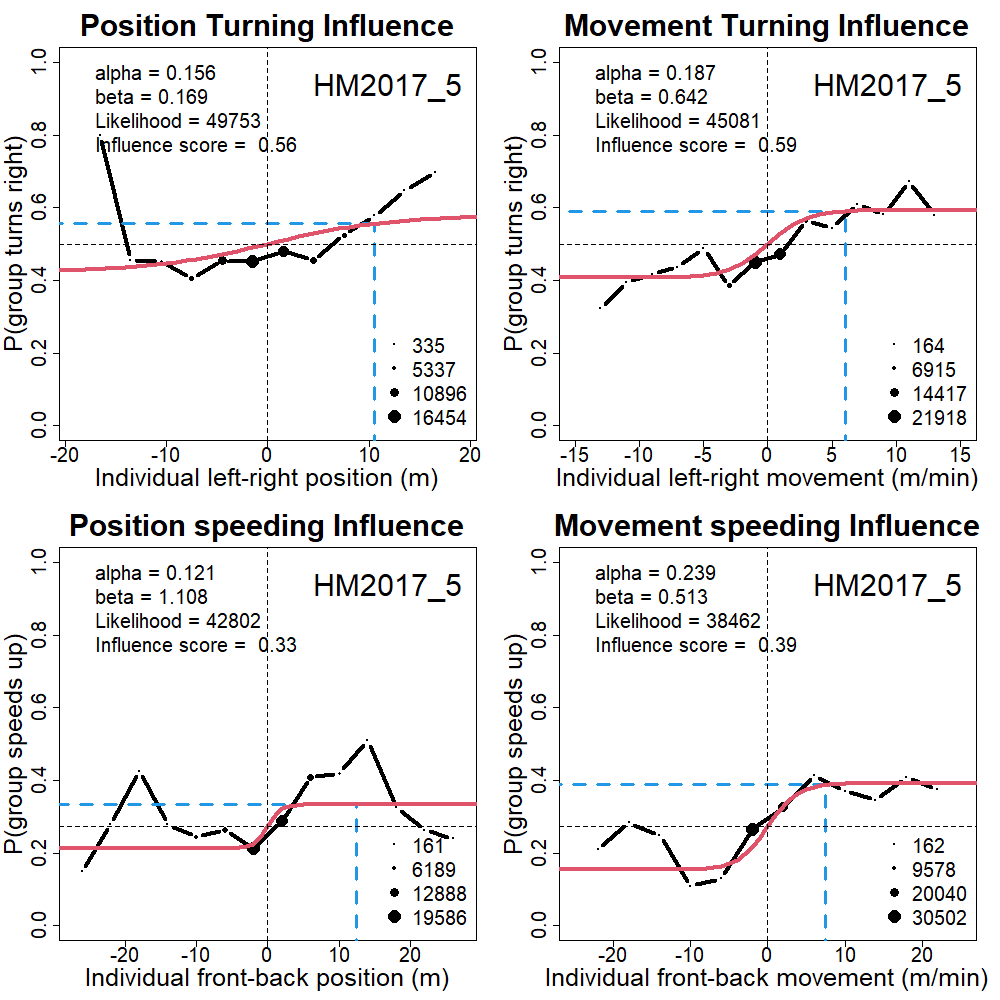

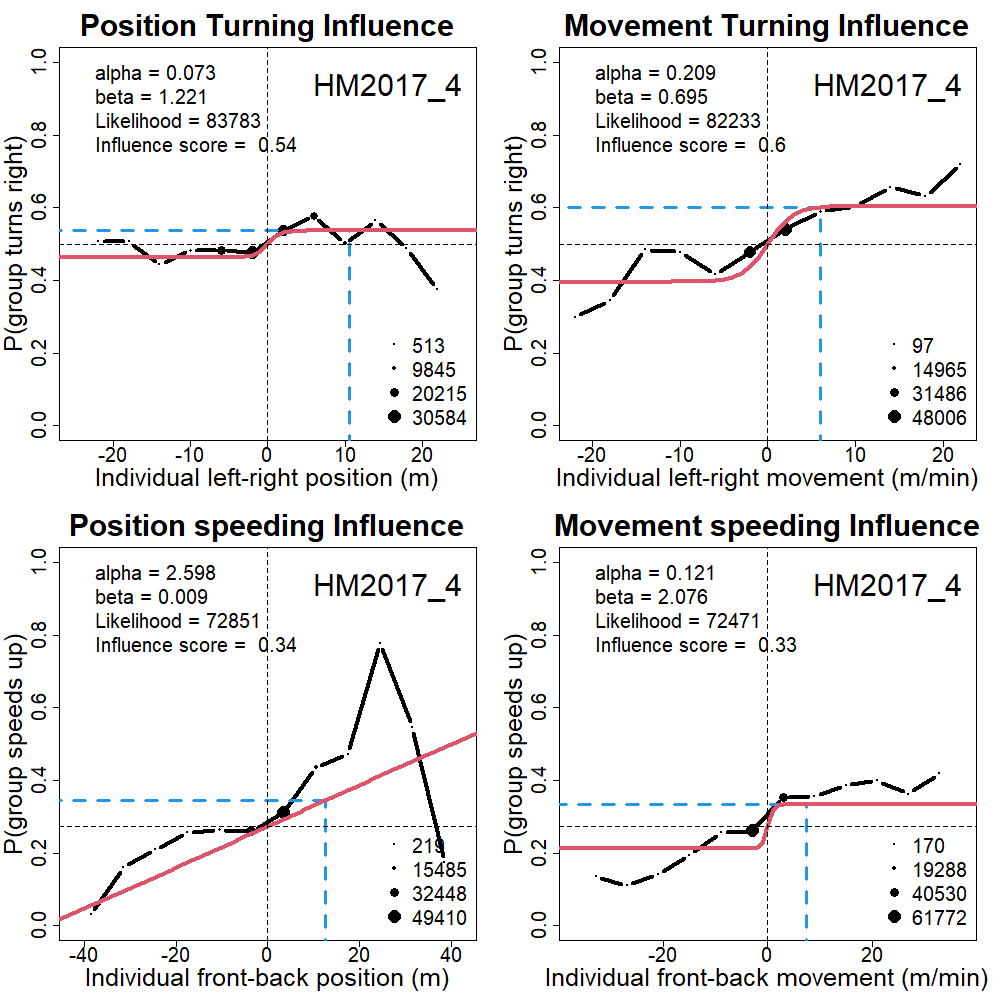

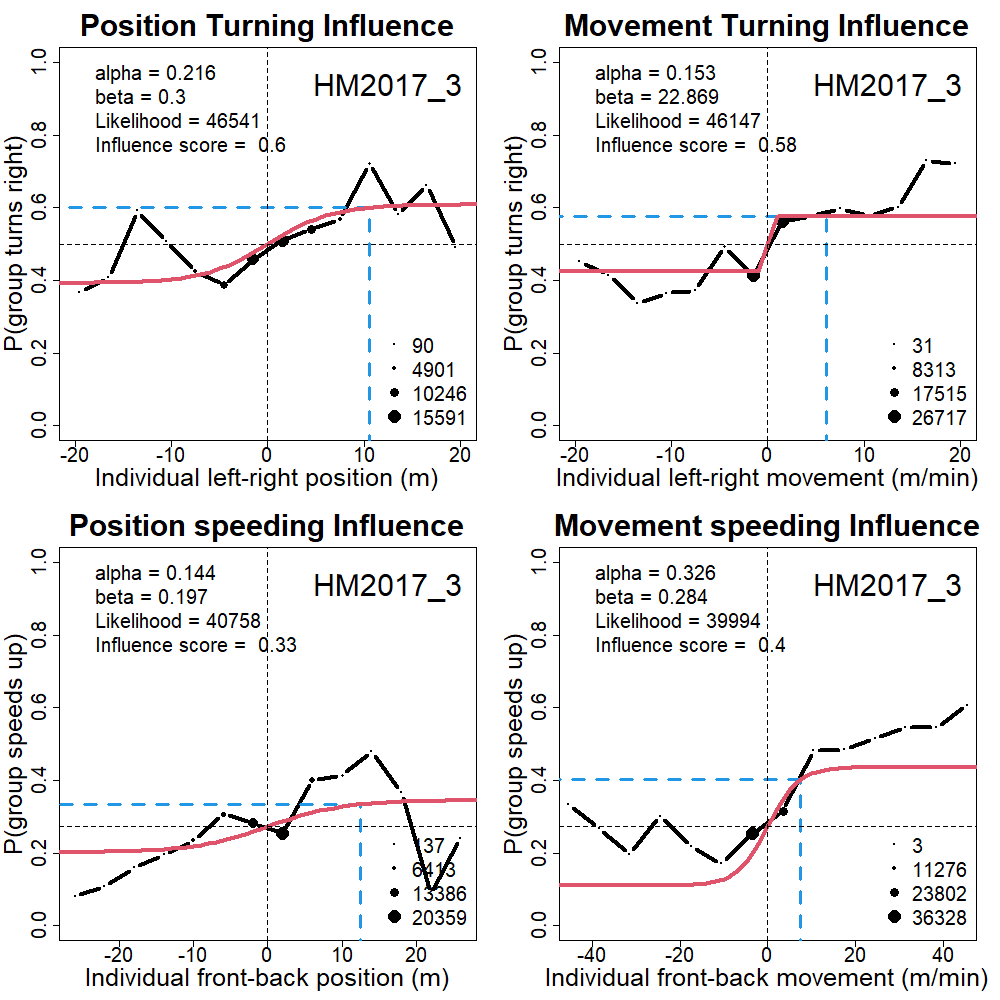

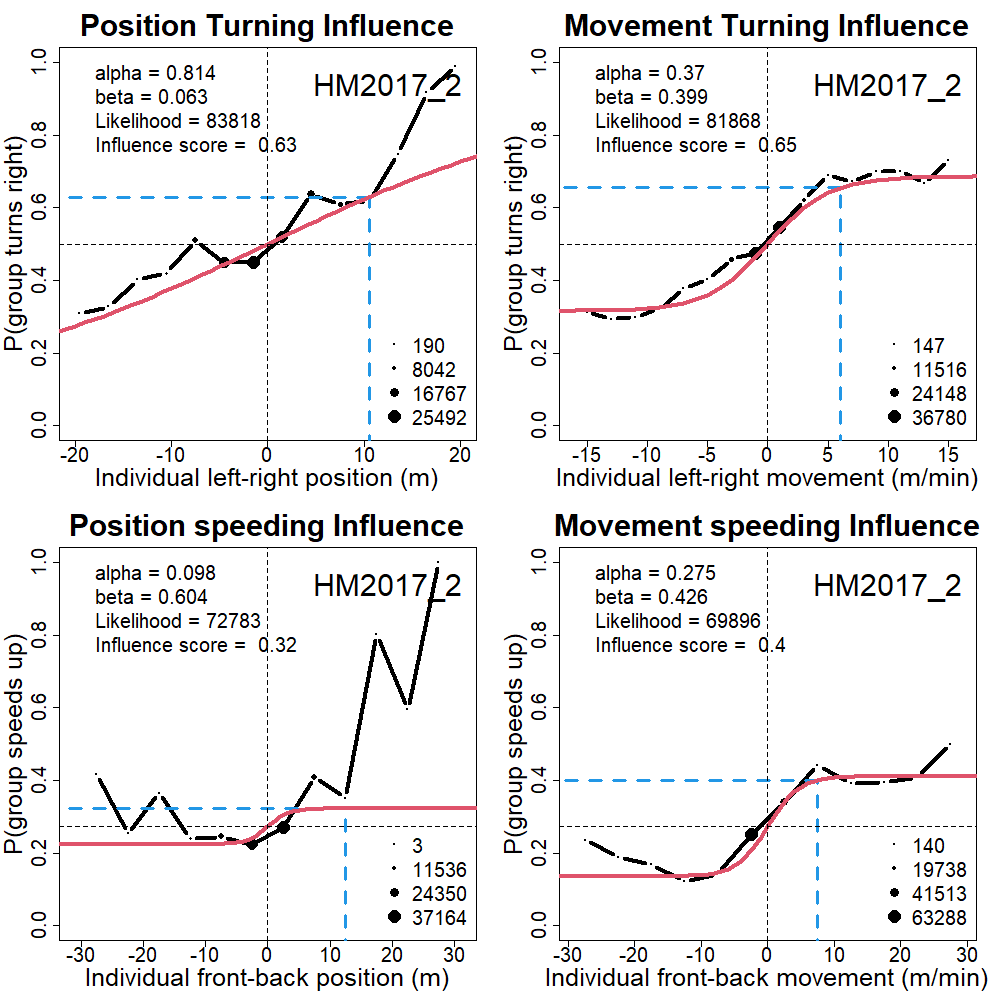
**

**
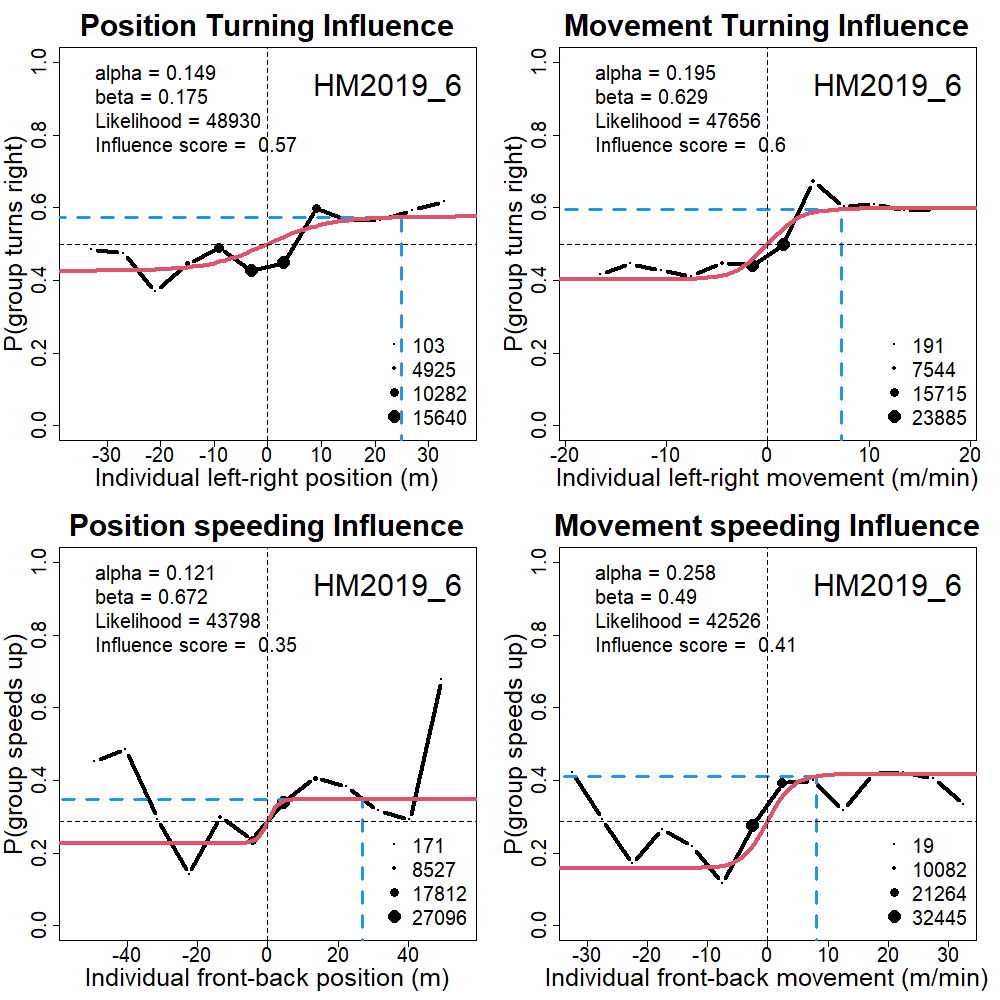

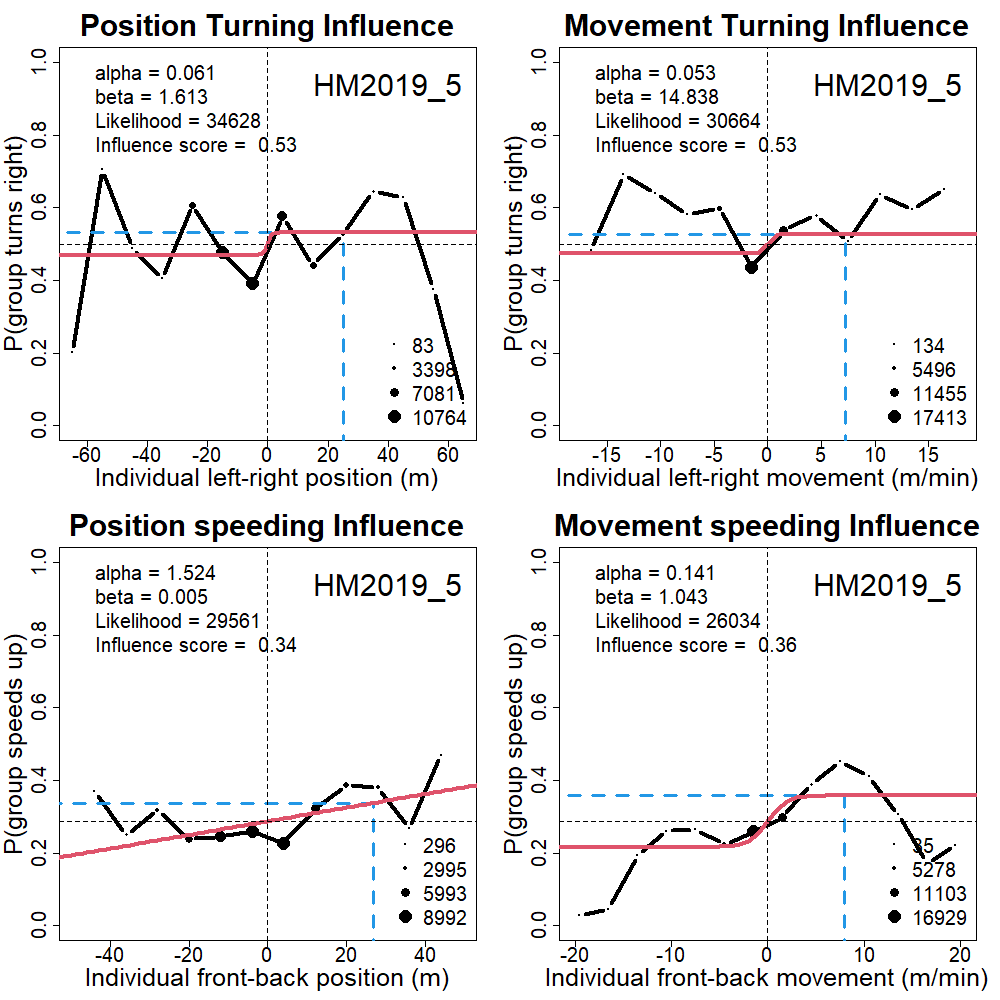

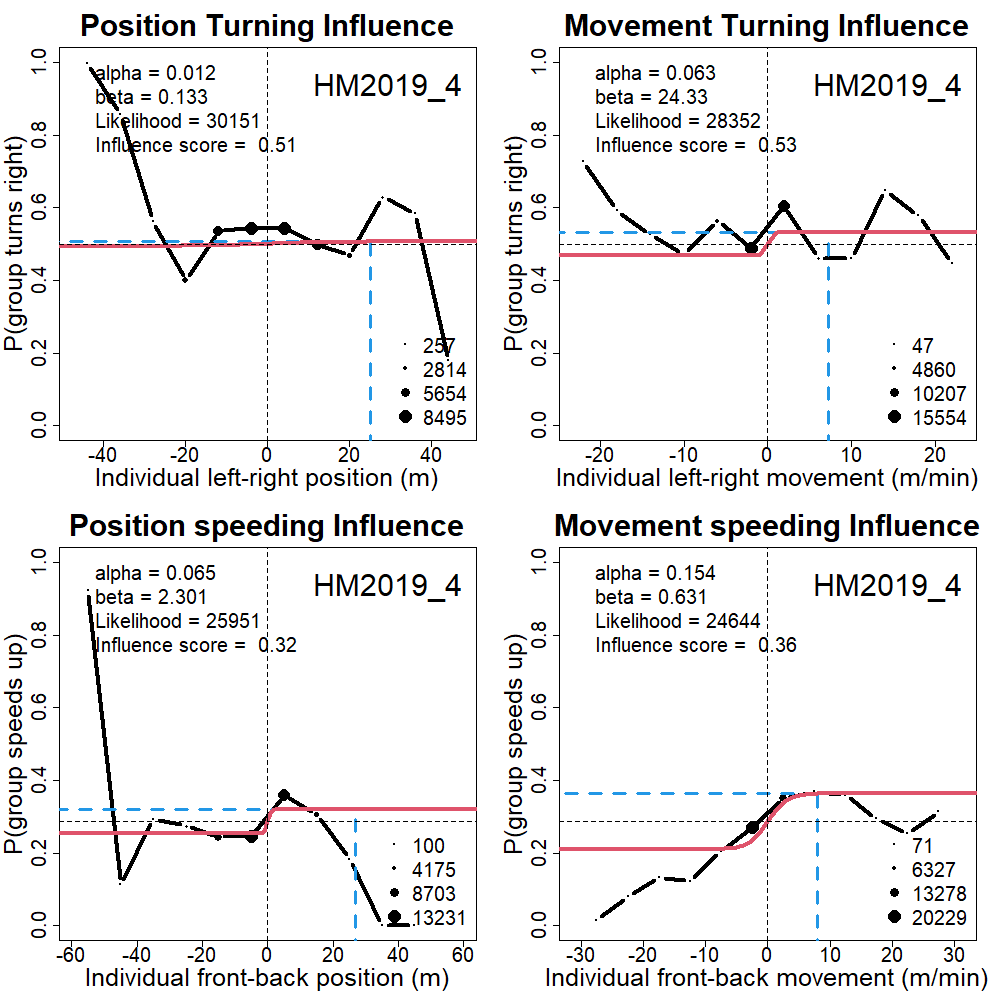

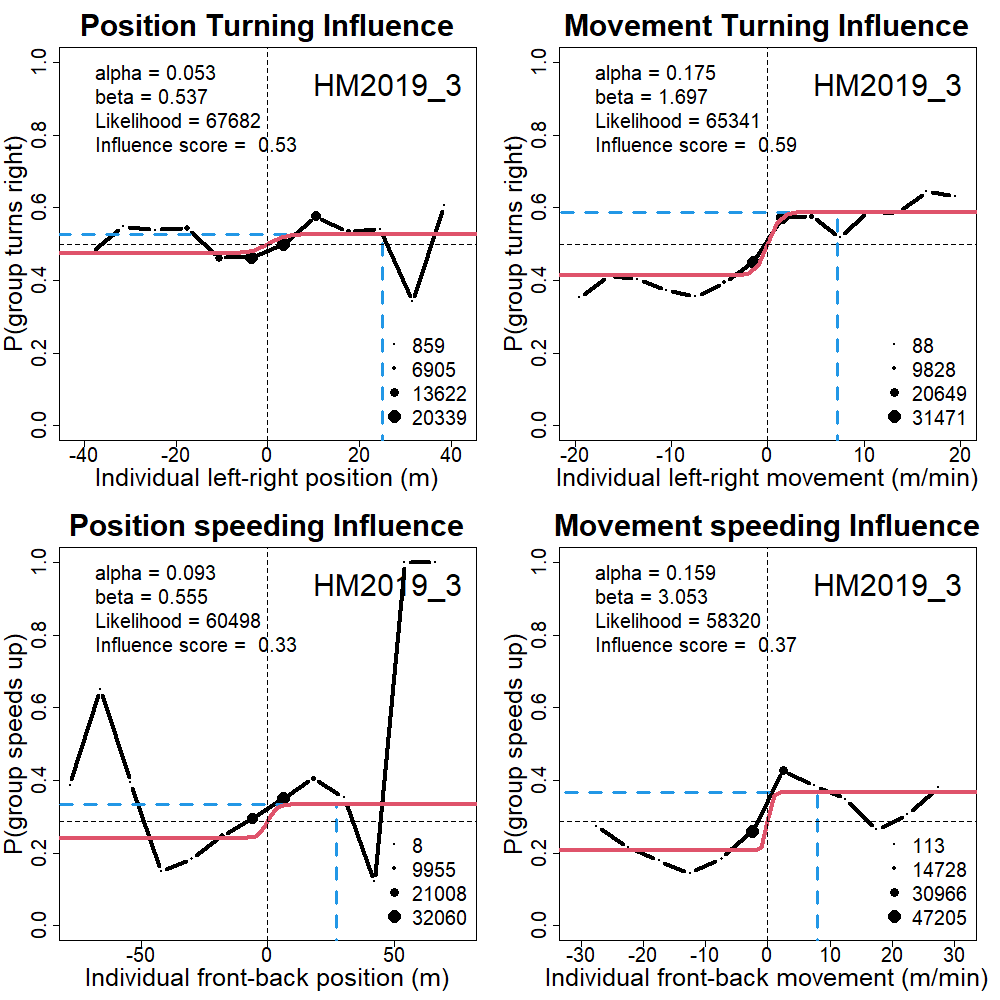

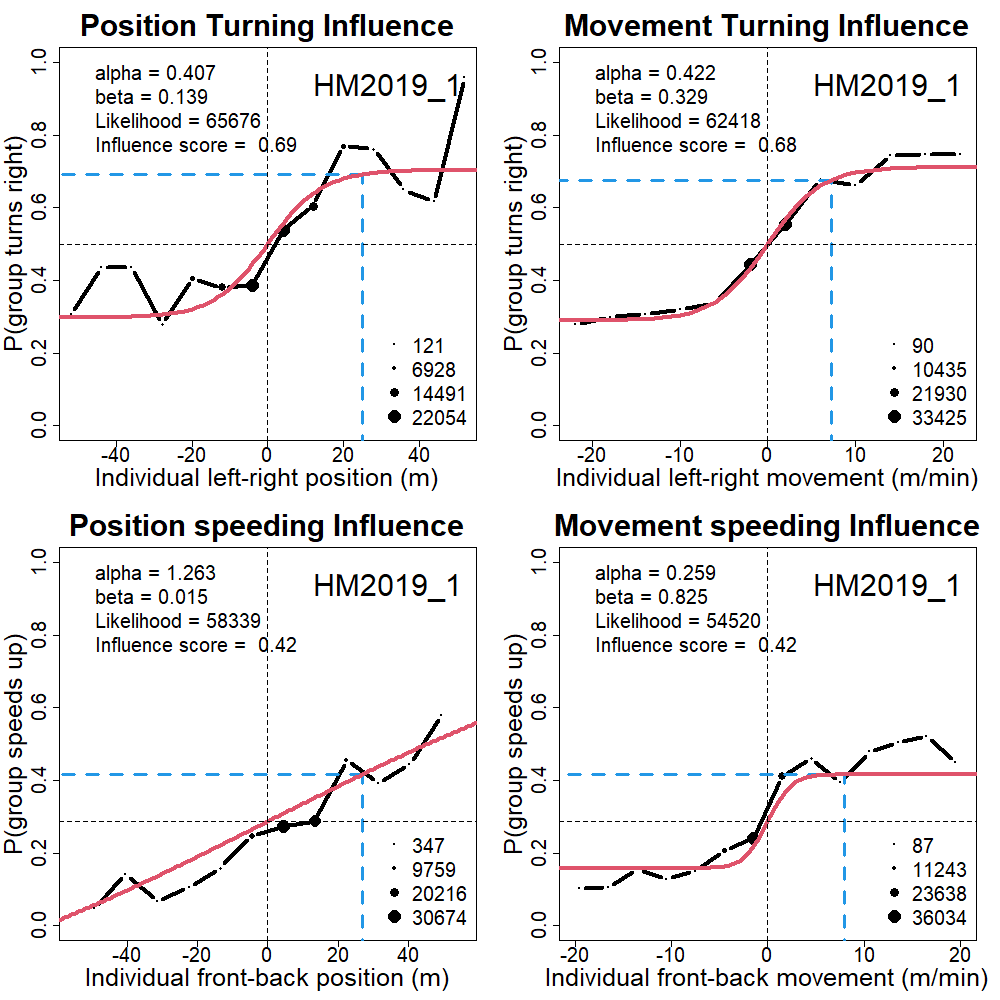

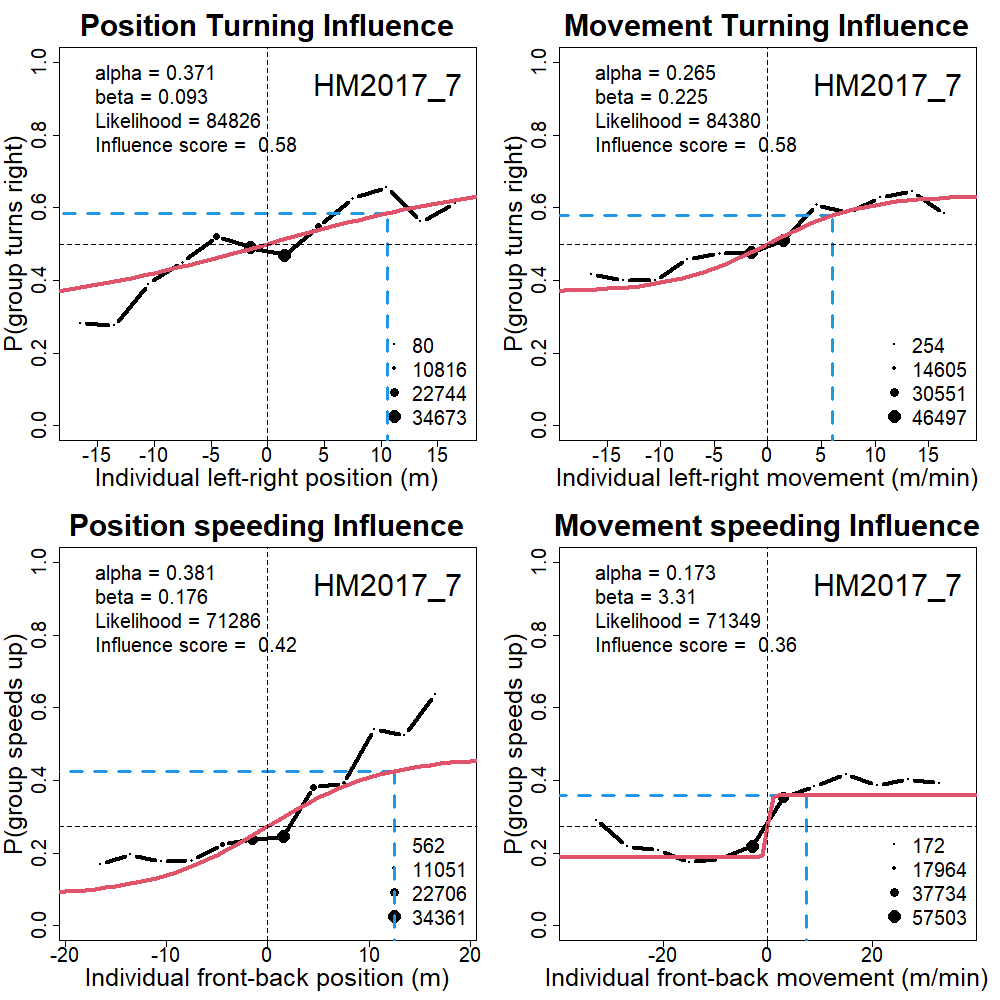
**

**
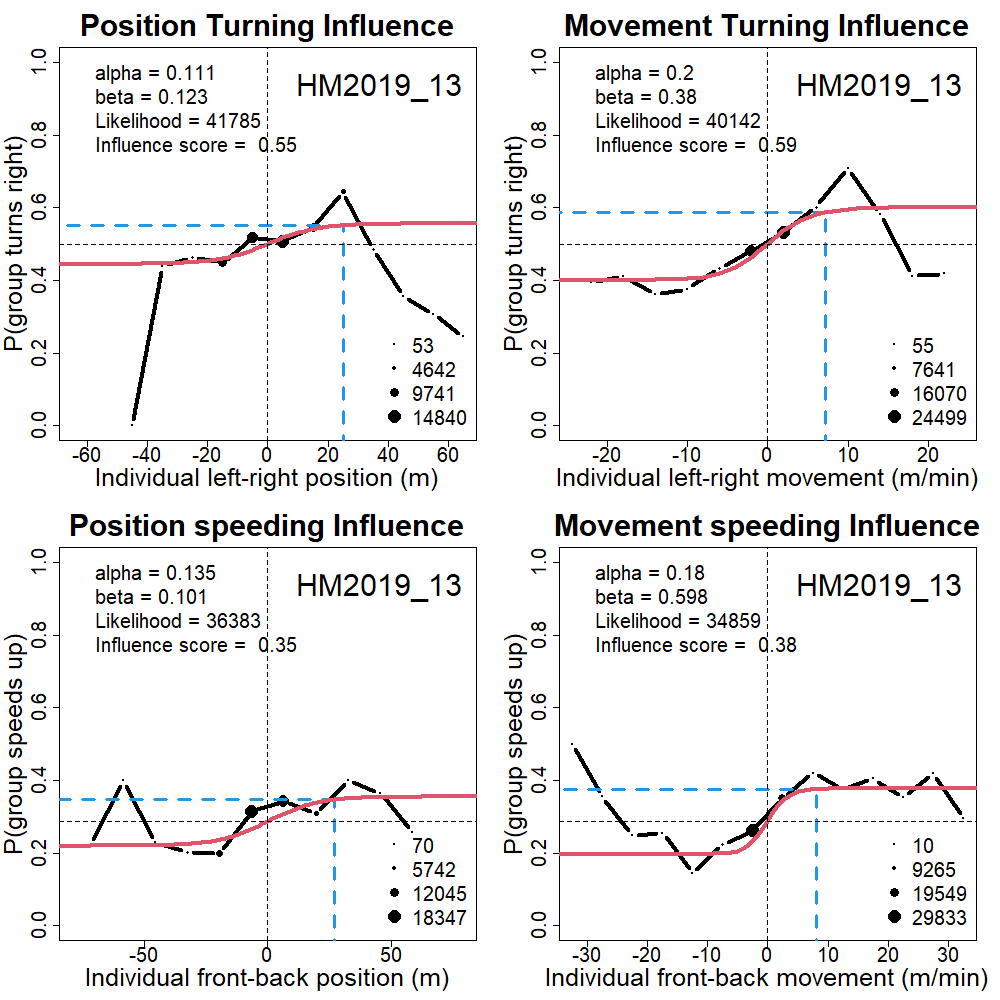

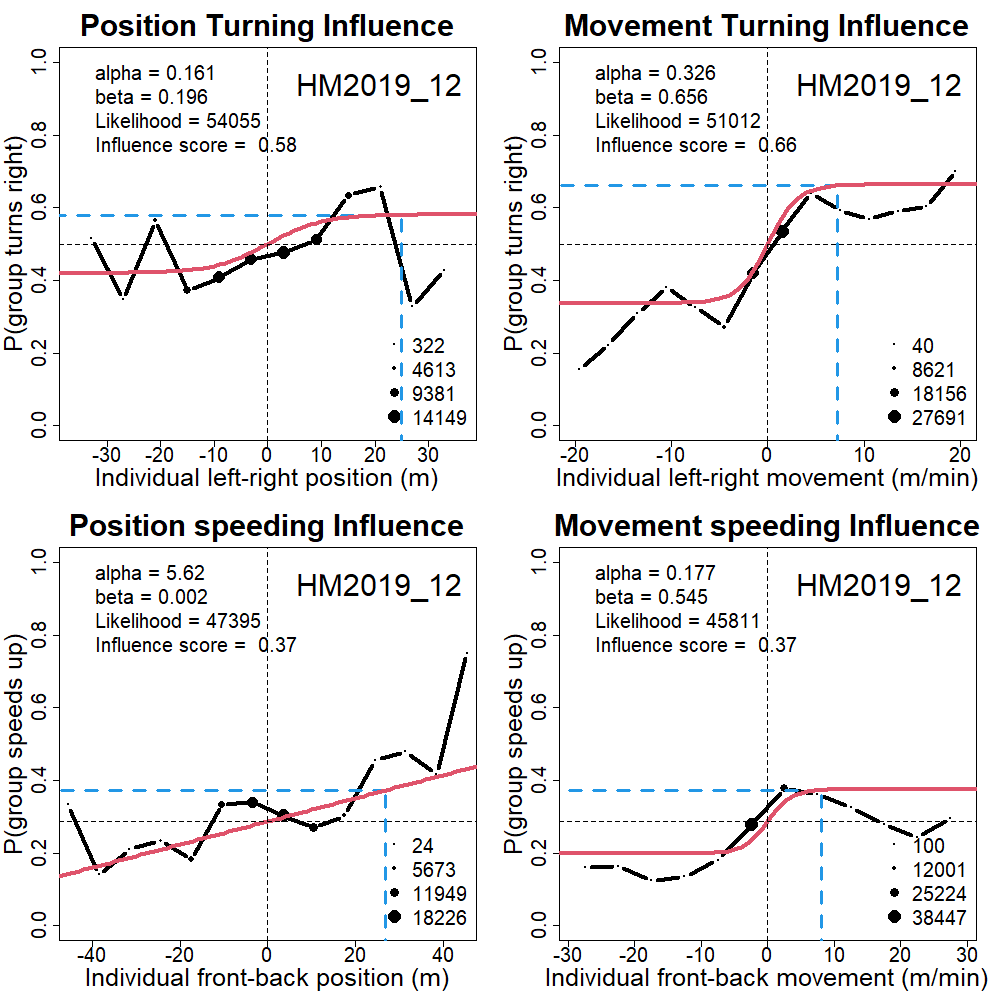

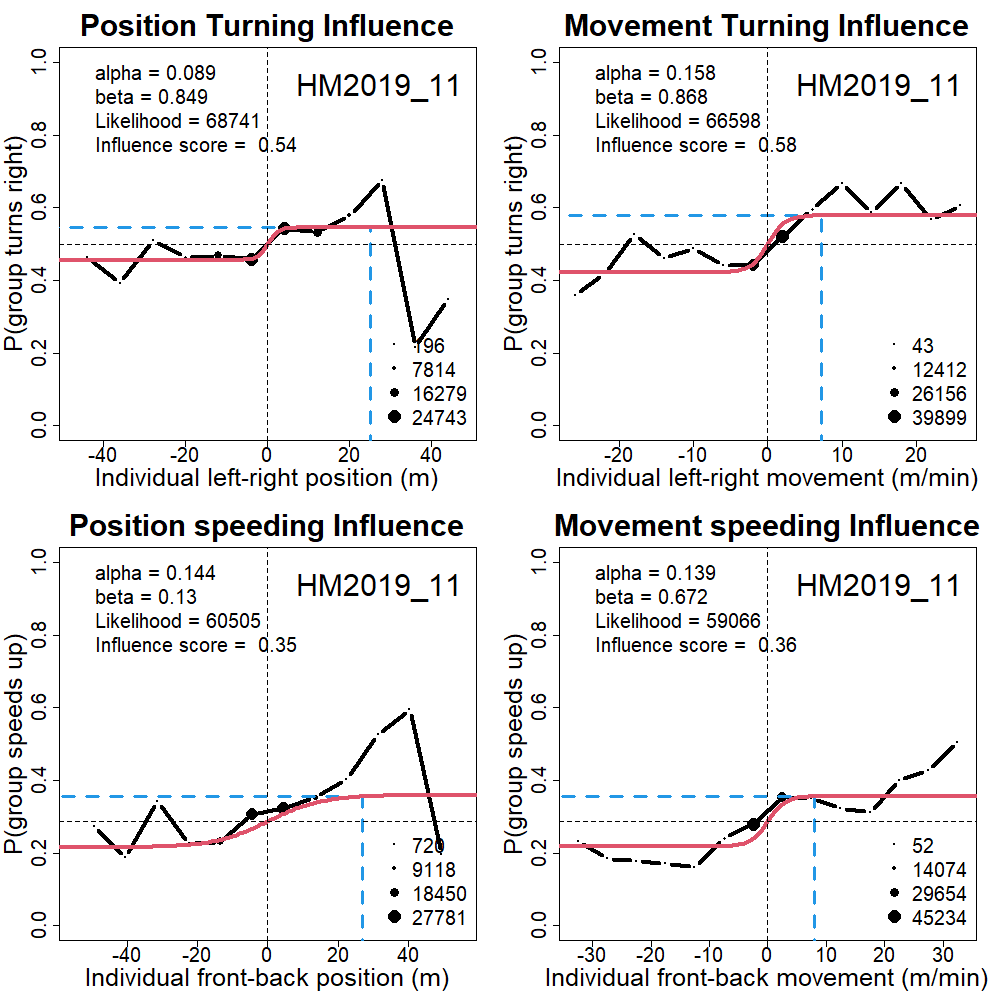

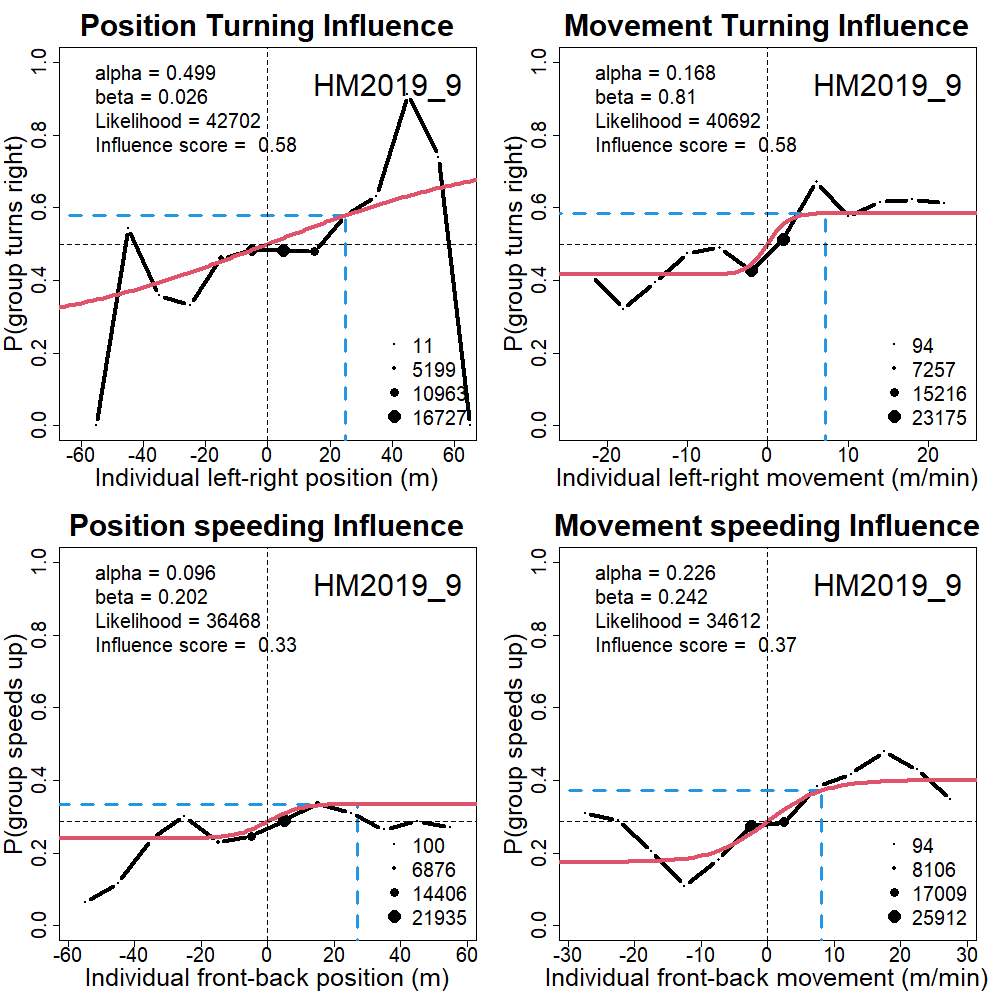

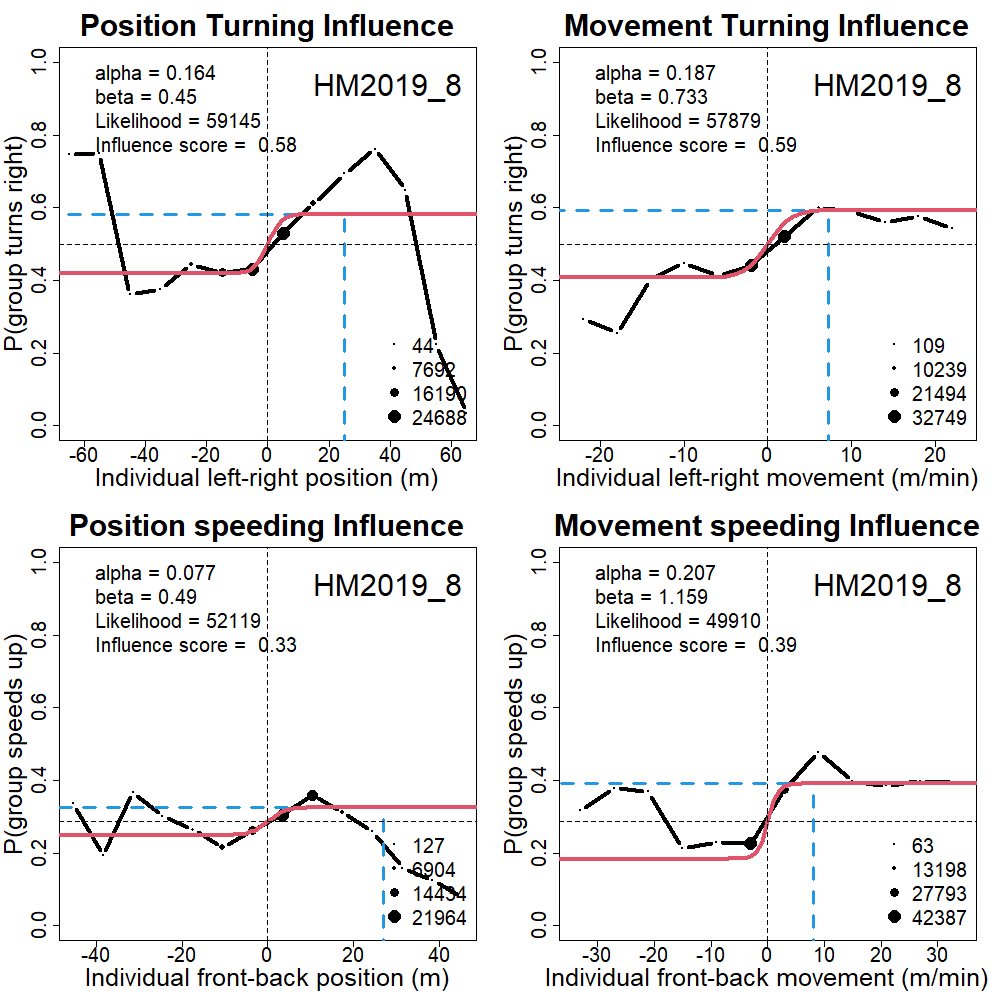

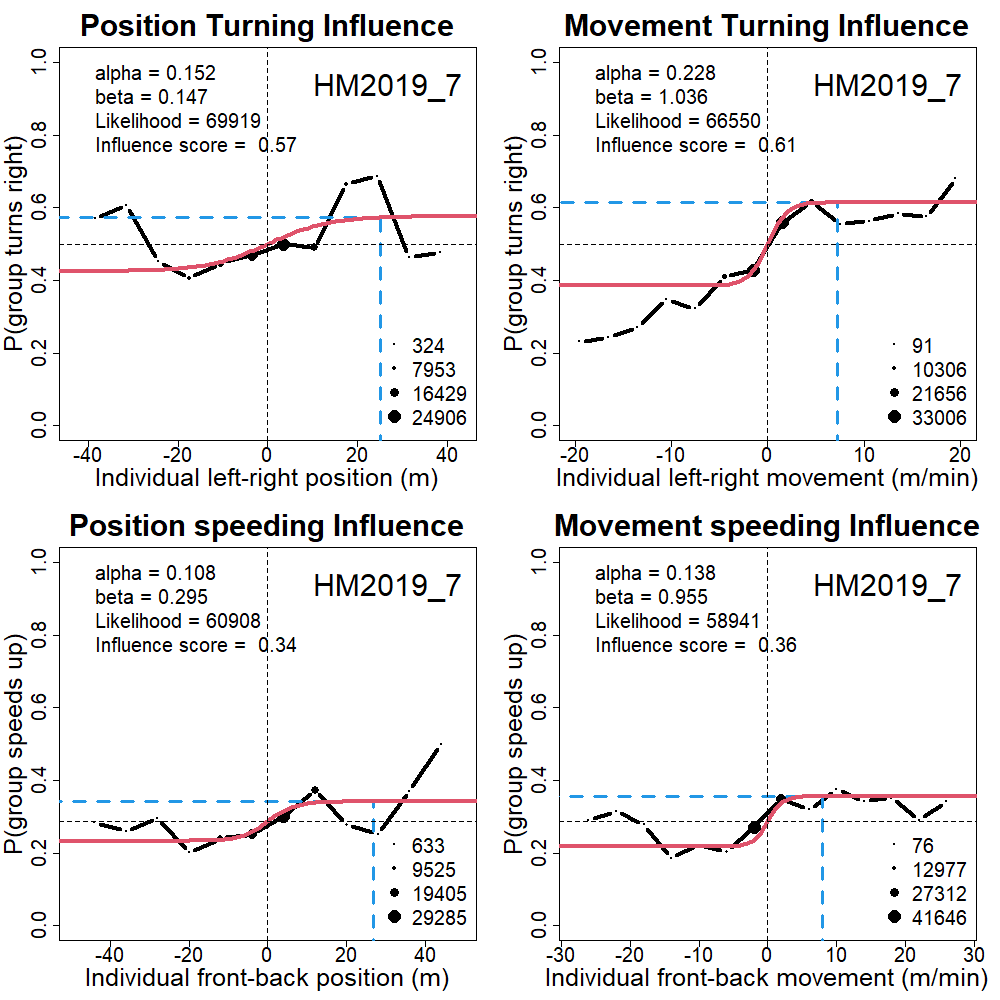
**

**
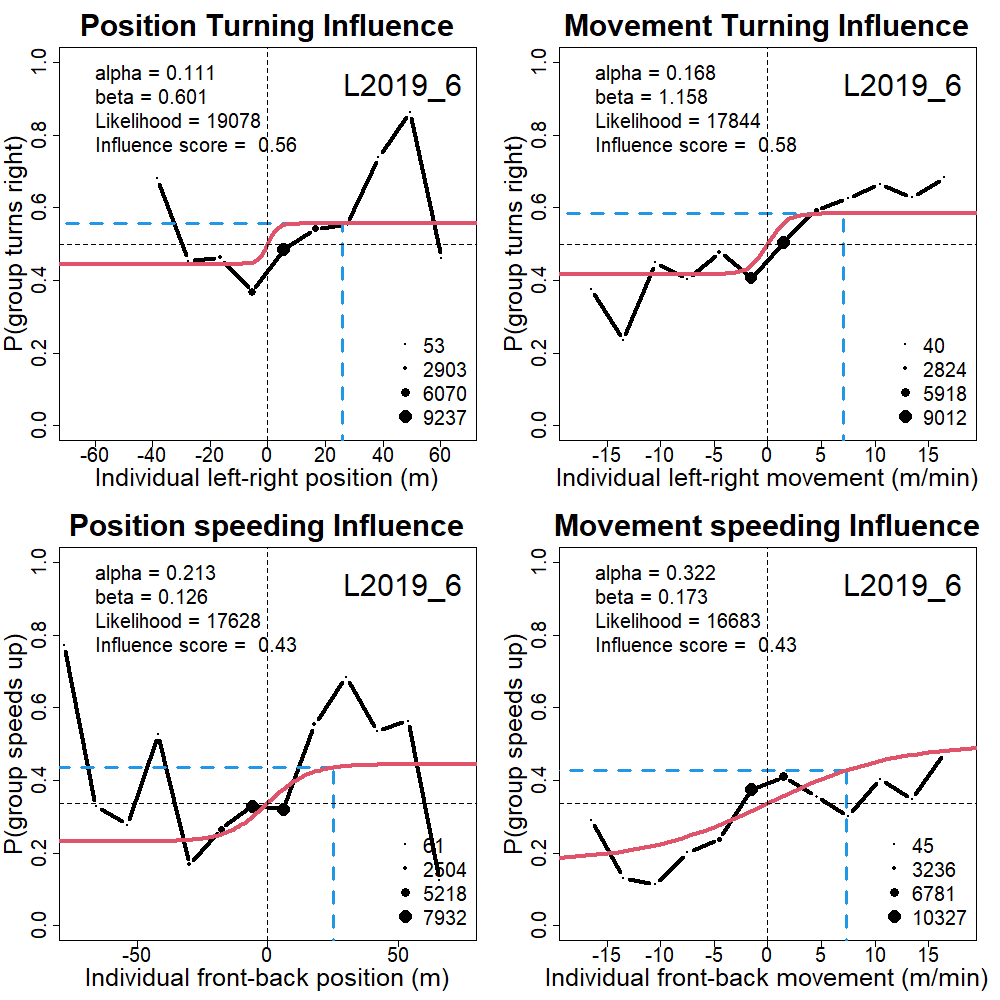

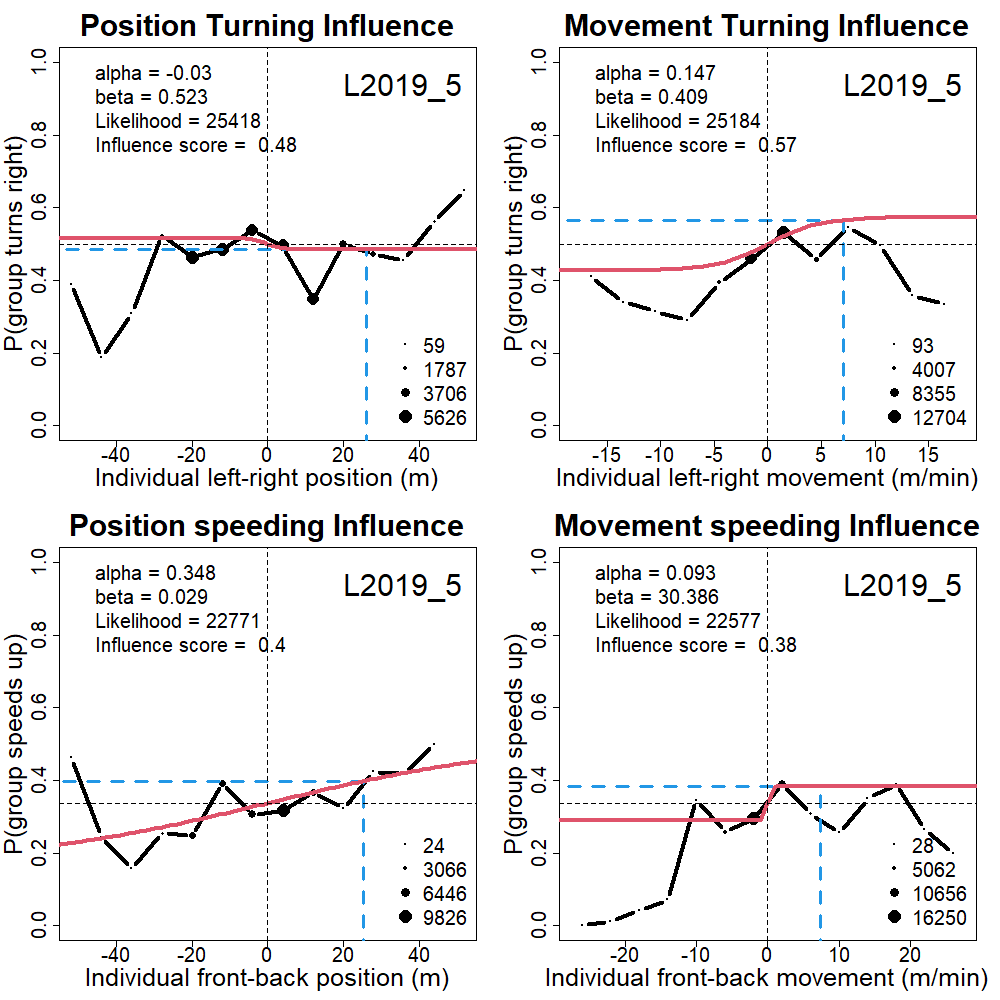

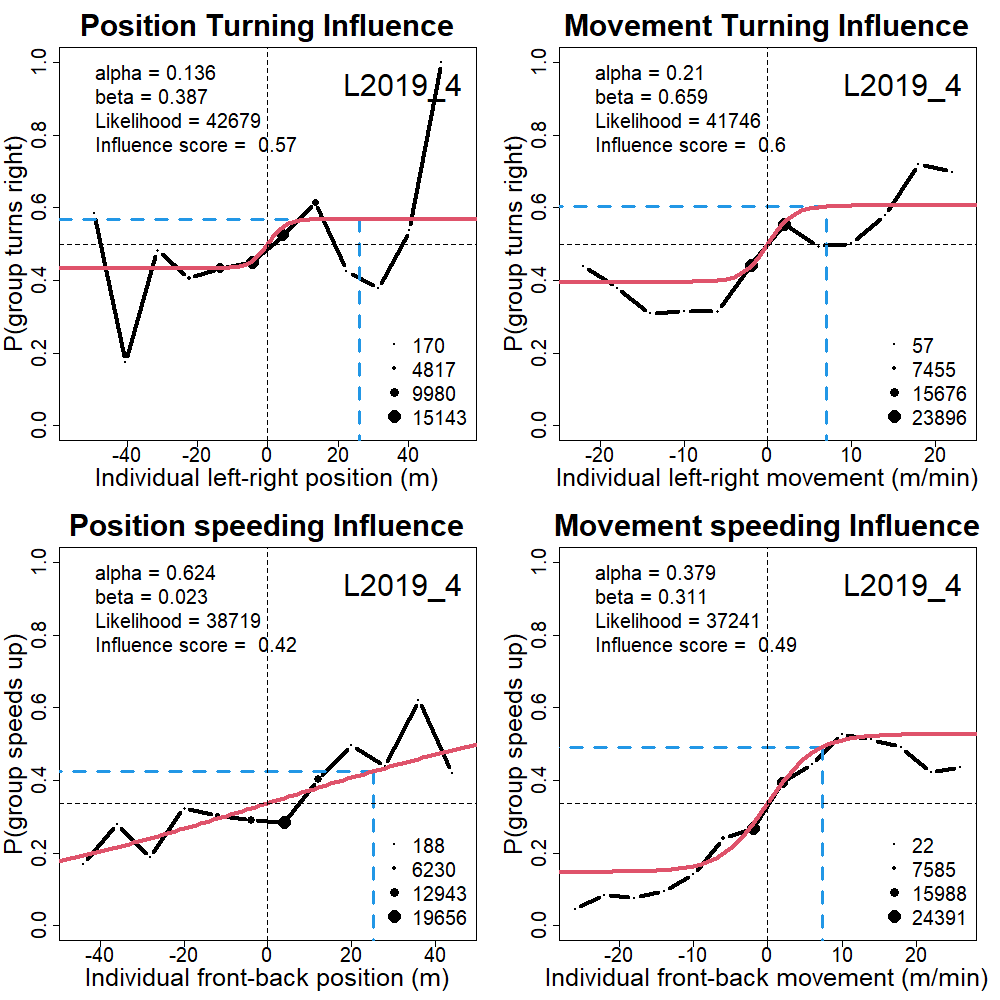

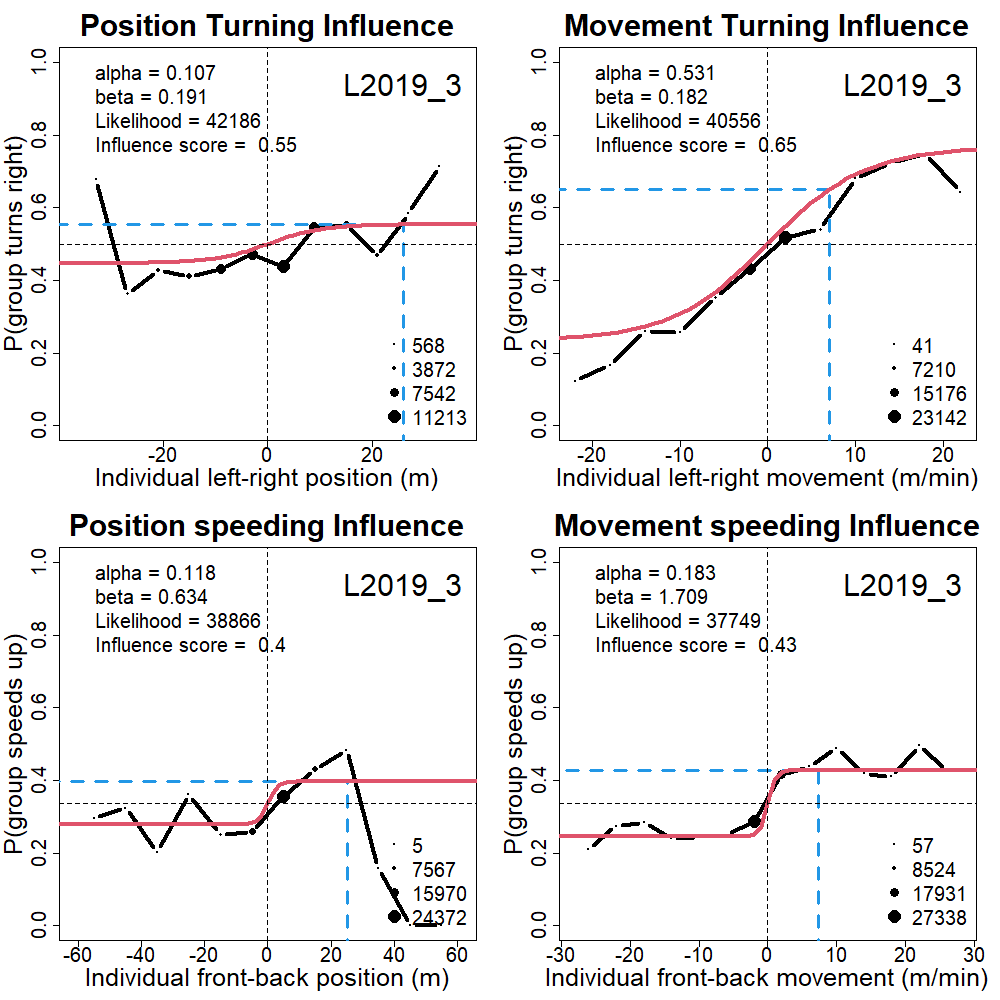

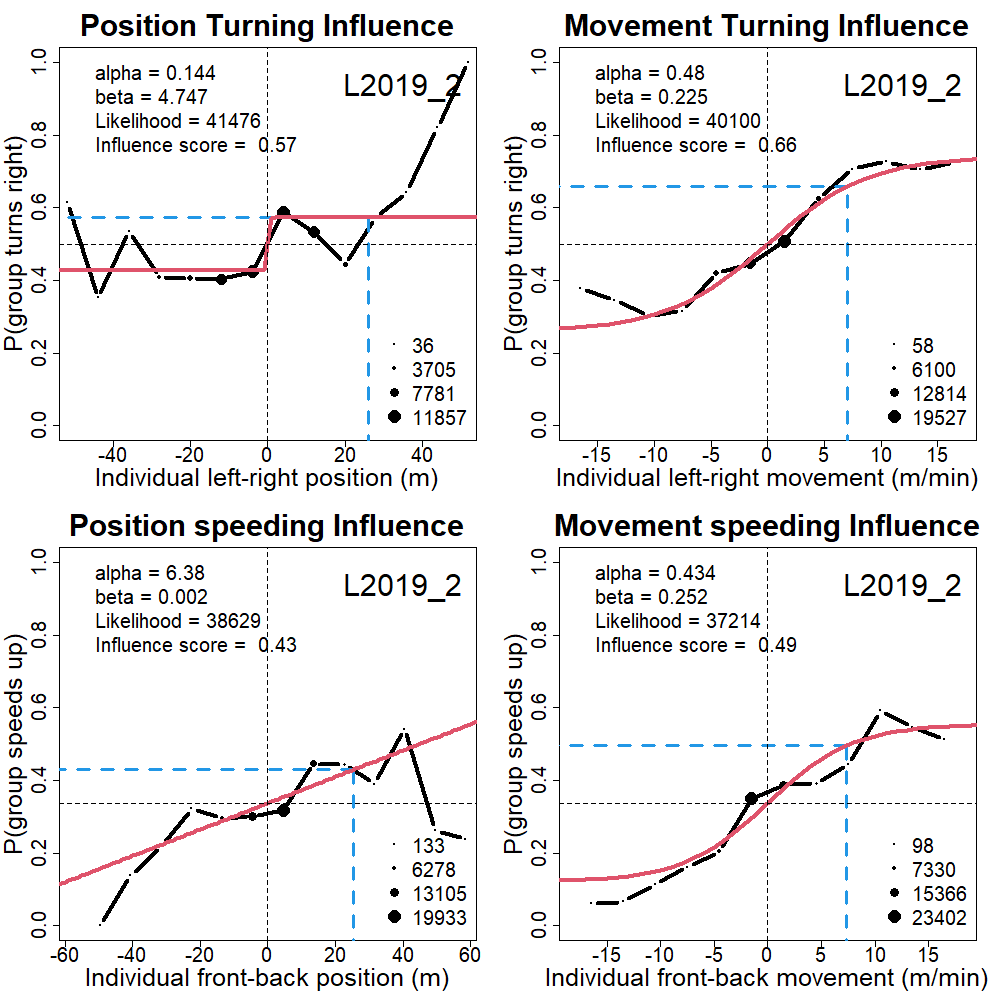

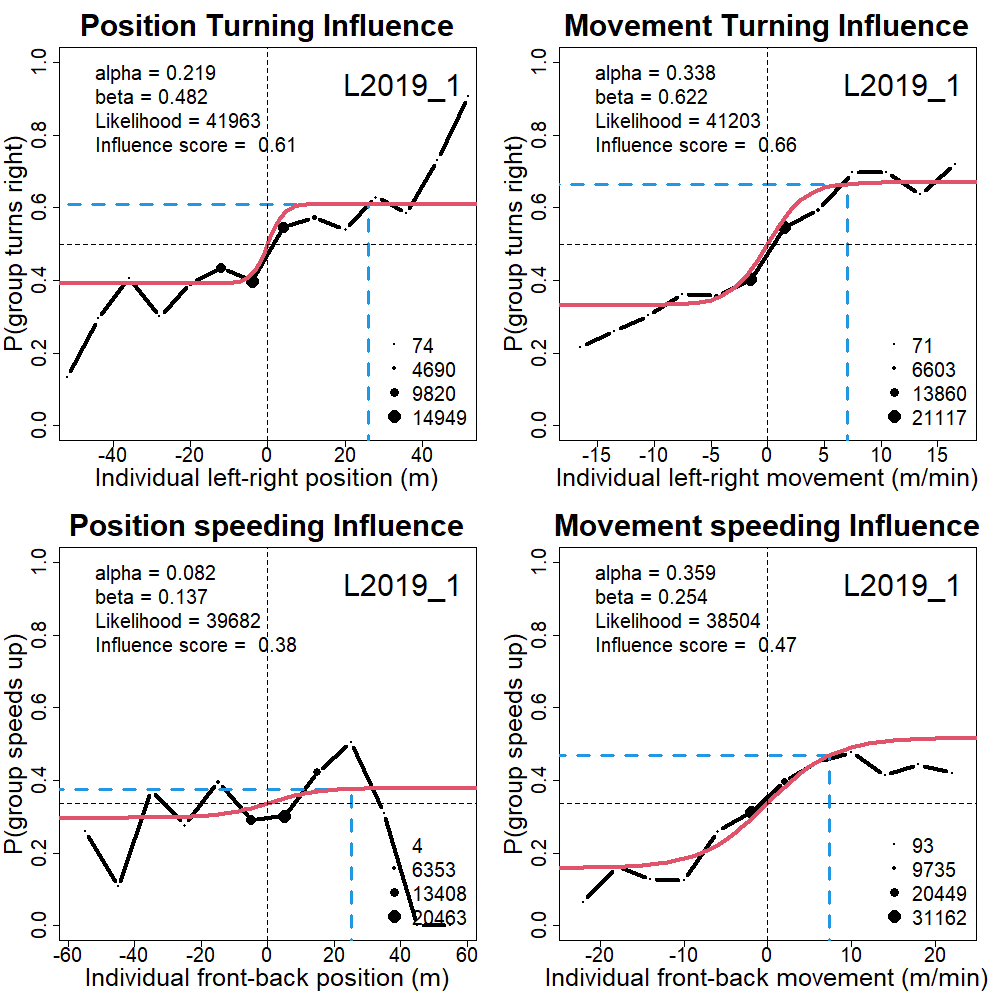
**

**
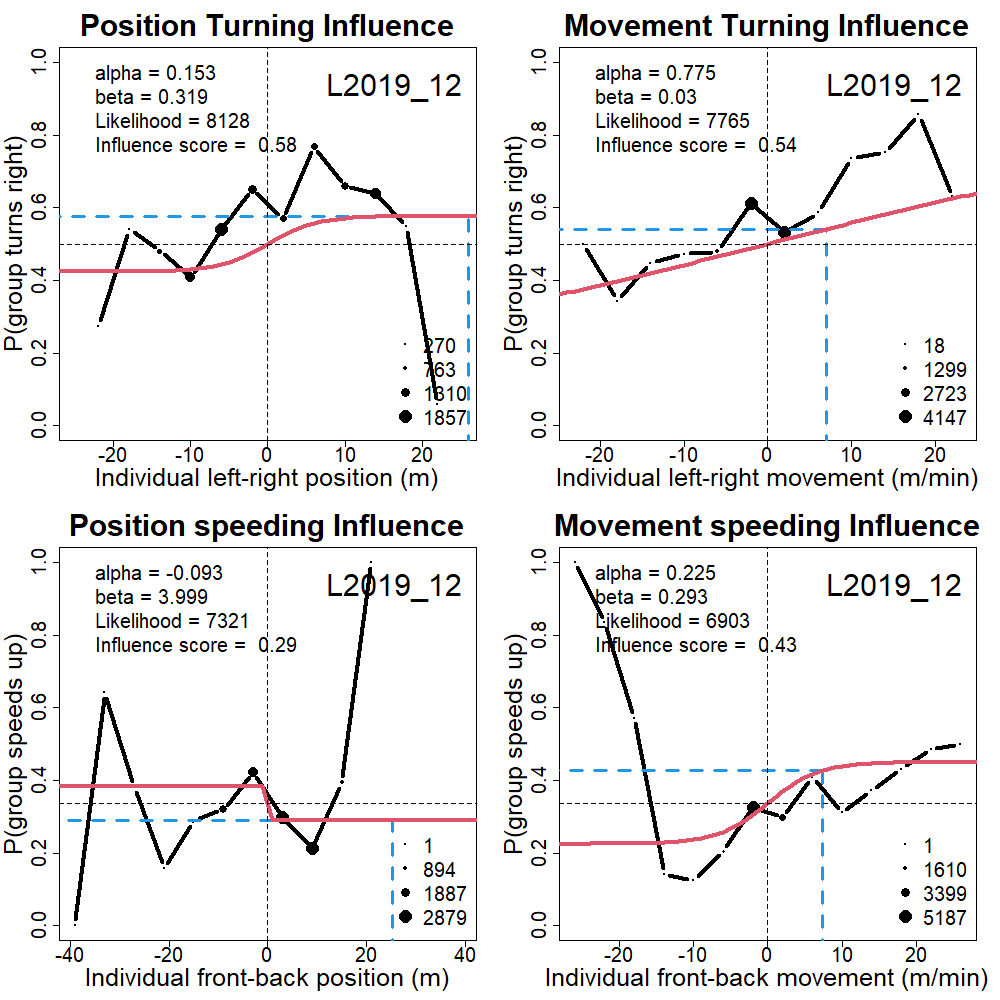

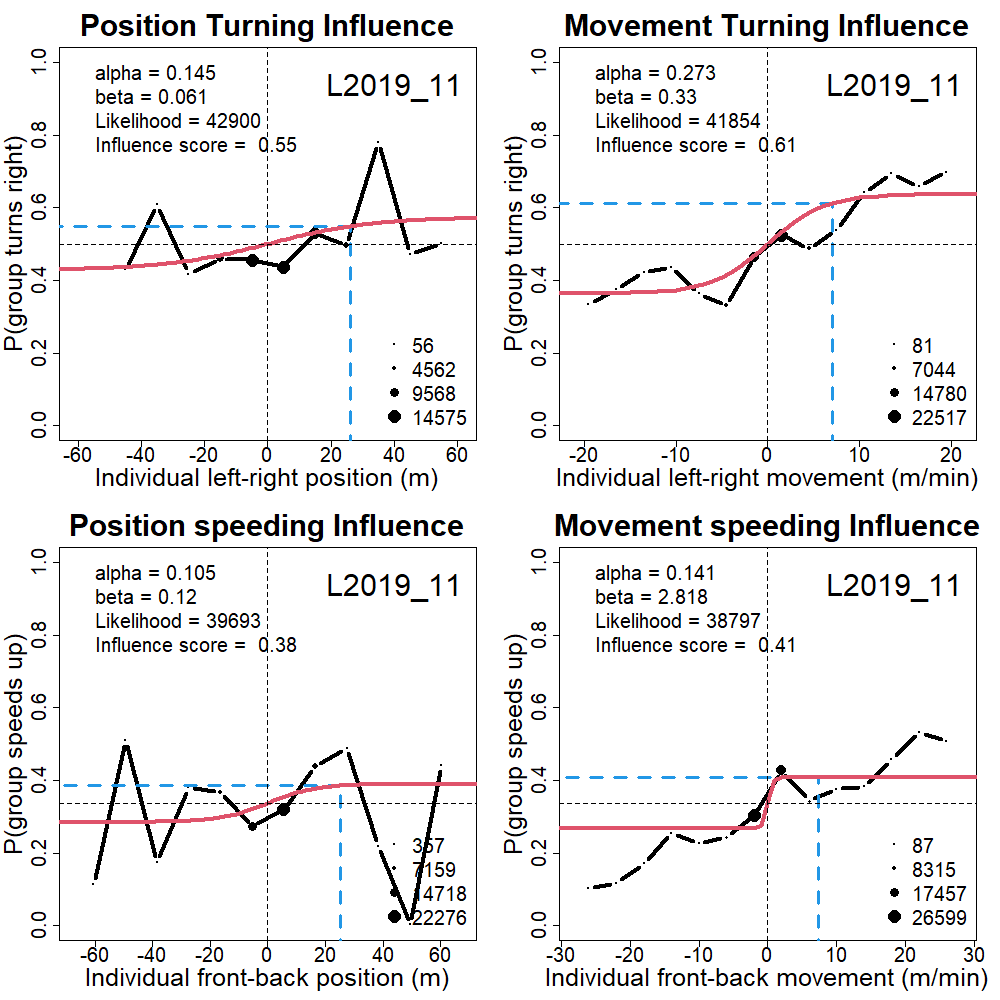

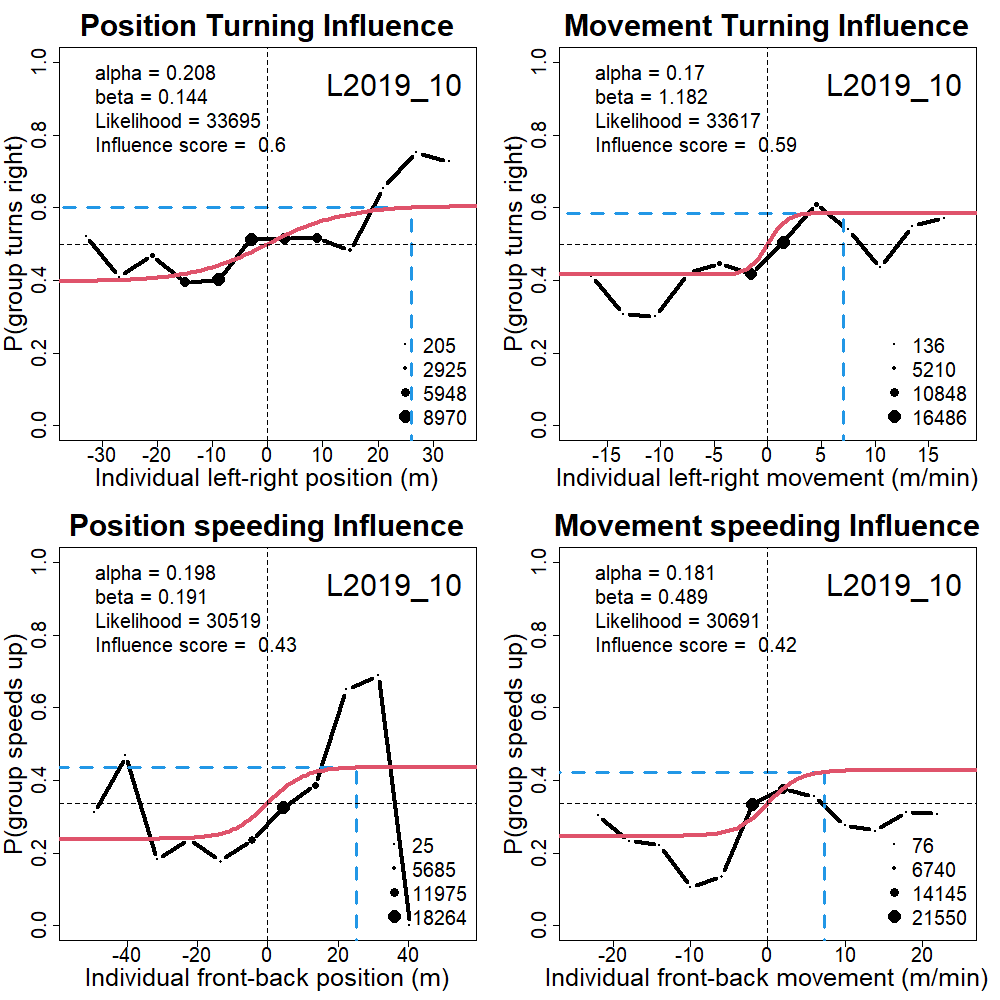

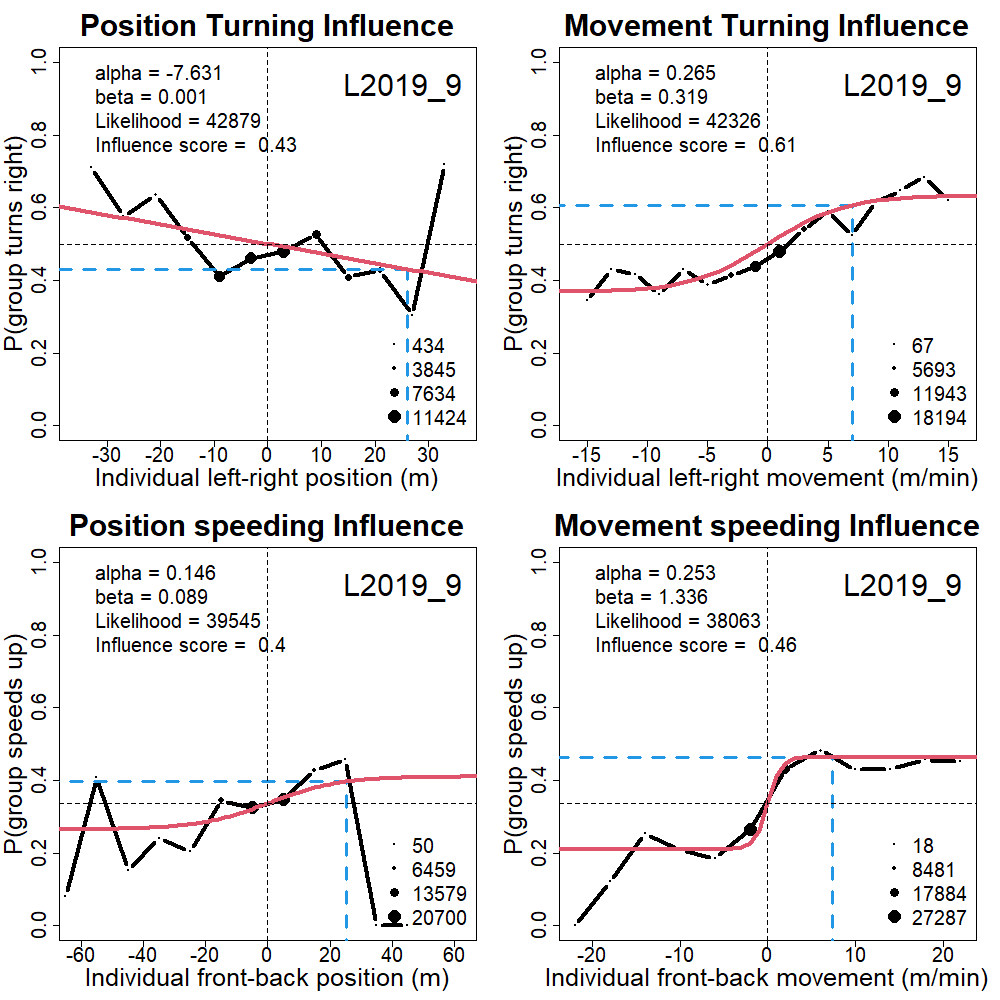

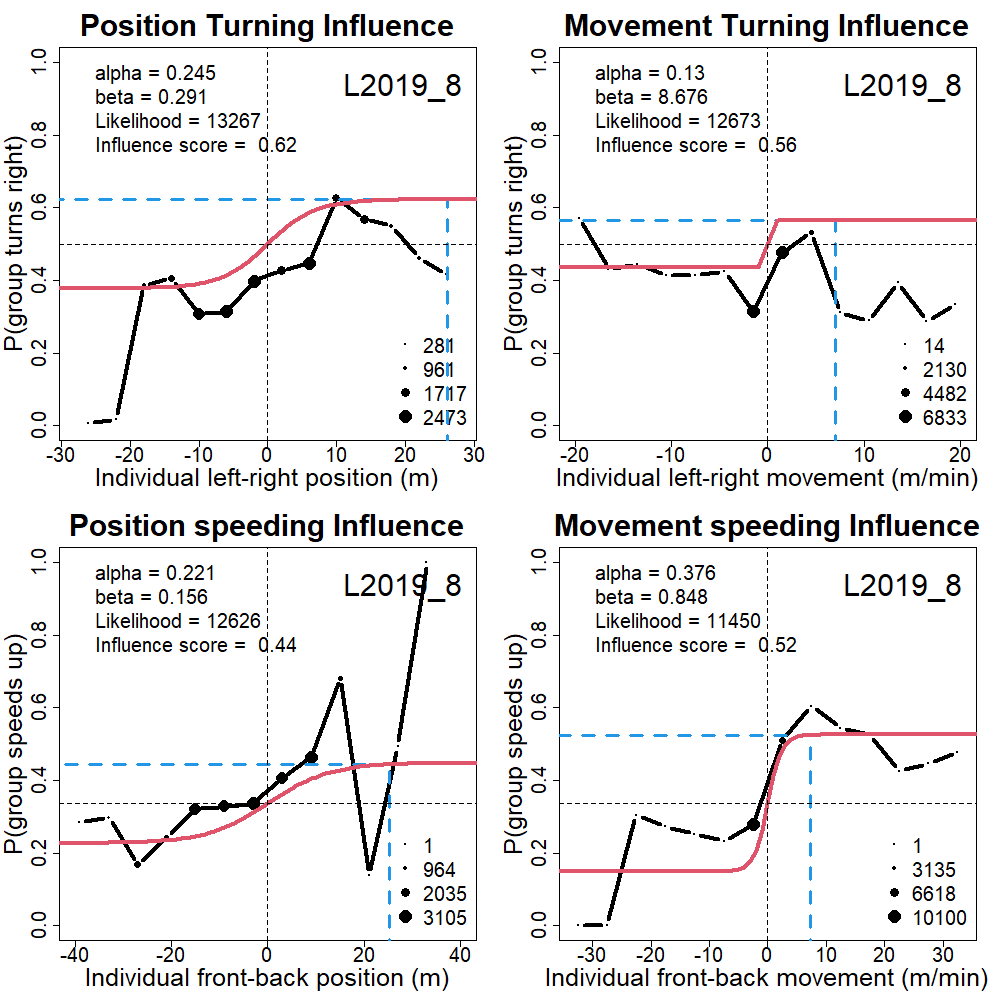

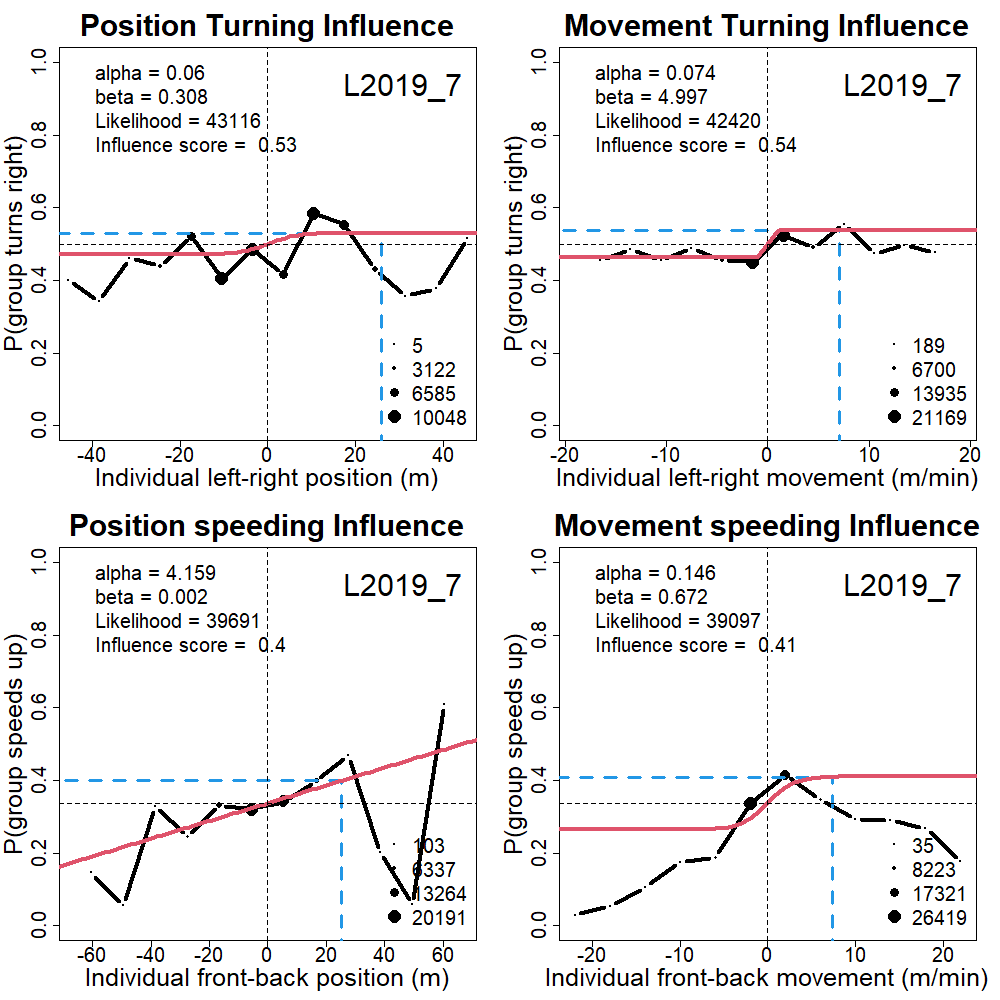
**

**
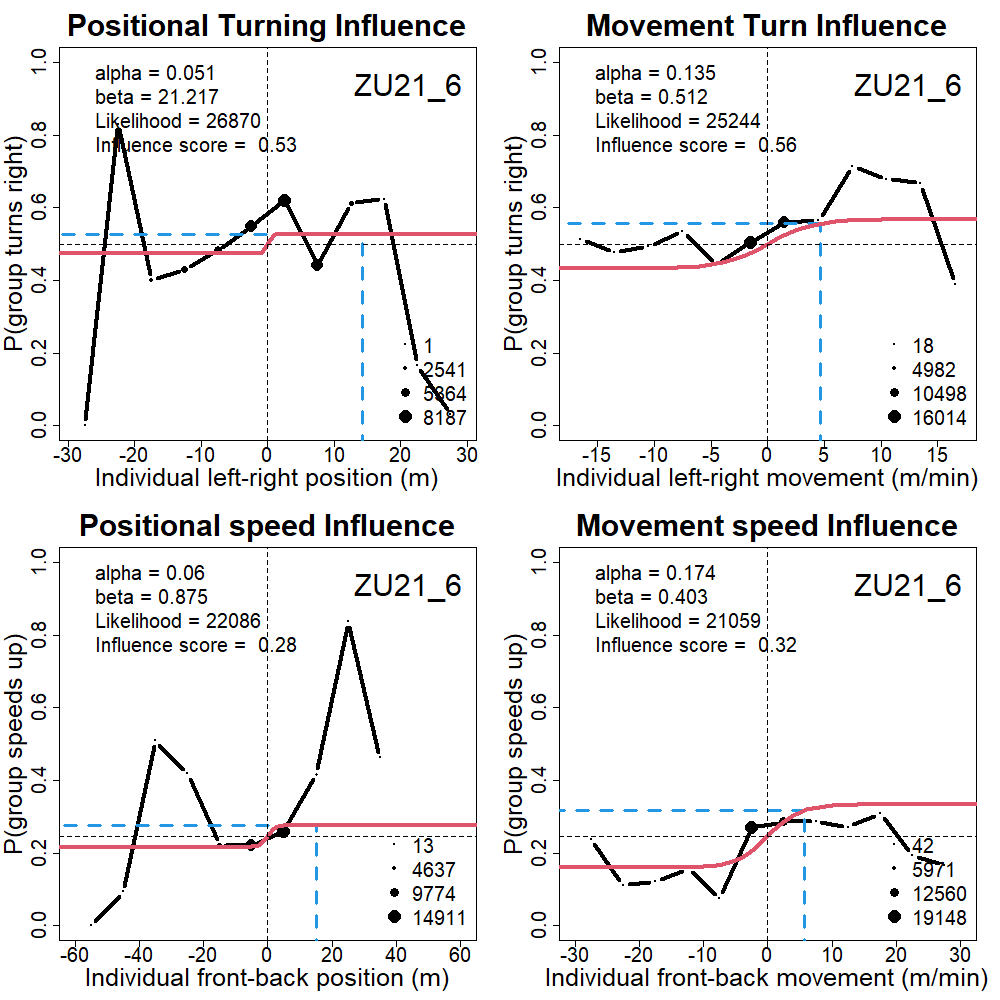

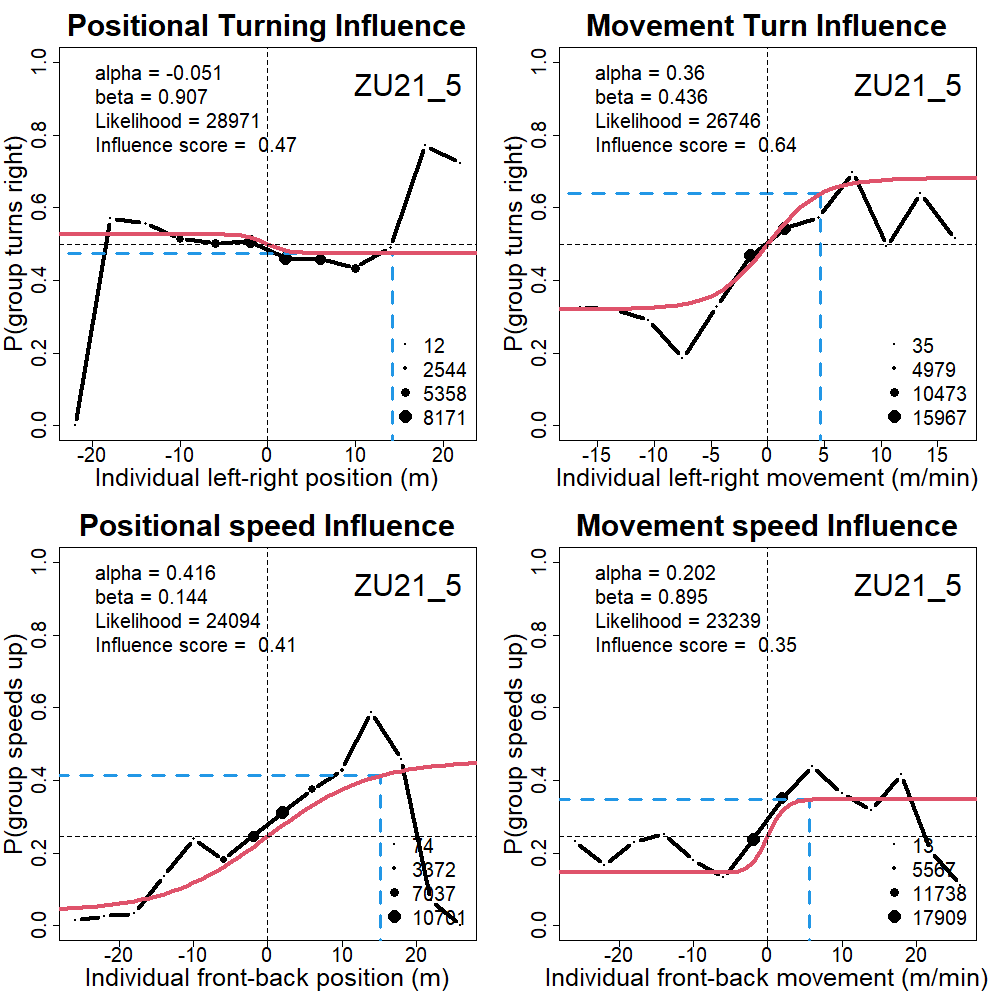

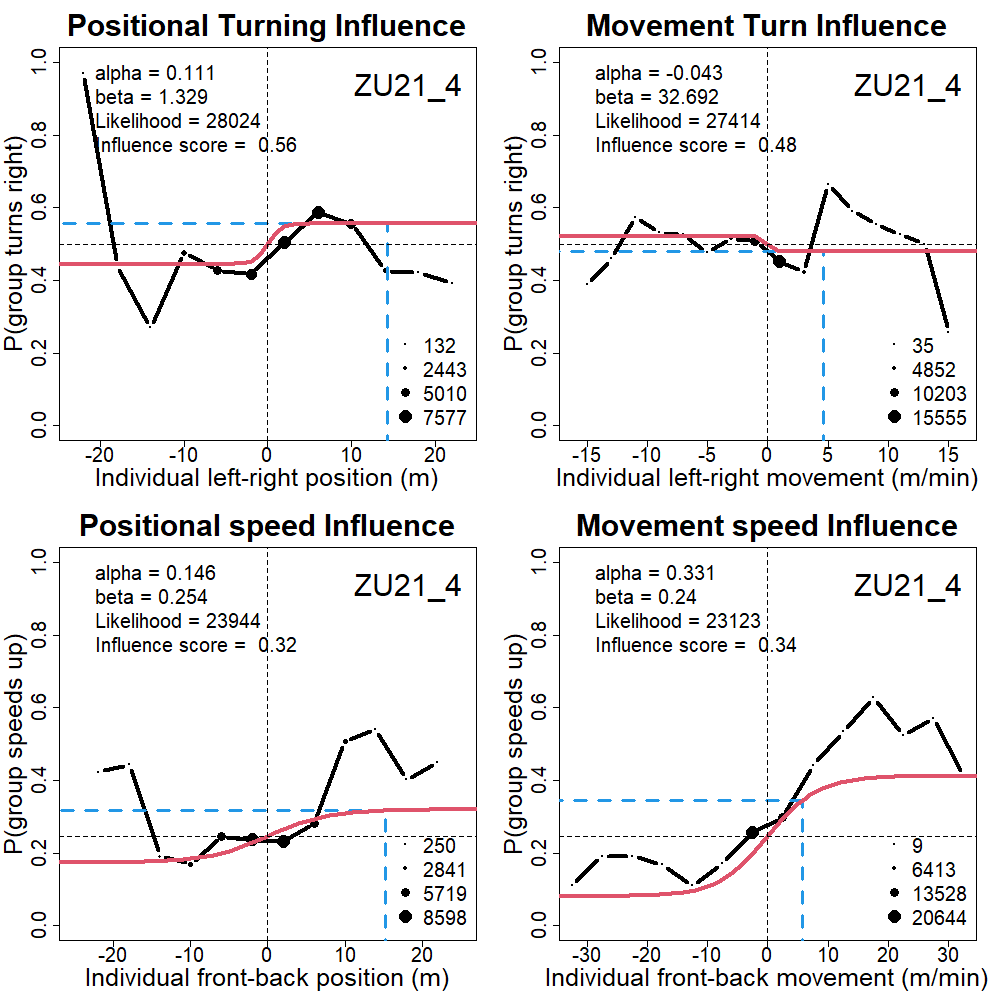

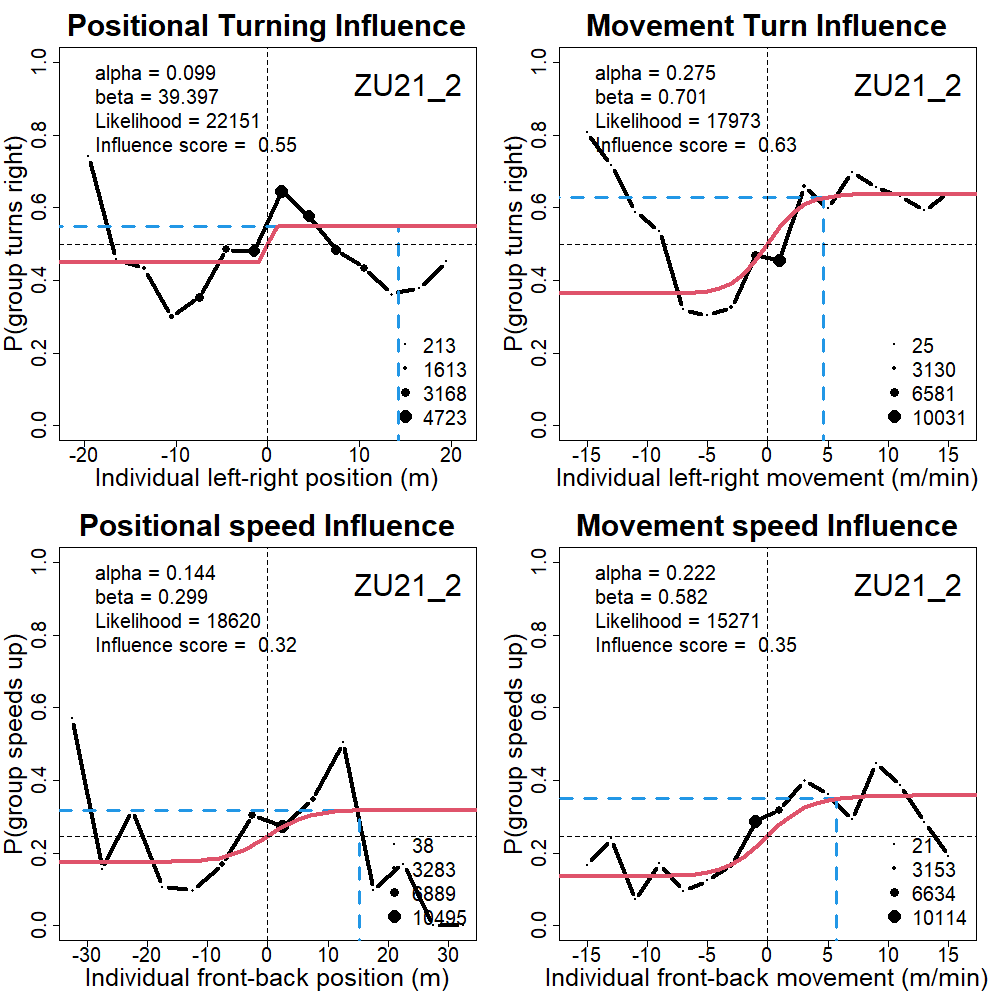

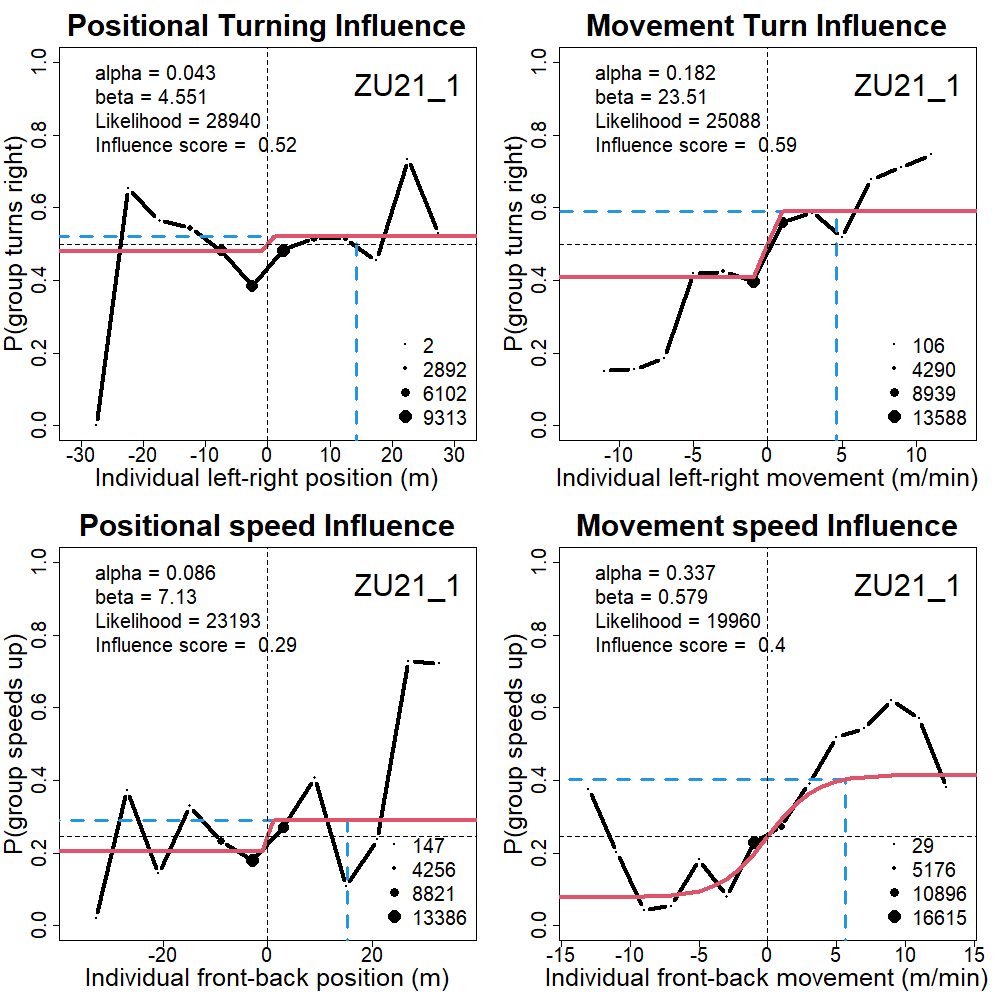

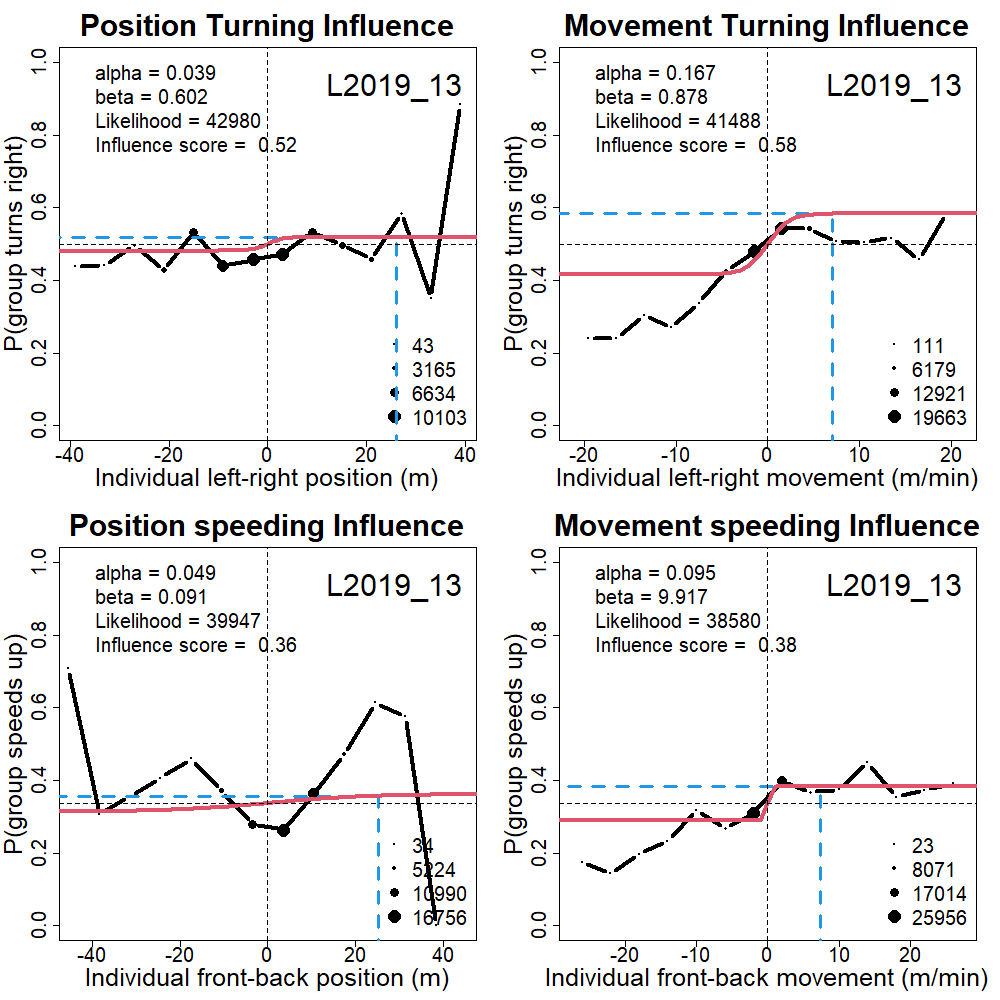
**

**
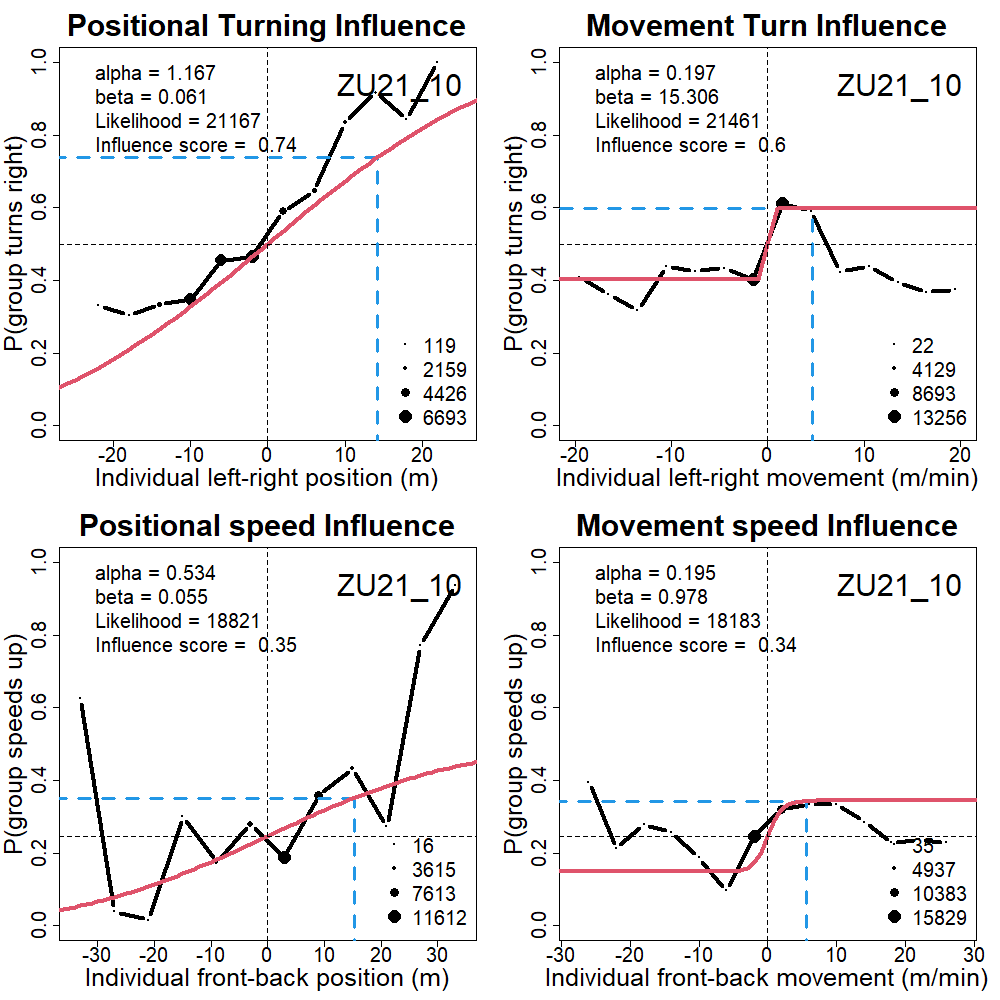

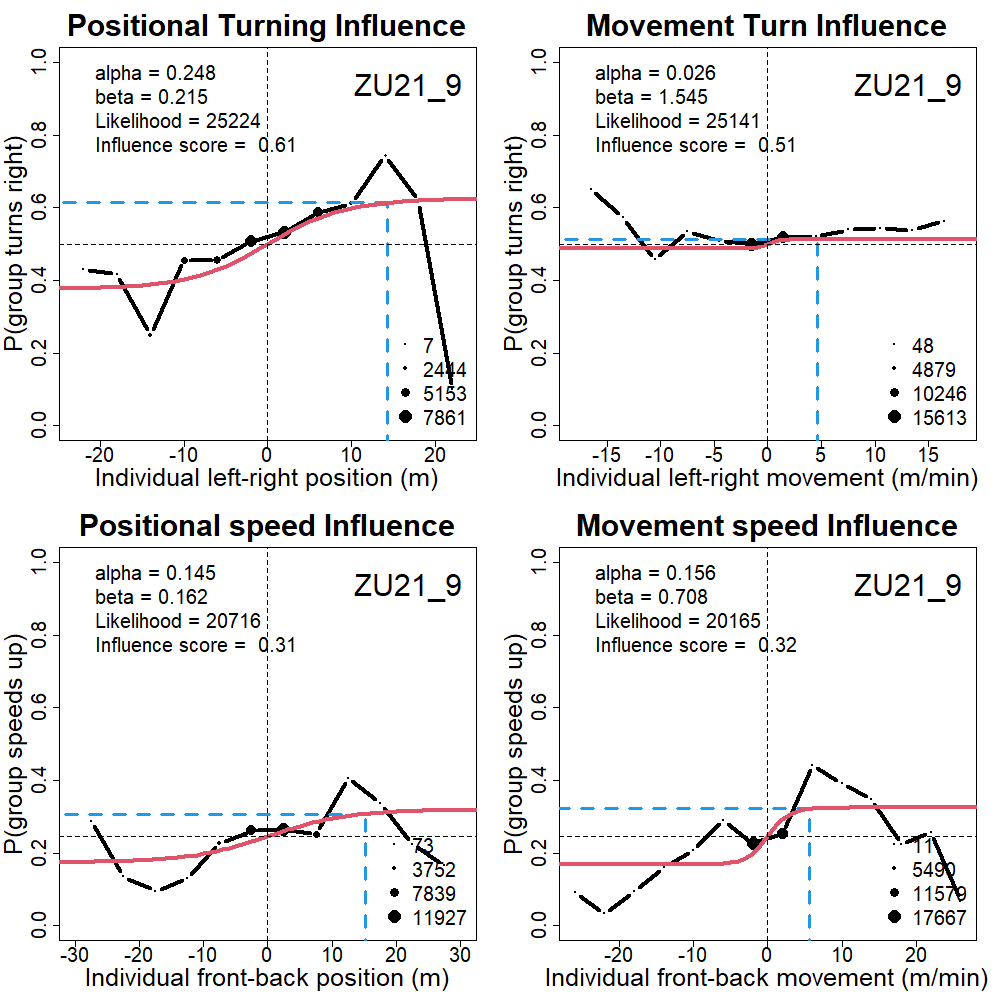

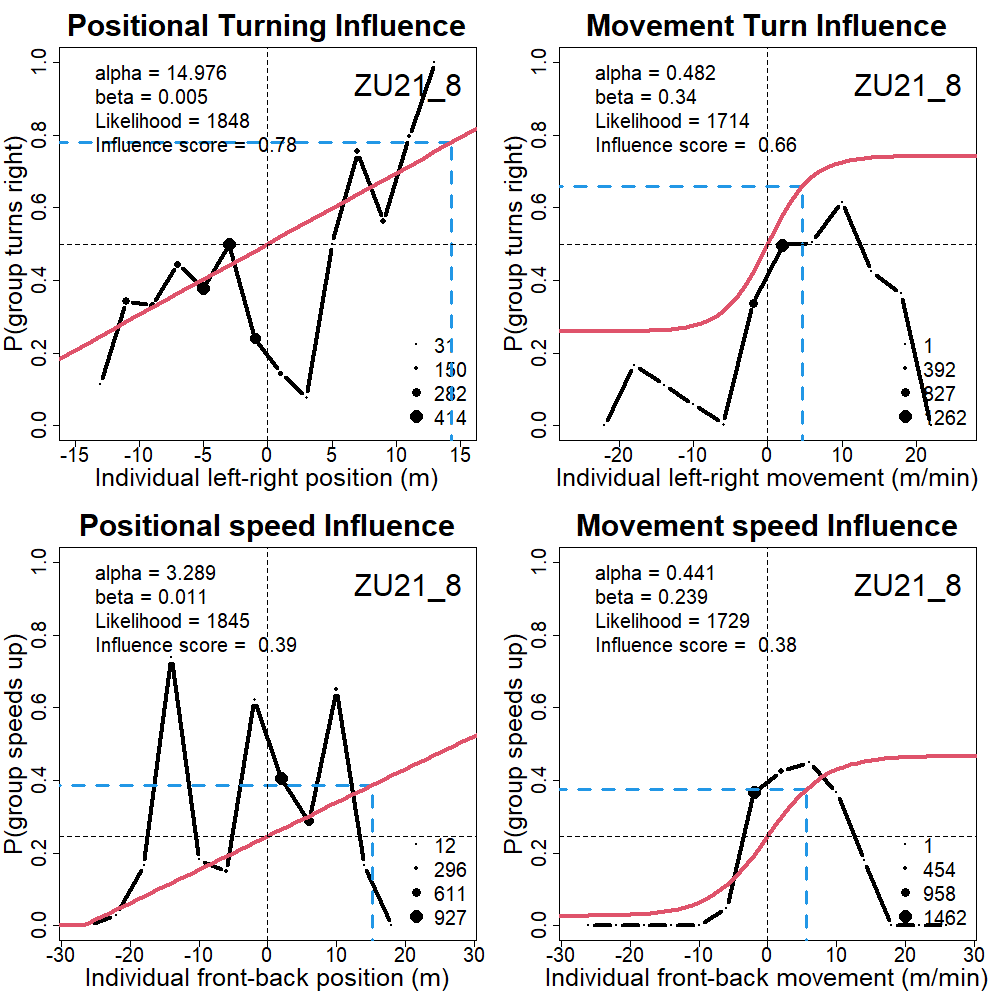

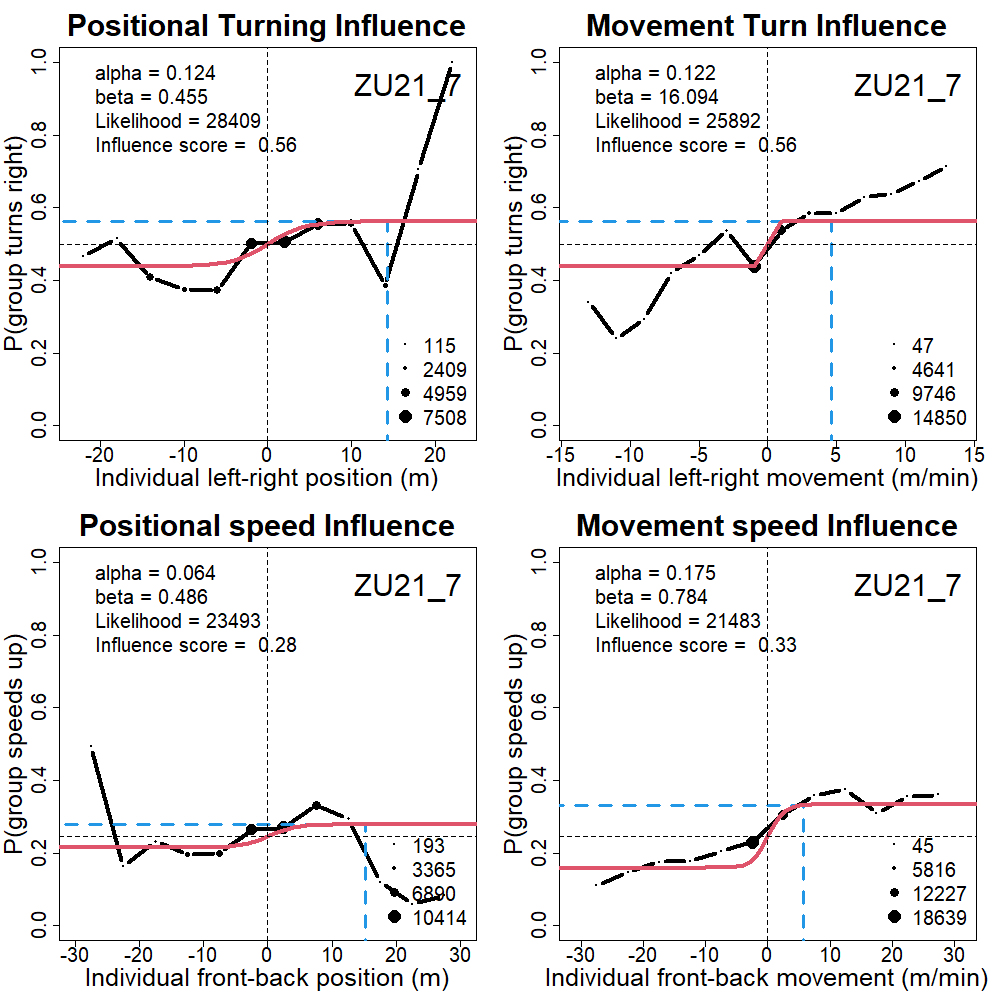

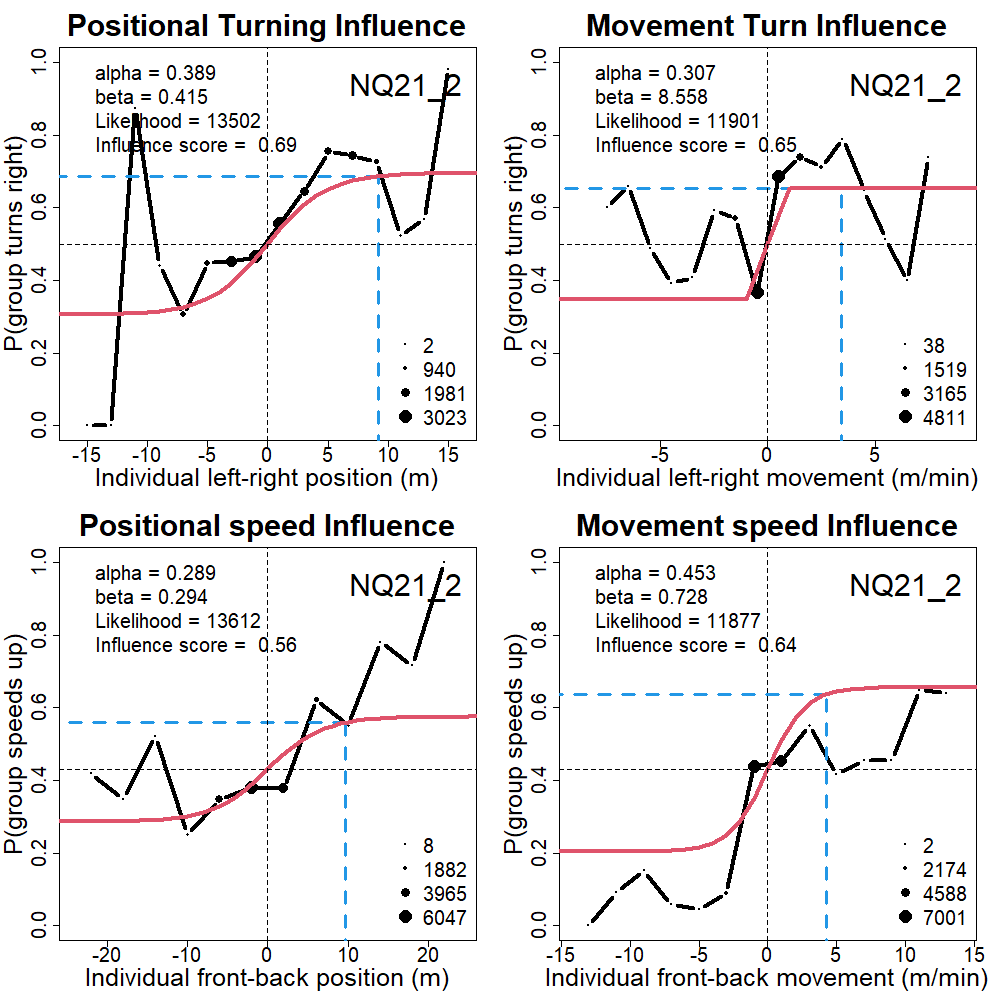

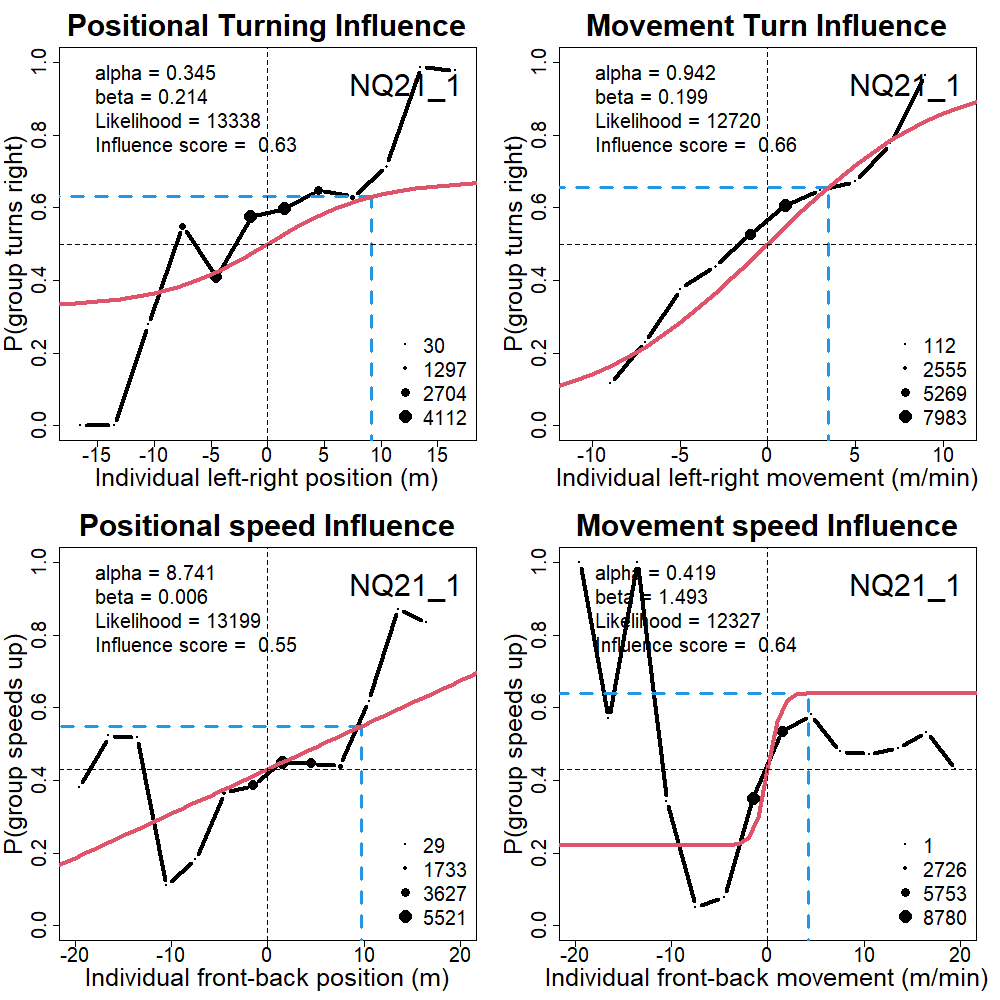
**

**
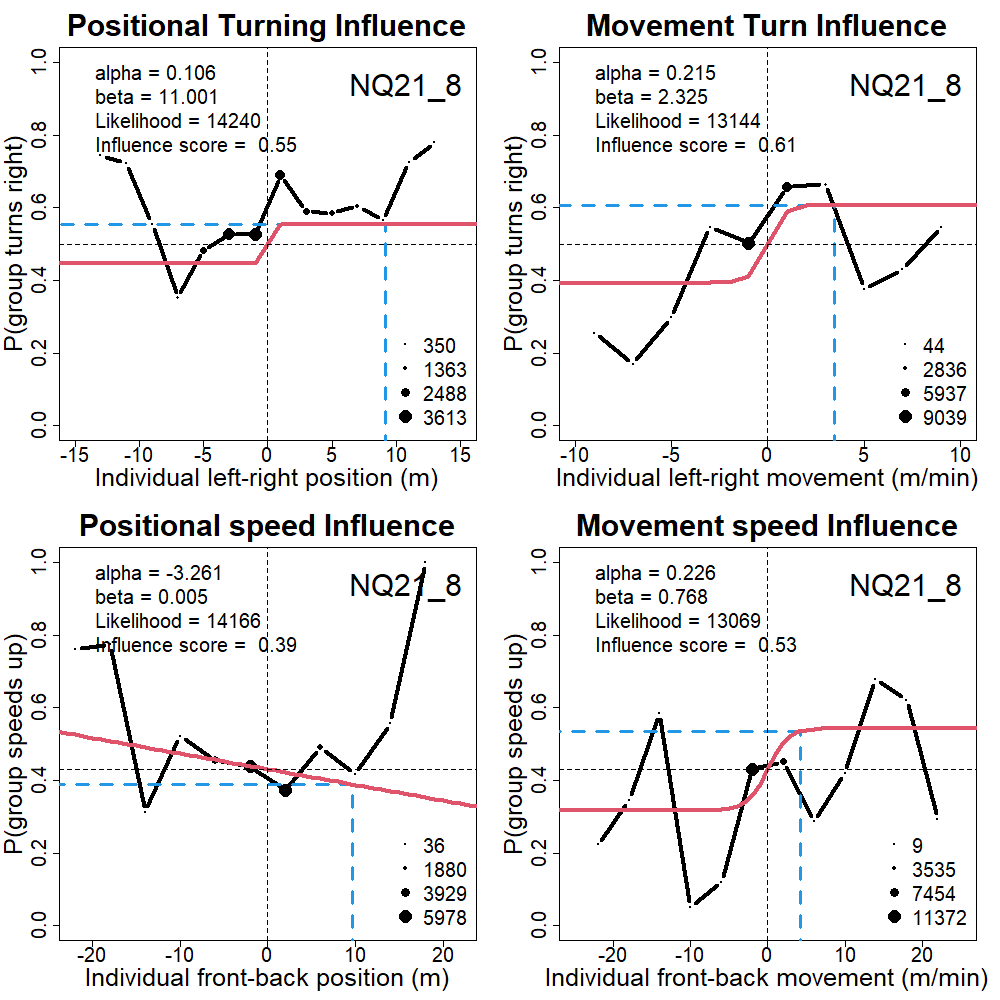

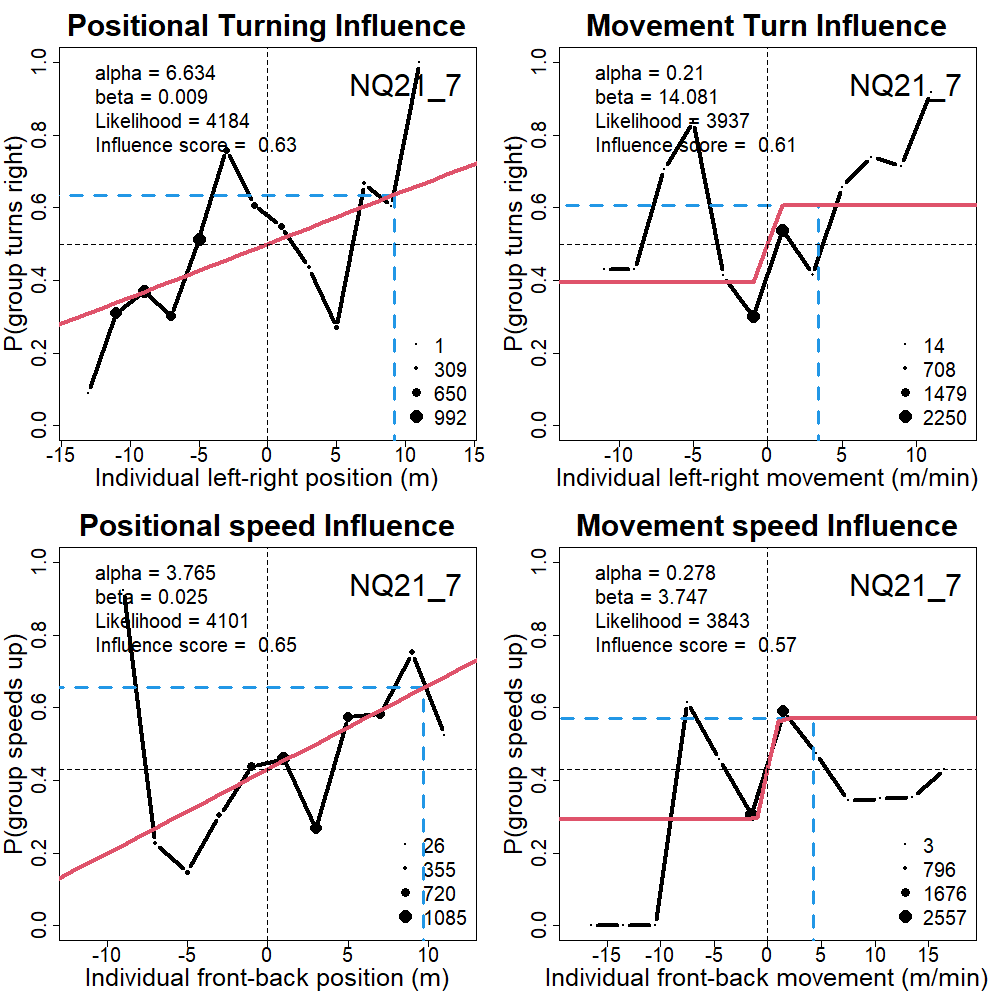

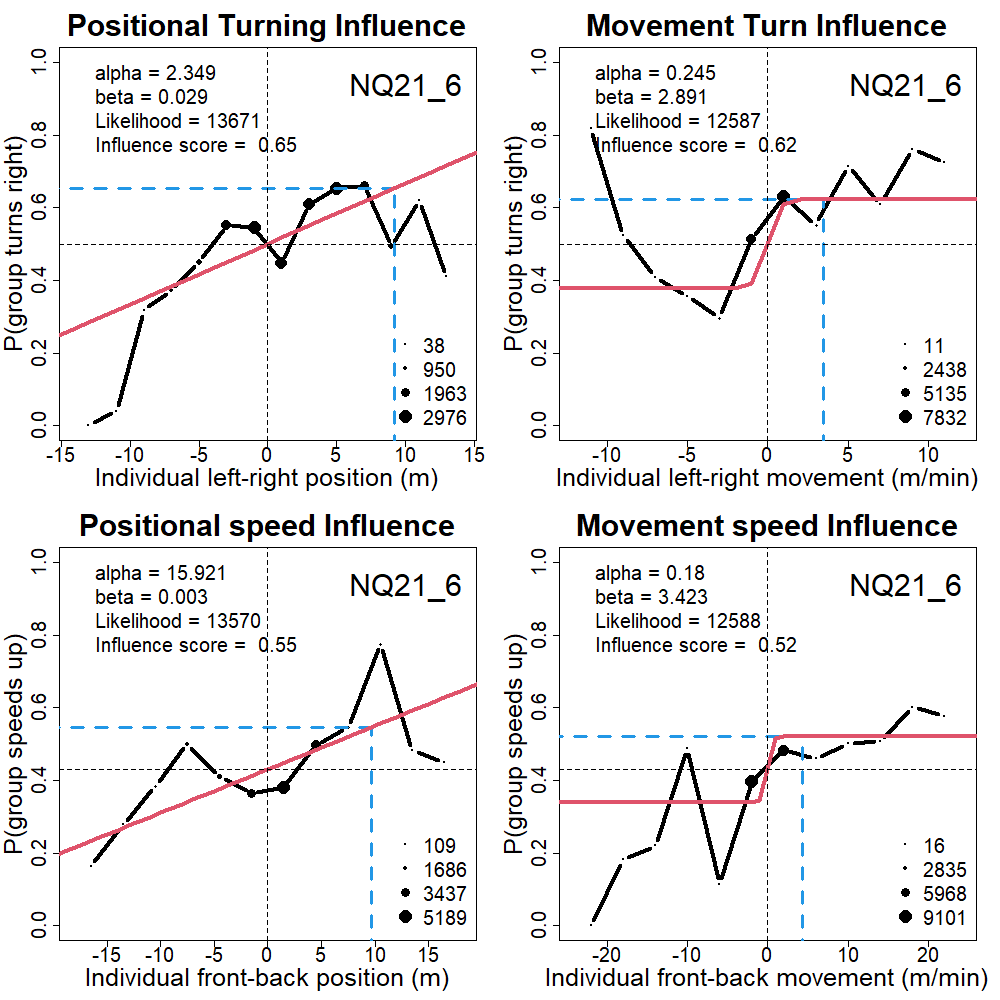

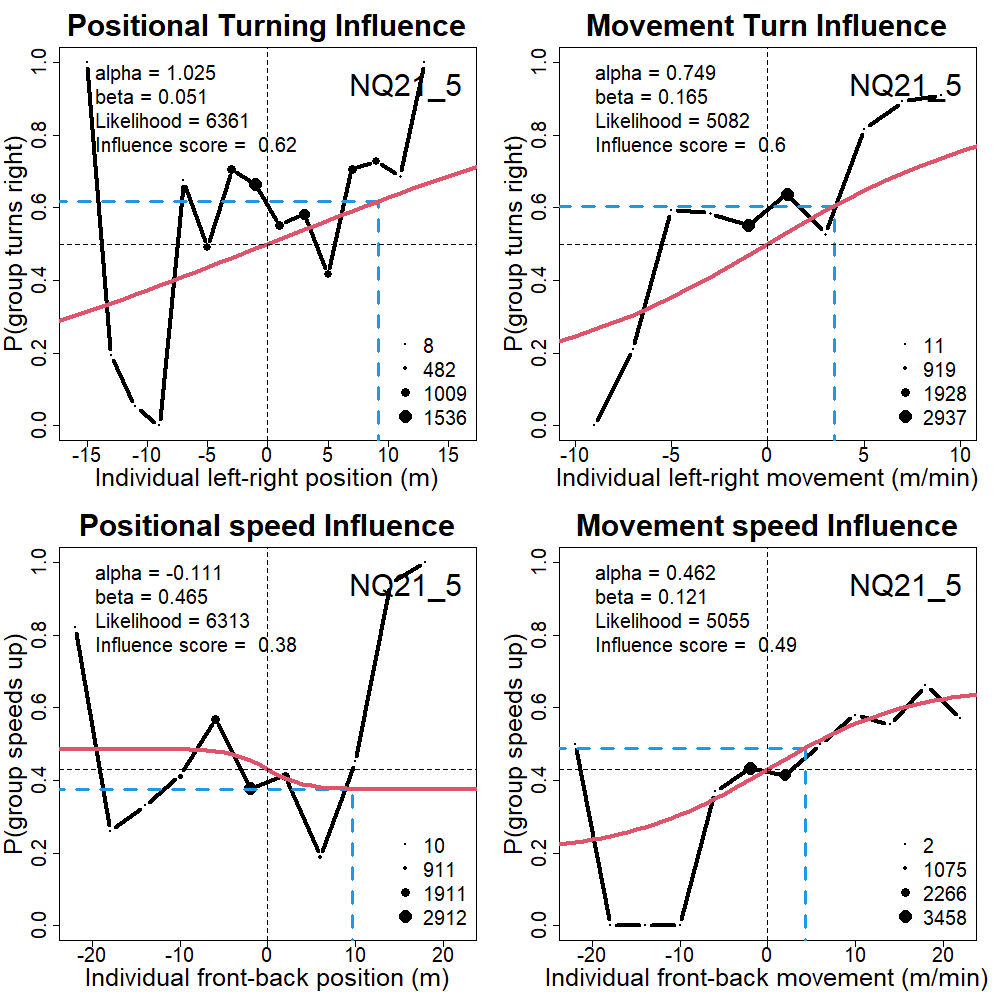

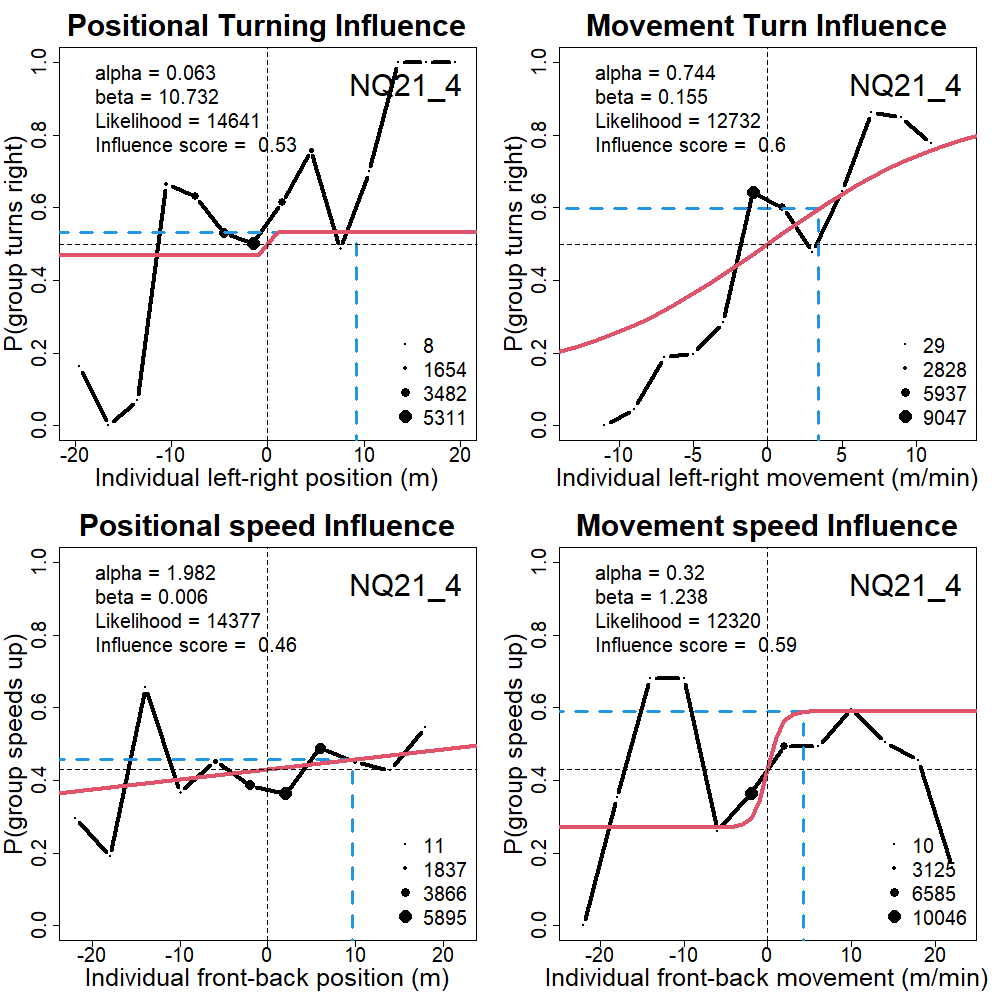

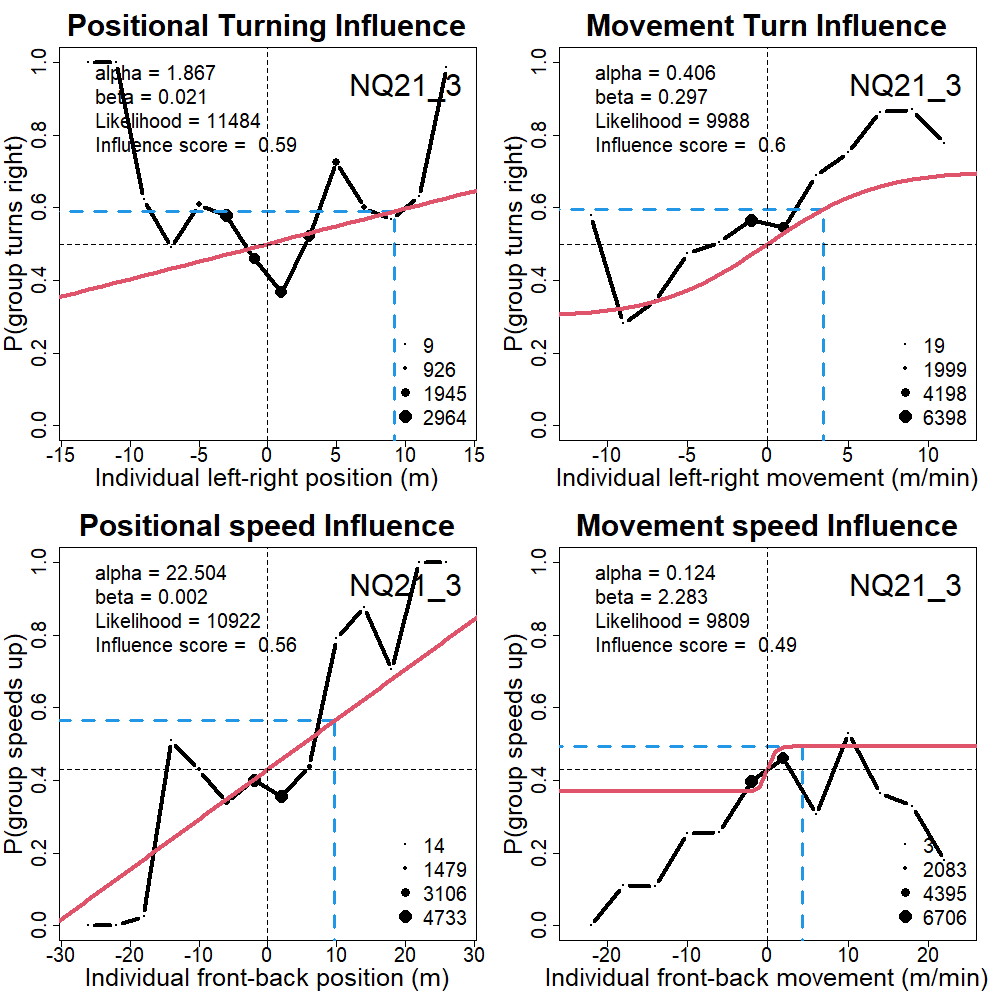
**
